# Are Fear Learning Processes Altered in Obsessive‐Compulsive Disorder, Social Anxiety, and Specific Phobia? Insights From the Late Positive Potential, Fear‐Potentiated Startle, and Ratings

**DOI:** 10.1111/psyp.70277

**Published:** 2026-03-23

**Authors:** Kim M. Sobania, Kai Härpfer, Hannes P. Carsten, Tania M. Lincoln, Franziska M. Kausche, Anja Riesel

**Affiliations:** ^1^ Department of Psychology University of Hamburg Hamburg Germany

**Keywords:** fear conditioning, late positive potential (LPP), OCD, social phobia, specific phobia, startle response

## Abstract

Fear learning processes are often considered underlying mechanisms in the development and maintenance of anxiety‐ and stress‐related disorders. However, limited attention has been paid to whether these changes are shared across disorders or certain symptoms. In this context, transdiagnostic research on symptom dimensions is especially relevant, as it addresses the significant symptom overlap and heterogeneity observed in these disorders. In the current study, we investigated the late positive potential, fear‐potentiated startle, and subjective ratings (US‐expectancy) in a transdiagnostic sample (*N* = 156) including participants with obsessive‐compulsive disorder (OCD; *n* = 38), social anxiety disorder (SAD; *n* = 39), specific phobia (SP; *n* = 40), and control participants (*n* = 39). Anxious arousal, anxious apprehension, and depressive symptoms were examined as relevant core symptom dimensions to these disorders. A differential fear learning paradigm using geometrical forms was employed, including a habituation, acquisition, generalization, and extinction phase. We observed successful fear acquisition across all outcomes, which generalized to the stimulus most similar to the CS+, the GS+. During extinction, fear responses to the CS+ remained significantly elevated compared to the CS−. Group comparison revealed that patients with SAD rated US‐expectancy for the CS− higher during acquisition. On a dimensional level, anxious arousal was associated with increased US‐expectancy and startle response for the CS+ during acquisition and increased US‐expectancy for the GS+ during generalization. Depressive symptoms were linked to an overall higher US‐expectancy during extinction. These findings suggest that individual differences in symptom dimensions, particularly anxious arousal and depressive symptoms, appear to influence fear learning and extinction processes across disorders. This underscores the potential of adopting transdiagnostic approaches in future research and clinical interventions and highlights that the frequently co‐occurring comorbid depressive symptoms impact fear extinction, and therefore, should be considered in the treatment of anxiety disorders to enhance therapeutic outcomes.

## Impact Statement

This study investigates fear and safety learning across different anxiety‐ and stress‐related disorders (obsessive‐compuslive disorder, social anxiety disorder, and specific phobia) while also considering core symptom dimensions to address symptom overlap and heterogeneity. Results indicate that a dimensional approach across disorders is more effective than categorical comparisons, with transdiagnostic dimensions—especially anxious arousal and depressive symptoms—significantly influencing fear learning and extinction. This highlights the importance of adopting transdiagnostic approaches in future research and clinical interventions.

## 1 Introduction

Adequate fear learning is an important requirement to protect individuals from potentially threatening stimuli and situations. In the laboratory, this process can be examined using fear conditioning paradigms, most of which consist of multiple phases, which commonly include fear acquisition, fear generalization, and fear extinction learning (Lonsdorf et al. 2017). Fear acquisition involves associative learning, where a neutral stimulus (NS) becomes a conditioned stimulus (CS+) after being repeatedly paired with an aversive event (US; Duits et al. 2015; Lonsdorf et al. 2017; Pavlov 1927). Additionally, another stimulus that is never paired with the US serves as a safety cue (CS−; Duits et al. 2015; Fyer et al. 2020). Thus, differential conditioning involves excitatory learning to the CS+ as well as inhibitory learning to the CS− (Bouton 2004; Lonsdorf and Merz 2017). Fear generalization refers to the process by which individuals apply previously acquired fear associations and responses to perceptually or semantically similar stimuli (GS) without the need for relearning (Cooper et al. 2022; Dymond et al. 2015; Lissek et al. 2008). This phenomenon allows for a quicker response to potential threats, but it can also lead to undue anxiety in situations that may not pose an actual threat (Dymond et al. 2015). In contrast, fear extinction entails an inhibitory learning process, where individuals learn that a previously fear‐associated stimulus no longer signals danger or threat, by repeatedly presenting the CS+ without the US (Bouton 1993; Craske et al. 2014; Hermans et al. 2006). Differential fear conditioning in humans is commonly examined using various outcomes (Lonsdorf et al. 2017; Ojala and Bach 2020). The most frequently used are self‐report ratings (e.g., ratings of US‐expectancy), the fear‐potentiated startle (FPS), and skin conductance response (SCR) (e.g., Boddez et al. 2013; Hamm and Weike 2005; Lissek et al. 2008; Vervliet et al. 2013). More recently, event‐related potentials (ERPs) have gained increased attention (Lonsdorf and Merz 2017). The FPS serves as a reliable and fast expression of a defensive response (Blumenthal et al. 2005; Lang et al. 1990). Triggered by auditory startle probes it can be measured with electromyography at the orbicularis oculi muscle (Blumenthal et al. 2005; Hamm and Vaitl 1996; Lipp et al. 1994; Lonsdorf et al. 2017). In fear conditioning paradigms, increased FPS responses are found during the presentation of the CS+ compared to the CS− (e.g., Andreatta and Pauli 2015; Lissek et al. 2008). ERPs, on the other hand, indicate electrophysiological correlates of the processing of fear‐relevant stimuli and related attentional processes (Öhmann et al. 2001). The late positive potential (LPP) as a late slow wave ERP component has been associated with in‐depth processing of emotional or motivational salient stimuli (Cuthbert et al. 2000; Hajcak et al. 2012). LPPs during fear conditioning are reported to be elevated for the CS+ compared to the CS− during fear acquisition and generalization (e.g., Bacigalupo and Luck 2017; Bruchmann et al. 2021; Dou et al. 2023; Paiva et al. 2020).

While fear learning processes are essential for survival, studies suggest that these often exceed a functional level in patients with anxiety and stress‐related disorders such as social anxiety disorder (SAD), obsessive‐compulsive disorder (OCD) or post‐traumatic stress disorder (PTSD), resulting in suffering and impairment in everyday life (Cooper et al. 2022; Kausche et al. 2025; Lissek et al. 2008). Mechanisms of fear learning have been linked to symptom development, maintenance, and treatment of those disorders (Abramowitz et al. 2009; Duits et al. 2015; Lissek et al. 2005). Furthermore, previous studies indicate that especially fear extinction processes are associated with exposure‐based therapy outcomes, the standard treatment for anxiety‐ and stress‐related disorders (e.g., Arch and Craske 2009; Geller et al. 2019; Rosenberg et al. 2024; Scheveneels et al. 2021). Meta‐analytical evidence on fear conditioning in anxiety‐ and stress‐related disorders (i.e., anxiety disorders, OCD, and PTSD) reveals that patients with these disorders, when compared to healthy controls (HC), exhibit heightened fear responses to conditioned safety cues during fear acquisition and reduced fear extinction (Duits et al. 2015; Kausche et al. 2025). More precisely, for the safety cue, results demonstrated a heightened reactivity during acquisition, extinction, and extinction recall in subjective ratings and FPS data (Kausche et al. 2025). Additionally, Cooper et al. (2022), provide meta‐analytic evidence for a small positive effect in favor of heightened fear generalization in anxiety‐ and stress‐related disorders relative to comparison participants. In general, these changes in fear acquisition, generalization, and extinction were observed across various anxiety and stress‐related disorders. However, there is also evidence for disorder‐ or symptom‐specific effects such as, for example, that only anxiety and OCD patients but not PTSD patients show elevated affect ratings to the CS+ compared to HC (Kausche et al. 2025). Research on different disorders shows differential results regarding fear and safety learning supporting disorder‐ or symptom‐specific effects, but comparability is limited due to methodological differences and few studies examine multiple disorders simultaneously.

Initial primary studies have shown that individuals with OCD and those with SAD exhibit altered fear acquisition compared to HC (e.g., Geller et al. 2017; Rabinak et al. 2017). More precisely, OCD patients, when compared to HC, demonstrated a more pronounced fear response to the CS+ (Geller et al. 2017) or the CS− (Apergis‐Schoute et al. 2017; Fyer et al. 2020), whereas SAD patients exhibited increased ratings for both the CS+ and the CS− (Hermann et al. 2002; Lissek et al. 2008; Rabinak et al. 2017). For processes of fear generalization, previous studies have been inconsistent. While some studies have shown overgeneralization, that is, increased responding to the CS− and GS in patients with panic disorder and generalized anxiety disorder (GAD) compared to HC (Lissek et al. 2010, 2014), other studies reported no difference between GAD patients and HC (Tinoco‐González et al. 2015). In addition, overgeneralization was not reported for patients with specific phobia (SP) (Lange et al. 2019) and Ahrens et al. (2016) reported overgeneralization of conditioned fear in SAD patients for socially relevant stimuli, as reflected in SCR and arousal ratings, though this effect did not extend to US expectancy or valence ratings. For, patients with panic attacks fear overgeneralization was reported in one study (Lissek et al. 2010). Lastly, studies showed impaired extinction learning in patients with SAD (Hermann et al. 2002) and panic disorder (Neueder et al. 2019; Schwarzmeier et al. 2019), but not in SP (Lange et al. 2019) and mixed results for OCD patients (Cooper and Dunsmoor 2021). While a substantial amount of previous studies in the field of anxiety‐ and stress‐related disorders have operationalized fear learning processes using subjective ratings, SCR, and FPS, only few have assessed the LPP: Klein et al. (2024) focused on the comparison between clinically anxious and non‐anxious youth in a fear generalization and extinction recall paradigm. They found a significant enhanced LPP for the CS+ as well as the stimulus most similar to the CS+ compared to the CS− during fear generalization in anxious but not in non‐anxious youth. Another study investigated fear conditioning using the P300 in participants with and without PTSD using a trauma‐specific picture as US. They reported an overall increased response to the CS+ versus the CS− during acquisition and extinction in participants with PTSD (Wessa and Flor 2007).

Taken together, evidence supports that patients with anxiety disorders show a small but consistently increased fear response to both the CS+ and CS− during fear acquisition compared to HC, while patients with OCD show an increased response to the CS+ only (Geller et al. 2017; Kausche et al. 2025). For overgeneralization and extinction of fear, there is a small but less consistent indication of heightened fear generalization and reduced fear extinction in both anxiety disorders and OCD (Cooper et al. 2022; Kausche et al. 2025). However, considerable symptomatic heterogeneity, symptom overlap and comorbidity between different anxiety‐ and stress‐related disorders, as well as a possible publication bias, have to be taken into account when interpreting these findings (Cooper et al. 2024; Kausche et al. 2025). There are indications of symptom‐ and disorder‐specific differences, but they are difficult to capture meta‐analytically and challenging to integrate across primary studies with varying methodologies. In fact, Cooper and Dunsmoor (2021) suggest that the category of anxiety‐ and stress‐related disorders may not account for differences in fear learning processes and point to potential benefits by focusing on individual differences in transdiagnostic dimensional measures. Both the heterogenous results and the indication of differences between disorders underline the need to investigate differences in fear learning across the diagnostic spectrum of anxiety‐ and stress‐related disorders simultaneously using one single paradigm and multiple outcome measurements (Duits et al. 2015; Kausche et al. 2025). Additionally, examining transdiagnostic symptom dimensions might add to a better understanding of underlying shared and distinct processes within the spectrum of anxiety‐ and stress‐related disorders (Cooper et al. 2024; Sep et al. 2019).

In order to investigate anxiety‐ and stress‐related disorders using a dimensional approach, recent research has characterized anxiety disorders and OCD on two latent dimensions: anxious arousal and anxious apprehension (Clark and Watson 1991; Cox et al. 2010; Krueger 1999; Sharp et al. 2015). The physiological component of anxiety‐ and stress‐related disorders (i.e., hypervigilance, hyperarousal) is thought to be represented by the dimension anxious arousal, whereas anxious apprehension is associated with a more cognitive component (i.e., negative and repetitive thoughts as well as worry) (Härpfer et al. 2021; Sharp et al. 2015). As depressive disorders are the most common comorbidity of anxiety disorders and OCD (Saha et al. 2021) and anxiety symptoms are related as well as distinct to symptoms of depression (Craske et al. 2009), alterations in fear conditioning processes due to depressive symptoms should be considered. In fact, a recent meta‐analysis by Kausche et al. (2025) reports moderating influences of depressive symptoms in patients with anxiety‐ and stress‐related disorders during acquisition and extinction. Thus, having a closer look at different anxiety disorders and OCD as well as applying dimensional approaches—both on the anxiety and the depressive spectrum—to better capture individual differences may help us to better understand their development and maintenance and may offer insights into why not all patients respond to exposure therapy (Foa and Kozak 1985; Hofmann and Smits 2008; Milad and Quirk 2012; Rosenberg et al. 2024).

In light of the scarcity of previous studies, our preregistered^1^ (osf.io/cfqm9) study aimed to explore fear conditioning processes in a transdiagnostic sample of patients with different anxiety disorders (i.e., SAD and SP), OCD, and unaffected controls. These diagnostic groups were chosen as SP is characterized primarily by circumscribed fear with strong anxious arousal, OCD by pronounced anxious apprehension, and SAD by a combination of both dimensions (Clark and Watson 1991; Cox et al. 2010; Krueger 1999; Sharp et al. 2015). Specifically, while we recruited the sample based on their diagnoses, the aim was, however, to integrate categorical analyses based on diagnoses with dimensional analyses based on symptom dimensions. To this end, we used multiple outcome measurements—specifically shock expectancy ratings, FPS, and LPP—as well as different fear learning phases including fear acquisition, generalization, and extinction to assess the various facets of the underlying processes across different diagnostic groups. We preregistered hypotheses regarding categorical as well as dimensional analyses and all hypotheses relate equally to the main outcome variables. For the categorical analyses, we hypothesized that during fear acquisition all groups would show differential fear acquisition. We further hypothesized that, whereas participants with SAD and SP would show increased responding to the CS+ and CS− compared to controls, participants with OCD would show increased responding only to the CS+ compared to controls. For fear generalization we hypothesized that all groups would show fear generalization and that participants with SAD, SP, and OCD would show increased fear generalization compared to controls. Lastly, for fear extinction we hypothesized that all participants would show fear extinction and that those with SAD, SP, and OCD would show reduced fear extinction compared to controls. For the dimensional analyses, we hypothesized that during fear acquisition, anxious arousal would be associated with enhanced responding towards the CS+ and CS−, and anxious apprehension would be associated with enhanced responding towards the CS+. During fear generalization, anxious arousal would be associated with enhanced responding to the generalization stimuli, and anxious apprehension primarily with enhanced responding to the most ambiguous generalization stimulus, that is, the stimulus equally similar to the CS+ as well as CS−. Lastly, during fear extinction, anxious arousal as well as anxious apprehension would be associated with delayed fear extinction.

## 2 Methods

### 2.1 Participants

The final sample consisted of *n* = 156 participants (*n* = 39 control participants, *n* = 38 patients with OCD, *n* = 40 patients with SP, *n* = 39 patients with SAD). Groups were matched for self‐identified gender (*n* = 118 female [75.64%], *n* = 38 male [24.36%]), age (*M* = 28.44, SD = 7.98), and years of education (*M* = 12.17, SD = 0.98), see Table 1 for demographic and clinical characteristics. Initially, 163 participants were invited to the laboratory assessment, but five participants were excluded due to experiment discontinuation and another two participants were mistakenly included in the study even though they met an exclusion criterion. Presence of current psychiatric diagnoses was assessed in all participants using the Structured Clinical Interview for DSM‐5 Disorders—Clinical Version (SCID‐5‐CV; Beesdo‐Baum et al. 2019; First et al. 2016). Comorbidities within the anxiety‐ and stress‐related disorder spectrum were allowed. Participants were assigned to the clinical groups based on their primary diagnosis. Exclusion criteria were: history of any neurological disorder, current or lifetime substance‐related disorders and schizophrenia spectrum disorder, current manic episode in the context of a bipolar disorder, use of benzodiazepines during the last week, and neuroleptic medication during the last 3 months. Further exclusion criteria for the control group included: current or lifetime diagnosis of SP, SAD, and/or OCD. In order to recruit a realistic community control group, participants with any other current diagnoses of mental disorders were not excluded. In total, three participants in the control group fulfilled criteria for a current mental disorder (i.e., skin‐picking disorder, premenstrual dysphoric disorder, and primary insomnia). None were diagnosed with a current anxiety‐ or stress related nor with a current affective disorder.

**TABLE 1:** Demographical, questionnaire, and clinical data across diagnostic groups.

	OCD_a_ (*n* = 38)		SAD_b_ (*n* = 39)		SP_c_ (*n* = 40)		CON_d_ (*n* = 39)		Group comparison			
	*M*	SD	*M*	SD	*M*	SD	*M*	SD	*χ* ^2^/*F*	df	$\eta_{p}^{2}$	*p*
Demographics												
Gender (f/m)	29/9		29/10		31/9		29/10		0.15	3		0.985
Age	29.13	7.55	28.74	7.67	27.63	9.72	28.28	6.86	0.25	3, 152	0.01	0.858
Education	12.11	1.03	12.36	0.87	12.10	1.03	12.13	0.98	0.63	3, 152	0.01	0.598
Questionnaires												
OCI‐R	29.03_bcd_	11.39	11.54_ad_	8.86	9.28_a_	10.07	5.51_ab_	4.74	49.54	3, 150	0.50	**< 0.001**
LSAS‐SR	46.65_bd_	29.89	71.46_acd_	28.76	32.24_b_	23.91	20.90_ab_	17.64	28.57	3, 149	0.37	**< 0.001**
SMSP	8.24_d_	8.65	6.54_d_	7.39	8.87_d_	7.79	1.74_abc_	3.79	7.74	3, 148	0.14	**< 0.001**
PSWQ	57.97_cd_	11.84	57.77_cd_	9.05	48.68_abd_	13.73	39.90_abc_	11.43	21.27	3, 152	0.30	**< 0.001**
MASQ‐AA	29.16_d_	9.55	28.13_d_	7.93	24.55	8.11	21.95_ab_	5.96	6.70	3, 152	0.12	**< 0.001**
STAI‐T	52.14_cd_	11.60	53.36_cd_	10.04	39.77_ab_	12.39	35.10_ab_	7.63	28.40	3, 150	0.36	**< 0.001**
STAI‐S	44.78_cd_	13.51	45.53_cd_	10.37	34.97_ab_	9.23	32.53_ab_	5.91	16.54	3, 148	0.25	**< 0.001**
BDI‐II	17.11_cd_	12.12	16.97_cd_	12.51	8.08_ab_	9.57	3.67_ab_	4.53	16.72	3, 150	0.25	**< 0.001**
Clinical												
Number of diagnoses	3.05_cd_	1.90	3.13_cd_	2.08	1.98_abd_	1.53	0.13_abc_	0.34	29.32	3, 152	0.37	**< 0.001**
Comorbid depression (yes/no)	8/30		12/27		4/36		0/39		16.01	3		**0.001**
Antidepressants (yes/no)	9/29		4/35		2/38		0/39		13.80	3		**0.003**
CGI‐S	4.63_cd_	0.88	4.58_cd_	1.03	3.80_abd_	0.99	1.97_abc_	0.63	73.03	3, 151	0.60	**< 0.001**
GAF	55.79_cd_	9.25	58.34_cd_	11.44	75.40_abd_	11.41	88.74_abc_	6.37	95.28	3, 151	0.65	**< 0.001**
YBOCS	22.39	5.55	—	—	—	—	—	—	—	—	—	—

*Note:* Degrees of freedom are deviating for some questionnaires due to missing data; means with different subscripts within rows indicate significant differences between the groups (the letters indicate to what group a significant difference was found with a = OCD, b = SAD, c = PHOB, d = CON) according to Sidak corrected post hoc *t*‐tests with *p* < 0.05. *N* = 156. *p* < 0.05 are printed in bold.

Abbreviations: BDI‐II = Beck Depression Inventory II; Diagnoses include index and comorbid diagnoses (*n*); CGI‐S = clinical global impression—severity of illness, CON = control group; Self‐identified gender (f = female, m = male); age and education in years, GAF = GAlobal assessment of functioning, LSAS‐SR = Liebowitz Social Anxiety Scale—self report, MASQ‐AA = Mood and Anxiety Symptom Questionnaire (Anxious Arousal Subscale), OCD = obsessive‐compulsive disorder, OCI‐*R* = obsessive‐compulsive inventory, PSWQ = Penn State Worry Questionnaire, SAD = social anxiety disorder, SMSP = severity measure for specific phobia, SP = specific phobia, STAI = state–trait‐anxiety inventory (trait and state subscale), YBOCS = Yale‐Brown Obsessive Compulsive Scale (was only conducted when individuals had an OCD diagnosis).

### 2.2 Procedure

The study was approved by the local ethics committee of the University of Hamburg (AZ: 2021_351). Participants were recruited via the outpatient unit of the University of Hamburg and external advertisements. Prior to the laboratory assessment, participants were screened for eligibility and conducted a telephone interview to assess clinical diagnoses. During the laboratory assessment, participants completed questionnaires (see Table 1) and performed a series of tasks relevant to a larger project. These tasks were performed in the order in which they are listed: a resting state measurement, a flanker task, a passive picture viewing task, a No Threat/Predictable Threat/Unpredictable Threat task, as well as the differential fear conditioning task that is the focus of this study.

#### 2.2.1 Questionnaires

The battery of questionnaires included various facets of clinical symptom dimension (see Supporting Information: Appendix B for an overview). Anxious apprehension was measured by the Penn State Worry Questionnaire (PSWQ; 16 items, 5‐point Likert scale 1–5; Cronbach's *α* = 0.94; Glöckner‐Rist and Rist 2014; Meyer et al. 1990), anxious arousal by the respective subscale of the Mood and Anxiety Symptom Questionnaire (MASQ‐AA; 17 items, 5‐point Likert scale 1–5; Cronbach's *α* = 0.88; Watson et al. 1995; Watson and Clark 1991), and depression symptoms by the Beck Depression Inventory II (BDI‐II; 21 items, 4‐point Likert scale 0–3; Cronbach's *α* = 0.95; Beck et al. 1996; Hautzinger et al. 2006). Participants gave written informed consent and received either course credit or monetary compensation for study participation.

#### 2.2.2 Fear Conditioning Task

**FIGURE 1:**
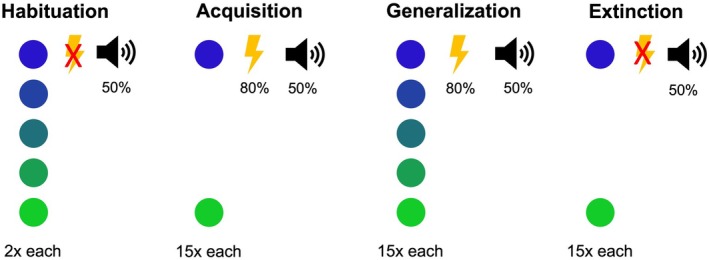
Schematic representation of the differential fear conditioning task. Overview of the four phases during the differential fear conditioning task. During Habituation, the startle probe was presented first, five times without the presentation of the conditioned stimuli (CS; colored circles), followed by the CS two times each. An unpleasant but not painful electrotactile shock to the back of the dominant hand (flash symbol) served as unconditioned stimulus (US). During Acquisition the CS+ and the CS− were presented 15 times each, with a reinforcement rate of 80%. During Generalization the five CS were presented 15 times each with a reinforcement rate of 80%. During Extinction the CS+ and CS− were presented 15 times each and no US was applied. During the whole task, the startle probe (speaker symbol) was delivered during 50% of the CS trials and 50% of the inter‐trial intervals (ITIs). The color of the CS+ was counterbalanced across participants. The duration of the stimuli presentation was 2600 ms, the duration of the ITIs was counterbalanced between 1500 and 2500 ms. US onset was 2450 ms after CS onset and startle onset 600 ms prior to CS or ITI offset.

The differential fear conditioning task (see Figure 1) was displayed using Presentation Software (Neurobehavioral Systems Inc., Albany, California) on a 19‐in. monitor with a resolution 1920 × 1080 pixels and a refresh rate of 120 Hz. Participants sat in a dimly lit, electrically shielded cabin at a viewing distance of approximately 24 in. The task consisted of four different phases: habituation, fear acquisition, fear generalization, and fear extinction. These phases were conducted consecutively and without any breaks. Five colored circles with a resolution of 172 × 172 pixels (visual angle *α* = 4.5°) served as conditioning stimuli (CS). The color of the circles varied along a blue to green dimension. The bluest and greenest stimuli served as CS+ and CS−, counterbalanced across participants. The stimuli in between served as generalization stimuli (GS). One GS was most similar to the blue circle, one most similar to the green circle, and one GS was the most ambiguous stimulus, incorporating equally blue and green color (see Supporting Information: Appendix C for an overview of the color coding). An electrotactile shock to the back of the dominant hand served as the US. Prior to the experiment, a shock workup procedure was conducted: First, the individual perception threshold was determined, followed by a gradual increase in intensity to identify a level that was perceived as unpleasant but not painful. During fear acquisition and generalization, the reinforcement rate was 80%. During extinction learning, no US was applied. CS presentation was always 2600 ms, with an inter‐trial interval (ITI) of 1500–2500 ms. US followed CS presentation after 2450 ms, terminating with CS offset. The approximate total duration of the task was 15–20 min.

To measure the FPS, a startle sound was delivered in 50% of the CS trials as well as during 50% of the ITIs, always 600 ms before CS or ITI offset. Startle probes consisted of 95 dB bursts of white noise (50 ms, rise/fall time < 1 ms) delivered binaurally through headphones. Prior to the presentation of the CS during the habituation phase, five noise alone trials were delivered for habituation purposes towards the startle probe (Blumenthal et al. 2005).

After every phase, participants rated all CS according to their US‐expectation, the unpleasantness of the US, and the unpleasantness of the startle probe on a visual analog scale from 0 to 100.

### 2.3 Electrophysiological Recording and Preprocessing

Using 61 Ag/AgCl‐electrodes mounted on a cap with equidistant electrode sites (Easycap, Herrsching, Germany) and two 32‐channel BrainAmp amplifiers (Brain Products GmbH, Gilching, Germany), EEG signals were recorded with a band‐pass filter of 0.01–250 Hz and digitized continuously at a sampling rate of 1000 Hz. Recording reference was located between AF3 and Fz, the ground electrode between AF4 and Fz. An external electrode was placed at the left infraorbital site for vertical eye movements. EEG data were processed in *Brain Vision Analyzer 2.2.2* (Brain Products GmbH, Gilching, Germany). First, a band‐pass filter with a low cut‐off of 0.1 Hz and a high cut‐off of 30 Hz (24 dB/oct roll‐off) as well as a notch filter of 50 Hz were applied to the continuous EEG signals. Subsequently, ocular artifacts were corrected by using an independent component analysis (ICA; Jung et al. 2000). Relevant components were semi automatically identified and manually checked by visual inspection of the scalp topography, the component activation, and the inspection of the corrected EEG signal. Continuous data then were re‐referenced to the average of all scalp electrodes and segmented into response‐locked epochs (CS: −200 ms to 2600 ms) and startle probe‐locked epochs (Startle: −50 ms to 500 ms). Segments containing artifacts (i.e., absolute voltage range exceeding 200 μV, voltage step exceeding 50 μV between consecutive data points, or maximum voltage difference of less than 0.5 μV within 100 ms intervals) were removed. Since our study involves a unique clinical sample and longer ERPs, such as the LPP, which may be more susceptible to artifacts, we preregistered to exclude participants from data analysis if more than 50% of all trials contained artifacts (Luck 2014). Notably, however, the highest percentage of trial rejection in our data was only 14.82% (20 trials), indicating high data quality (range of rejected trials across participants = 0–20, *M* = 1.97, SD = 3.24) and demonstrating that our data also meet the recommended 25% cutoff for an ideal signal‐to‐noise ratio (Luck 2014). EEG data was corrected for the baseline interval of −200 to 0 ms. Finally, the LPP was quantified as the mean activity at the Pz electrode between 300 ms until 1000 ms (e.g., Cuthbert et al. 2000; Foti et al. 2009; Hajcak and Olvet 2008; Weinberg and Hajcak 2011).

For the FPS, two Ag/AgCl electrodes with a sensor diameter of 6 mm were placed directly below the lash line of the right eyelid, beneath the pupil and adjacent to it, to measure the surface electromyography of the eyeblink component of the startle reflex, in accordance with the guidelines by Blumenthal et al. (2005). A ground electrode was placed on the right infraorbital triangle. Data were recorded with a sampling rate of 1000 Hz. The data were preprocessed using filtering (28–500 Hz, 24 dB/octave roll‐off), rectification, baseline correction (baseline = 50 ms prior to startle probe onset), and integration (9 ms moving average). For each stimulus in each phase, segments from −500 to 250 ms relative to the startle probe were extracted. This segmentation was followed by detecting segments with excessive noise or spontaneous blinks that were scored as “missing value.” This detection took place automatically using the EMG Onset Search macro implemented in *Brain Vision Analyzer 2.2.2*. In a time window of −50 to 20 ms (relative to startle probe, i.e., refractory period), deviations ≥ 4 SD from either of two baselines (−500 to −300 ms and −300 to −100 ms relative to startle probe) were scored as “missing value” and omitted from analyses and further detection of startle blinks. Two baselines were used to avoid spontaneous blinks in either baseline period that could result in the inability of the algorithm to pick up blinks in the refractory period. Using the same algorithm, startle probe onset 20–120 ms after startle probe, was determined by deviations ≥ 4 SD from the baseline −50 to 0 ms relative to startle probe and the highest peak was scored in a 25–150 ms window relative to the startle probe. Undetectable responses (i.e., segments that are neither “missing values” nor contain > 4 SD baseline deviations in the response time window) were scored as zero and included into the analyses (i.e., startle magnitude, cf. Blumenthal et al. 2005). To ensure the availability of sufficient startle reactions, participants were excluded from the startle analyses if they showed zeros or “missing values” to > 66% of the startle probes including the 5 startle trials presented in the initial startle habituation phase (cf. Kuhn et al. 2020). Using *R Studio*, Version 2024.04.2 (R Core Team 2023; Posit Team 2024), data were t‐standardized for all analyses except linear mixed‐effects models where raw single trial data were used.

### 2.4 Data Analysis

For statistical analyses, *IBM SPSS Statistics*, Version 29.0.2.0 (SPSS Inc., Chicago), and *R Studio*, Version 2025.05.1 (R Core Team 2023; Posit Team 2025) were used. A significance level of *α* = 0.05 was used for all analyses. For post hoc comparisons, *p*‐values were Sidak corrected. Separate univariate analyses of variance (ANOVAs) were used to examine differences in demographical and clinical variables.

Regarding data analysis we deviated from the preregistration in the following respects. First, analysis of SCR was not included as we did not observe reasonable task effects, probably due to the paradigm being optimized for EEG and FPS data. Second, for LPP, single‐trial FPS, and shock (i.e., US) expectancy ratings, mixed‐measured ANOVAs for categorical hypotheses and separate regression models for dimensional hypotheses were preregistered. Instead, linear mixed‐effects models using the *lme4* (version 1.1.37; Bates et al. 2015) and *lmerTest* (version 3.1.3; Kuznetsova et al. 2017) package in *R* were fitted in order to reduce the number of separate analyses and to allow for simultaneous testing of both categorical and dimensional predictors. We would like to emphasize that the preregistered analyses (outlined in Supporting Information: Appendix D) were also carried out. The convergence and differences with the reported models are presented in Section 3.6. Third, additional exploratory analyses were added that are described below (see also Supporting Information: Appendix A for a detailed list of deviations from preregistration as well as reasons).

For the series of linear mixed‐effects models, the dependent variable (DV) was modeled as a function of fixed effects and subject‐level random intercepts. First, a null model was specified, including only a random intercept for participant (ID), to estimate the proportion of variance attributable to between‐subject differences:

$$\mathrm{DV}∼1+\left(1\;|\;\mathrm{ID}\right)$$

For the shock expectancy ratings for the acquisition and generalization phase, the null model exhibited singular fit, likely due to limited variance in the random effects. After comparing the pattern of results to the preregistered analyses and in order to preserve model consistency across outcomes, the full random‐effects structure was retained. However, results for these two models should be interpreted more cautiously, as parameter estimates may be less reliable (Field 2024, 1002). Next, a full model was computed to test the effects of stimulus cue (i.e., CS+ and CS− for acquisition and extinction, and CS+, GS+, GSU, GS− and CS− for generalization), group (i.e., four levels: OCD, SAD, SP, control), and three mean‐centered continuous predictors (i.e., anxious apprehension measured with the PSWQ, anxious arousal measured with the MASQ‐AA, and depressive symptoms measured with the BDI‐II; in contrast to anxiety dimensions, dimensional analyses of depressive symptoms were not preregistered but exploratorily included as recent meta‐analytic findings suggest an influence of depressive symptoms on altered fear learning [Kausche et al. 2025], see Supporting Information: Appendix A) as well as their full interaction with stimulus cue and group:

$$\begin{array}{c} \mathrm{DV}∼\text{stimulus}\;\mathrm{cue}+\text{group}+\text{PSWQ}+\text{MASQ}‐\mathrm{AA}\\ +\mathrm{BDI}‐\mathrm{II}+\text{stimulus}\;\mathrm{cue}\times \left(\text{group}+\text{PSWQ}+\text{MASQ}‐\mathrm{AA}+\mathrm{BDI}‐\mathrm{II}\right)\\ +\text{group}\times \left(\text{PSWQ}+\text{MASQ}‐\mathrm{AA}+\mathrm{BDI}‐\mathrm{II}\right)\\ +\left(1\;|\;\mathrm{ID}\right) \end{array}$$

The model for the shock expectancy rating during the generalization phase was computed without the BDI‐II due to multicollinearity, influences of the BDI‐II were checked separately. Results indicated no significant influence of BDI‐II levels on shock expectancy ratings during generalization and can be found in Supporting Information: Appendix E. Model summaries, degrees of freedom, test statistics, and standard errors were extracted using the *lmerTest* (version 3.1.3; Kuznetsova et al. 2017), *parameters* (version 0.26.0; Lüdecke et al. 2020), and *sjPlot* (version 2.8.17; Lüdecke 2024) packages. Model fit was evaluated using marginal and conditional *R* ^2^ values, and standardized effect sizes (partial *R* ^2^) were calculated using the *r2glmm* (version 0.1.3; Jaeger 2025) package. To identify the most parsimonious model, a backward model selection procedure, starting from the full model was applied. For these resulting reduced models, significance testing of fixed effects was performed using a Type III ANOVA with Satterthwaite's method to obtain *F*‐statistics and *p*‐values. For post hoc comparisons, estimated marginal means (EMMs) and conditional trends were computed using the *emmeans* (version 1.11.1; Lenth 2025) package.

Fear generalization was defined as an increase in fear response with increasing similarity to the threat associated CS+. This was analyzed via EMMs between the CS+ and any of the GS (i.e., GS+ as the stimulus most similar to the CS+, GS− as the stimulus most similar to the CS−, and GSU as the most ambiguous stimulus, incorporating equally similar parts to the CS+ as well as CS−). As there are numerous indices to quantify fear generalization (Stegmann et al. 2025), we additionally generated a fear generalization index (GI) by normalizing the mean response of the GS for responding to the CS+ and compared that between groups using an ANOVA:

$$\mathrm{GI}=\Sigma \left(\mathrm{GS}+,\mathrm{GSU},\mathrm{GS}-\right)\;\mathrm{CS}+$$

Additional non‐preregistered analyses were conducted. First, in order to test for changes across phases, one linear mixed‐effect model for each outcome measure was carried out including the factor phase (i.e., habituation, acquisition, generalization, and extinction for shock expectancy rating as well as acquisition, generalization, and extinction for FPS and LPP data), stimulus cue (i.e., CS+ and CS− only), and group (i.e., OCD, SAD, SP, and control):

$$\mathrm{DV}∼\text{stimulus}\;\mathrm{cue}\times \text{phase}\times \text{group}+\left(1\;|\;\mathrm{ID}\right)$$

Sidak corrected EMMs were used for post hoc comparisons. Second, in order to investigate group differences regarding shock and startle valence, two non‐preregistered mixed‐measures ANOVAs were calculated with the between‐subject factor group and the within‐subject factor phase (i.e., acquisition and generalization for shock valence, and acquisition, generalization, and extinction for startle valence). Third, we conducted Bayesian analyses for the main outcome measures to better parameterize our observed null effects. Lastly, we split up our sample in different clinical categories. Firstly, in two groups to compare individuals with any current anxiety‐ or stress‐related disorder (i.e., anxiety disorders, OCD, PTSD) to individuals without such diagnosis. Secondly, in three groups, were we additionally compared individuals with any current anxiety‐ or stress related disorder with a current comorbid depression (i.e., current depressive episode or dysthymia; A+/D+), to individuals with a current anxiety‐ or stress related disorder without a current comorbid depression (A+/D−), and individuals without such diagnoses (A−/D−). After dividing the groups, models were fitted using linear mixed‐models with an identical null model as described above and the following full model:

$$\mathrm{DV}∼\text{stimulus}\;\mathrm{cue}\times \text{group}+\left(1\;|\;\mathrm{ID}\right)$$

Again, Sidak corrected EMMs were used for post hoc comparisons.

## 3 Results

Due to missing data and dropouts, the number of participants varies for each outcome measure. An overview of missing data as well as reasons are listed in Supporting Information: Appendix F.

### 3.1 Demographical, Questionnaire, and Clinical Data

There were no significant differences regarding self‐identified gender, age, or education between the four groups. There were significant differences in the questionnaire and clinical interview scores with higher clinical burden in the clinical groups (see Table 1 for an overview and statistics, and Supporting Information: Appendix G for figures displaying the distribution across groups). According to the assessed questionnaires, obsessive‐compulsive symptoms were most prominent in the OCD group, and social anxiety symptoms were most prominent in the SAD group. While the SP group reported the highest SP symptoms, it was not significantly different from the OCD and SAD groups. Worry, trait, and state anxiety, as well as depressive symptoms, were most pronounced in the OCD and SAD groups, being significantly different from the SP and control groups. Anxious arousal was most prominent in the OCD and SAD groups compared to the control group, with the SP group being in between. The severity of illness in the clinical global impression rating was significantly more severe for the OCD and SAD groups compared to the SP and control groups, followed by the SP group being significantly more severe compared to the control group. A similar pattern was found for the global assessment of functioning, with the control group having the highest level of functioning, followed by the SP group, and then followed by the OCD and SAD groups.

### 3.2 Shock and Startle Valence

For shock valence, there were no significant main effects of phase (*F*(1, 147) < 0.01, *p* = 0.965, $\eta_{p}^{2}$ < 0.001), or group (*F*(3, 147) = 1.47, *p* = 0.224, $\eta_{p}^{2}$ = 0.029), nor a significant interaction effect (*F*(3, 147) = 0.95, *p* = 0.421, $\eta_{p}^{2}$ = 0.019). Analysis of startle valence revealed a significant main effect of group (*F*(3, 147) = 4.59, *p* = 0.004, $\eta_{p}^{2}$ = 0.086). Post hoc pairwise comparison further revealed a significant difference between the SAD and control group (*p* = 0.002), with the control group rating the startle valence as significantly more unpleasant compared to the SAD group. There was no significant main effect of phase (*F*(2, 294) = 0.60, *p* = 0.519, $\eta_{p}^{2}$ = 0.004), or interaction between group and phase (*F*(3, 294) = 0.38, *p* = 0.863, $\eta_{p}^{2}$ = 0.008).

### 3.3 Fear Acquisition

Linear mixed‐effects models were calculated to examine the effects of stimulus cue, group, and continuous symptom measures, including random intercepts for participant to account for interindividual differences. To identify the model that explains the most variance, a backward model selection procedure, starting from the full model, was applied. Model fit indices for the reduced models as well as all main and interaction effects are displayed in Table 2. Backward model selection yielded the following models:

$$\begin{array}{c} \text{Shock Expectancy}∼\text{stimulus}\;\mathrm{cue}+\text{group}+\text{MASQ}‐\mathrm{AA}\\ \;+\text{stimulus}\;\mathrm{cue}\times \text{group}+\text{stimulus}\;\mathrm{cue}\times \text{MASQ}‐\mathrm{AA}\\ \;+\left(1\;|\;\mathrm{ID}\right) \end{array}$$

$$\begin{array}{c} \mathrm{FPS}∼\text{stimulus}\;\mathrm{cue}+\text{MASQ}‐\mathrm{AA}+\text{stimulus}\;\mathrm{cue}\\ \;\times \text{MASQ}‐\mathrm{AA}+\left(1\;|\;\mathrm{ID}\right) \end{array}$$

$$\begin{array}{c} \mathrm{LPP}∼\text{stimulus}\;\mathrm{cue}+\text{group}+\mathrm{BDI}‐\mathrm{II}+\text{stimulus}\;\mathrm{cue}\\ \;\times \text{group}+\text{stimulus}\times \mathrm{BDI}‐\mathrm{II}+\text{group}\times \mathrm{BDI}‐\mathrm{II}\\ \;+\text{stimulus}\;\mathrm{cue}\times \text{group}\times \mathrm{BDI}‐\mathrm{II}+\left(1\;|\;\mathrm{ID}\right) \end{array}$$

**TABLE 2:** Model fit indices and results of the Type III ANOVA with Satterthwaite's method for the acquisition phase.

	$R_{c}^{2}$/$R_{m}^{2}$	ICC	RMSE	*σ*	*F*	df	*p*	$R_{p}^{2}$	95% CI
Shock expectancy rating	0.949/0.943	0.102	9.38	10.02					
Stimulus cue					5552.94	1, 146	**< 0.001**	0.811	0.780, 0.839
Group					1.72	3, 146	0.166	0.041	0.013, 0.102
MASQ‐AA					2.24	1, 146	0.137	0.001	0.000, 0.022
Stimulus cue × Group					3.93	3, 146	**0.001**	0.034	0.010, 0.093
Stimulus cue × MASQ‐AA					6.76	1, 146	**0.010**	0.020	0.001, 0.063
FPS^a^	0.685/0.015	0.680	38.11	39.78					
Stimulus cue					43.48	1, 1599	**< 0.001**	0.020	0.008, 0.035
MASQ‐AA					1.16	1, 144	0.283	0.001	0.000, 0.008
Stimulus cue × MASQ‐AA					9.11	1, 1600	**0.003**	0.004	0.000, 0.013
LPP	0.544/0.087	0.050	0.89	1.12					
Stimulus cue					12.11	1, 141	**< 0.001**	0.001	0.000, 0.023
Group					0.78	3, 141	0.505	0.004	0.001, 0.042
BDI‐II					0.21	1, 141	0.648	0.004	0.000, 0.032
Stimulus cue × Group					0.67	3, 141	0.573	0.004	0.001, 0.041
Stimulus cue × BDI‐II					1.67	1, 141	0.198	0.003	0.000, 0.028
Group × BDI‐II					0.47	3, 141	0.706	0.014	0.003, 0.061
Stimulus cue × Group × BDI‐II					2.72	3, 141	**0.047**	0.016	0.003, 0.064

*Note:* Degrees of freedom are deviating for the outcome measures due to missing data. The full model included stimulus cue (i.e., CS+ and CS− for acquisition), group (i.e., four levels: OCD, SAD, SP, and control), three mean‐centered continuous predictors (i.e., PSWQ, MASQ‐AA, BDI‐II) as well as their full interaction with stimulus cue and group. For each outcome measure, an individual reduced model was computed using a backward model selection procedure. *p* < 0.05 are printed in bold.

Abbreviations: BDI‐II = Beck Depression Inventory II, FPS = fear‐potentiated startle, LPP = late positive potential, $R_{c}^{2}$ = conditional *R* ^2^, $R_{m}^{2}$ = marginal *R* ^2^, $R_{p}^{2}$ = partial *R* ^2^, MASQ‐AA = anxious arousal subscale of the Mood and Anxiety Symptom Questionnaire.

^a^ Single trial data were used for the FPS.

#### 3.3.1 Categorical Analysis

##### 3.3.1.1 Shock Expectancy

**FIGURE 2:**
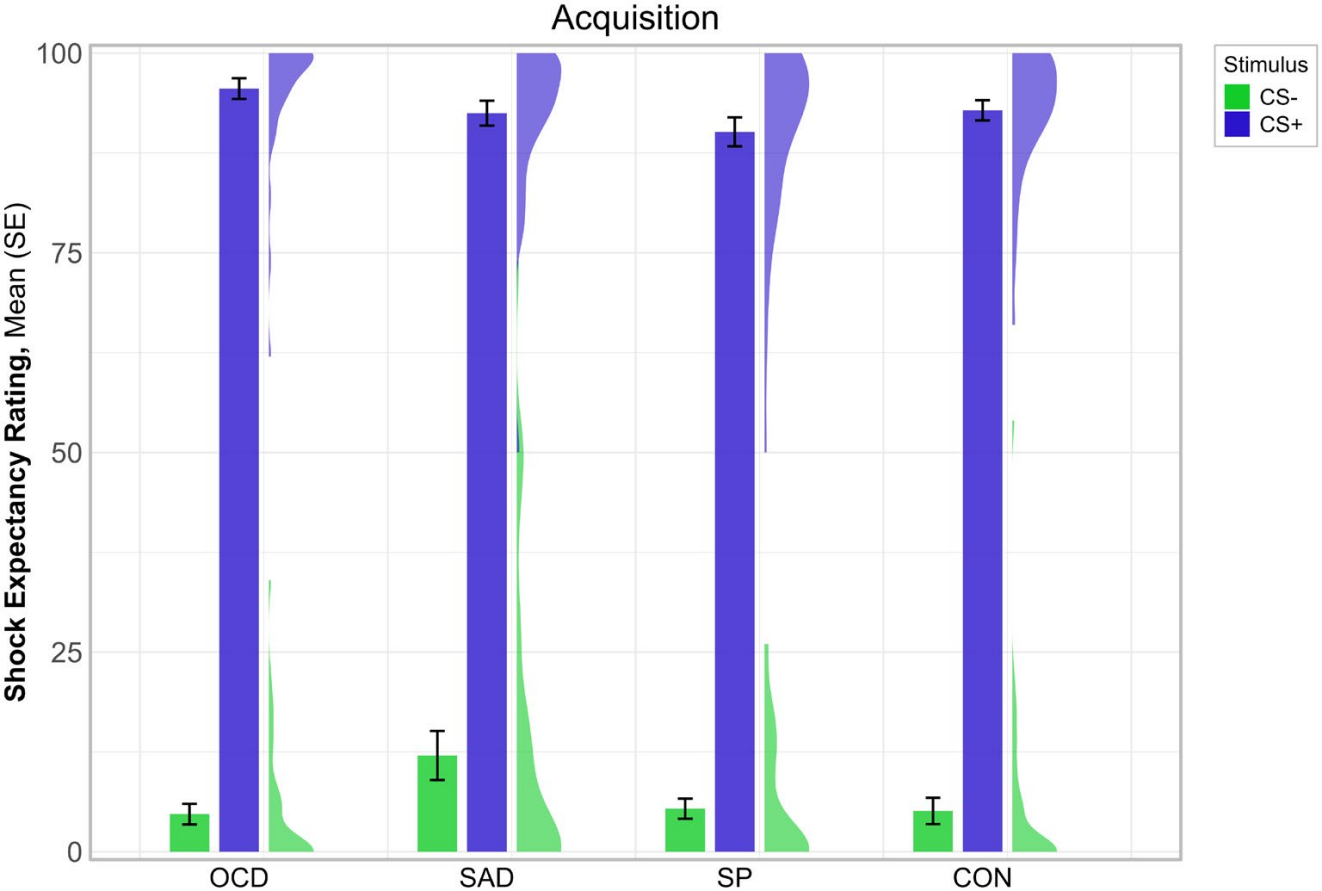
Shock expectancy ratings during acquisition across clinical groups. CON, control group; OCD, obsessive‐compulsive disorder; SAD, social anxiety disorder; SP, specific phobia. Depicted are the mean and the standard error of the shock expectancy ratings during the acquisition phase. A significant interaction effect for stimulus cue × group (*p* = 0.010) was found. Sidak corrected post hoc comparisons using EMMs indicated a significantly higher shock expectancy rating for the CS+ compared to the CS− for all groups (all *p*s < 0.001) and higher shock expectancy ratings for the CS− in the SAD group compared to all other groups (all *p*s < 0.031).

A significant main effect of stimulus cue (*p* < 0.001), and a significant interaction effect for stimulus cue × group (*p* = 0.010) was found. For the stimulus cue × group interaction, Sidak corrected post hoc comparisons using EMMs indicated a significantly higher shock expectancy rating for the CS+ compared to the CS− for all groups (OCD: *t*(146) = −37.59, *p* < 0.001; SAD: *t*(146) = −33.93, *p* < 0.001; SP: *t*(146) = −37.46, *p* < 0.001; control: *t*(146) = −37.61, *p* < 0.001) and higher shock expectancy ratings for the CS− in the SAD group compared to all other groups (SAD versus OCD: *t*(289) = −2.95, *p* = 0.021; SAD versus SP: *t*(289) = −2.81, *p* = 0.031; SAD versus control: *t*(289) = −2.91, *p* = 0.023, see Figure 2).

##### 3.3.1.2 FPS

A significant main effect of stimulus cue (*p* < 0.001) was found with an overall increased response to the CS+ compared to the CS−. Importantly, the factor group and its interactions were not included in the reduced model, indicating no differences in FPS during acquisition between the categorical groups.

##### 3.3.1.3 LPP

**FIGURE 3:**
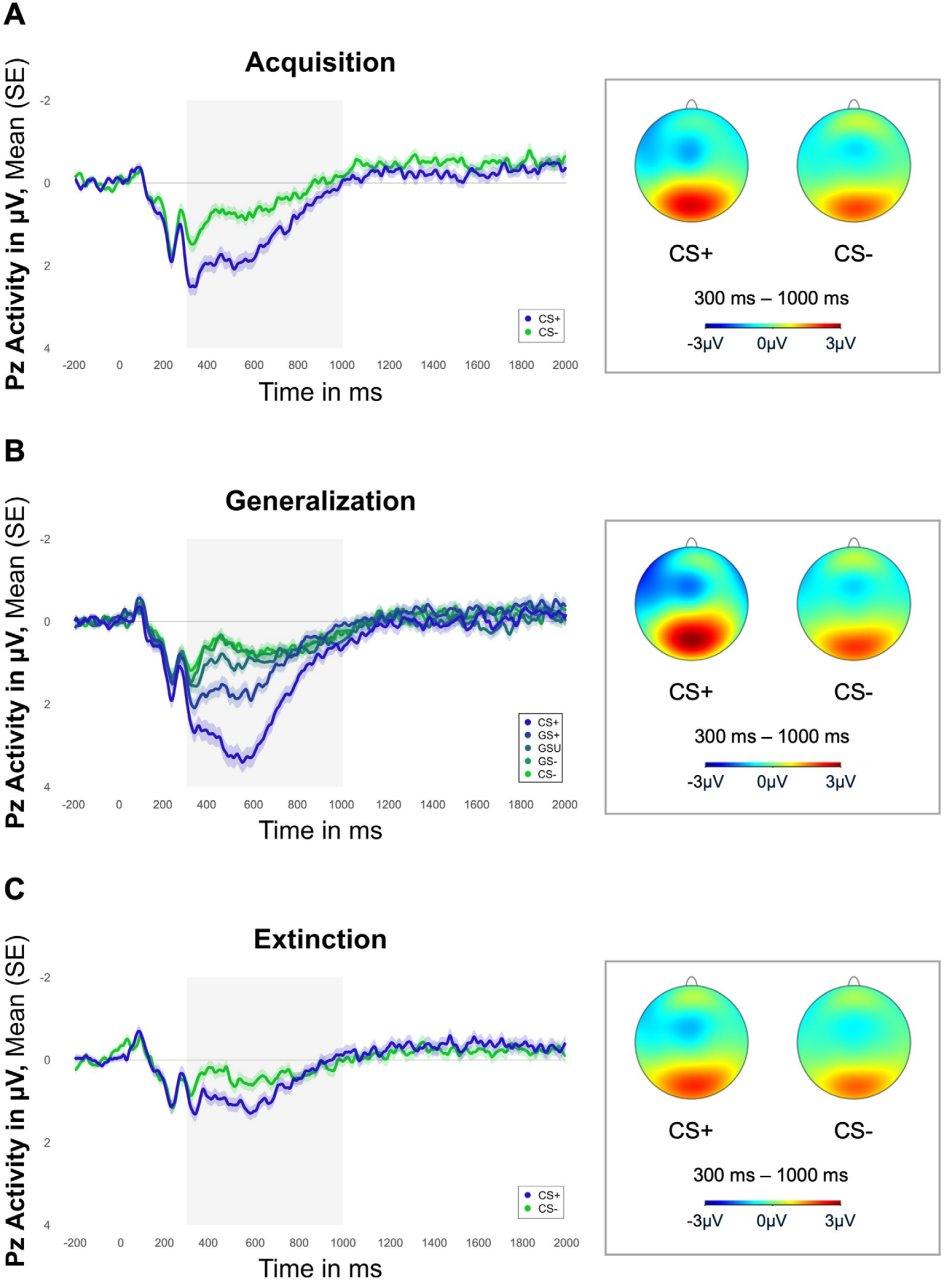
LPP activity across acquisition, generalization, and extinction. LPP, late positive potential. Depicted is the mean activity for the Pz electrode over all groups during (A) acquisition (significant mean effect of stimulus for the mean activity between 300 and 1000 ms after stimulus onset [*p* < 0.001]), (B) generalization (significant mean effect of stimulus for the mean activity between 300 and 1000 ms after stimulus onset [*p* < 0.001]), and (C) extinction (significant mean effect of stimulus for the mean activity between 300 and 1000 ms after stimulus onset [*p* = 0.020]).

A significant main effect of stimulus cue (*p* < 0.001), revealing an overall significantly higher response for the CS+ compared to the CS− (see Figure 3), was found. The main effect of group and its interaction with stimulus did not reach significance, indicating no categorical influence of the factor group.

#### 3.3.2 Dimensional Analysis

##### 3.3.2.1 Shock Expectancy

**FIGURE 4:**
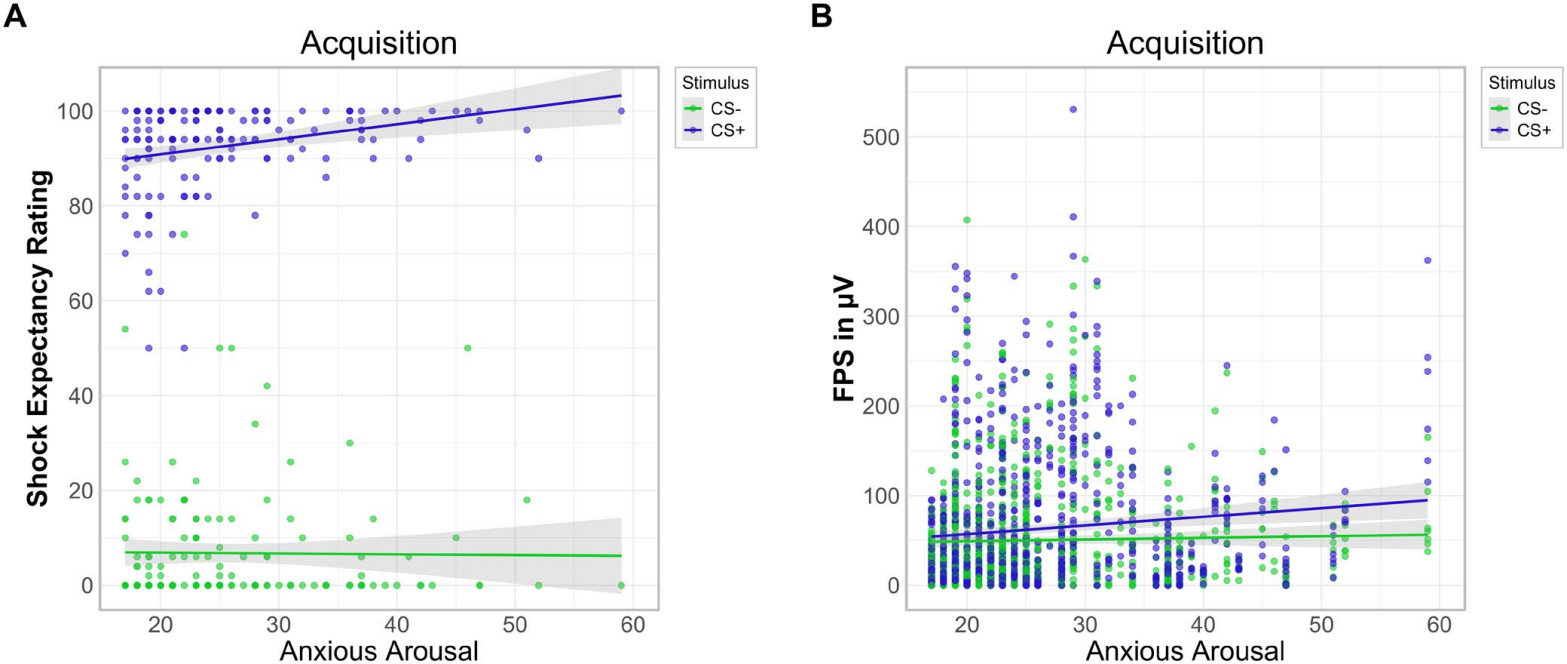
Influence of anxious arousal on shock expectancy ratings and fear‐potentiated startle during acquisition. FPS, fear‐potentiated startle. Anxious arousal measured with the sum score of the Anxious Arousal Subscale of the Mood and Anxiety Symptom Questionnaire (MASQ‐AA). Increased MASQ‐AA scores were associated with higher shock expectancy ratings for the CS+ (A) and with an elevated FPS response for the CS+ during acquisition (B).

A stimulus cue × MASQ‐AA interaction effect (*p* = 0.010) was found. Estimated marginal trends indicated that MASQ‐AA levels had a significant positive effect for the CS+ (*b* = 0.31, SE = 0.11, 95% CI: [0.10, 0.53]) but not for the CS− (*b* = −0.07, SE = 0.11, 95% CI: [−0.28, 0.15]). The contrast comparison confirmed that the slopes differed significantly (*t*(146) = −2.60, *p* = 0.010), indicating that increased MASQ‐AA scores were associated with higher shock expectancy ratings for the CS+ (see Figure 4).

##### 3.3.2.2 FPS

A significant interaction for stimulus cue × MASQ‐AA (*p* = 0.003) was found. Estimated marginal trends revealed that whereas the slopes were neither significant for CS− (*b* = 0.28, SE = 0.59, 95% CI: [−0.89, 1.45]) nor for the CS+ (*b* = 0.97, SE = 0.59, 95% CI: [−0.20, 2.14]), the slope for the CS+ was descriptively stronger and differed significantly in comparison to the CS− (*t*(1600) = −3.02, *p* = 0.003), indicating that higher MASQ‐AA scores were more strongly related to increased FPS for the CS+ (see Figure 4).

##### 3.3.2.3 LPP

A significant interaction of stimulus cue × group × BDI‐II (*p* = 0.047) was found, prompting further investigation using estimated marginal trends. Thus, for each stimulus cue × group combination, the slopes of BDI‐II levels on the LPP were estimated. While some descriptive differences in slopes emerged (e.g., a positive slope for the control group for the CS−), indicating an increase in response for higher BDI‐II levels (*b* = 0.06, SE = 0.06, 95% CI: [−0.05, 0.17]), pairwise contrasts of these slopes, however, revealed that none of the differences were statistically significant after Sidak correction (all *p*s > 0.800).

#### 3.3.3 Summary

Taken together, all groups showed the expected pattern of results in task effects across outcome measures. For the SAD group, an increased shock expectancy rating for the CS−; however, no significant differences for any of the other outcome measures or clinical groups could be found. Our hypotheses regarding different fear learning in SAD, SP, and OCD can therefore only be partially confirmed. Additionally, our hypotheses regarding anxious arousal and anxious apprehension can also be partially confirmed. While anxious arousal appears to be positively associated with shock expectancy rating and FPS responses for the CS+, anxious apprehension was not included in the reduced models, indicating it did not influence fear acquisition.

### 3.4 Fear Generalization

Model fit indices for the reduced models as well as all main and interaction effects for the generalization phase are displayed in Table 3. For the generalization phase, the following reduced models were computed:

$$\begin{array}{c} \text{Shock Expectancy}∼\mathrm{stimulus}\;\mathrm{cue}+\text{MASQ}‐\mathrm{AA}\\ \;+\text{stimulus}\;\mathrm{cue}\times \text{MASQ}‐\mathrm{AA}+\left(1\;|\;\mathrm{ID}\right) \end{array}$$

$$\mathrm{FPS}∼\text{stimulus}\;\mathrm{cue}+\left(1\;|\;\mathrm{ID}\right)$$

$$\begin{array}{c} \mathrm{LPP}∼\text{stimulus}\;\mathrm{cue}+\text{group}+\text{MASQ}‐\mathrm{AA}+\text{stimulus}\;\mathrm{cue}\\ \;\times \text{group}+\text{stimulus}\;\mathrm{cue}\times \text{MASQ}‐\mathrm{AA}+\text{group}\\ \;\times \text{MASQ}‐\mathrm{AA}+\text{stimulus}\;\mathrm{cue}\times \text{group}\times \text{MASQ}‐\mathrm{AA}\\ \;+\left(1\;|\;\mathrm{ID}\right) \end{array}$$

**TABLE 3:** Model fit indices and results of the Type III ANOVA with Satterthwaite's method for the generalization phase.

	$R_{c}^{2}$/$R_{m}^{2}$	ICC	RMSE	Σ	*F*	df	*p*	$R_{p}^{2}$	95% CI
Shock expectancy rating	0.795/0.733	0.231	16.57	17.78					
Stimulus cue					669.10	4, 596	**< 0.001**	0.752	0.727, 0.776
MASQ‐AA					3.06	1, 149	0.082	0.001	0.000, 0.011
Stimulus cue × MASQ‐AA					2.95	4, 596	**0.020**	0.013	0.004, 0.039
FPS^a^	0.646/0.012	0.642	38.76	39.48					
Stimulus cue					36.05	4, 3981	**< 0.001**	0.033	0.024, 0.047
LPP	0.429/0.147	0.330	1.21	1.34					
Stimulus cue					25.78	4, 564	**< 0.001**	0.026	0.011, 0.057
Group					0.05	3, 141	0.985	0.001	0.000, 0.015
MASQ‐AA					0.01	1, 141	0.908	0.003	0.000, 0.015
Stimulus cue × Group					0.92	12, 564	0.526	0.012	0.011, 0.050
Stimulus cue × MASQ‐AA					1.70	4, 564	0.150	0.006	0.002, 0.029
Group × MASQ‐AA					2.23	3, 141	0.087	0.005	0.001, 0.024
Stimulus cue × Group × MASQ‐AA					1.80	12, 564	**0.046**	0.023	0.017, 0.065

*Note:* Degrees of freedom are deviating for the outcome measures due to missing data. The full model included stimulus cue (i.e., CS+, GS+, GSU, GS−, and CS− for generalization), group (i.e., four levels: OCD, SAD, SP, and control), three mean‐centered continuous predictors (i.e., PSWQ, MASQ‐AA, BDI‐II) as well as their full interaction with stimulus cue and group. For each outcome measure, an individual reduced model was computed using a backward model selection procedure *p* < 0.05 are printed in bold.

Abbreviations: FPS = fear‐potentiated startle, LPP = late positive potential, MASQ‐AA = anxious arousal subscale of the Mood and Anxiety Symptom Questionnaire, $R_{c}^{2}$ = conditional *R* ^2^, $R_{m}^{2}$ = marginal *R* ^2^, $R_{p}^{2}$ = partial *R* ^2^.

^a^ Single trial data were used for the FPS.

#### 3.4.1 Categorical Analysis

##### 3.4.1.1 Shock Expectancy

A significant main effect of stimulus cue (*p* < 0.001) was found. Post hoc comparisons using EMMs (see Appendix H in the Supporting Information for stimulus cue comparisons and statistics) indicated not only an elevated response for the CS+ compared to all other stimuli but also for the GS+ (all *p*s < 0.001). The factor group and its interactions were not included in the reduced model, indicating no differences in shock expectancy ratings during generalization between the categorical groups.

##### 3.4.1.2 FPS

A significant main effect of stimulus cue (*p* < 0.001) was found. Sidak corrected post hoc comparisons using EMMs (see Appendix H in the Supporting Information for stimulus cue comparisons and statistics) revealed that FPS responses to the CS+ were significantly elevated compared to all other stimuli (all *p*s < 0.001). Additionally, the GS+ also elicited significantly higher FPS responses compared to the GSU (*p* < 0.001), the GS− (*p* = 0.005), and the CS− (*p* < 0.001). Note that group and its possible interactions were not included in the reduced model, indicating no influence of the factor group.

##### 3.4.1.3 LPP

A significant main effect of stimulus cue (*p* < 0.001) was found. Therefore, Sidak corrected post hoc comparisons of the main effect of stimulus using EMMs (see Appendix H in the Supporting Information for stimulus cue comparisons and statistics) were further investigated and revealed that the CS+ elevated a higher LPP response compared to all other stimuli (all *p*s < 0.001) as well as the GS+ compared to the GS‐ (*p* = 0.029) and CS− (*p* = 0.007; see Figure 3). Again, the main effect of group and its interaction with stimulus did not reach significance, indicating no categorical influence of the factor group.

#### 3.4.2 Dimensional Analysis

##### 3.4.2.1 Shock Expectancy

**FIGURE 5:**
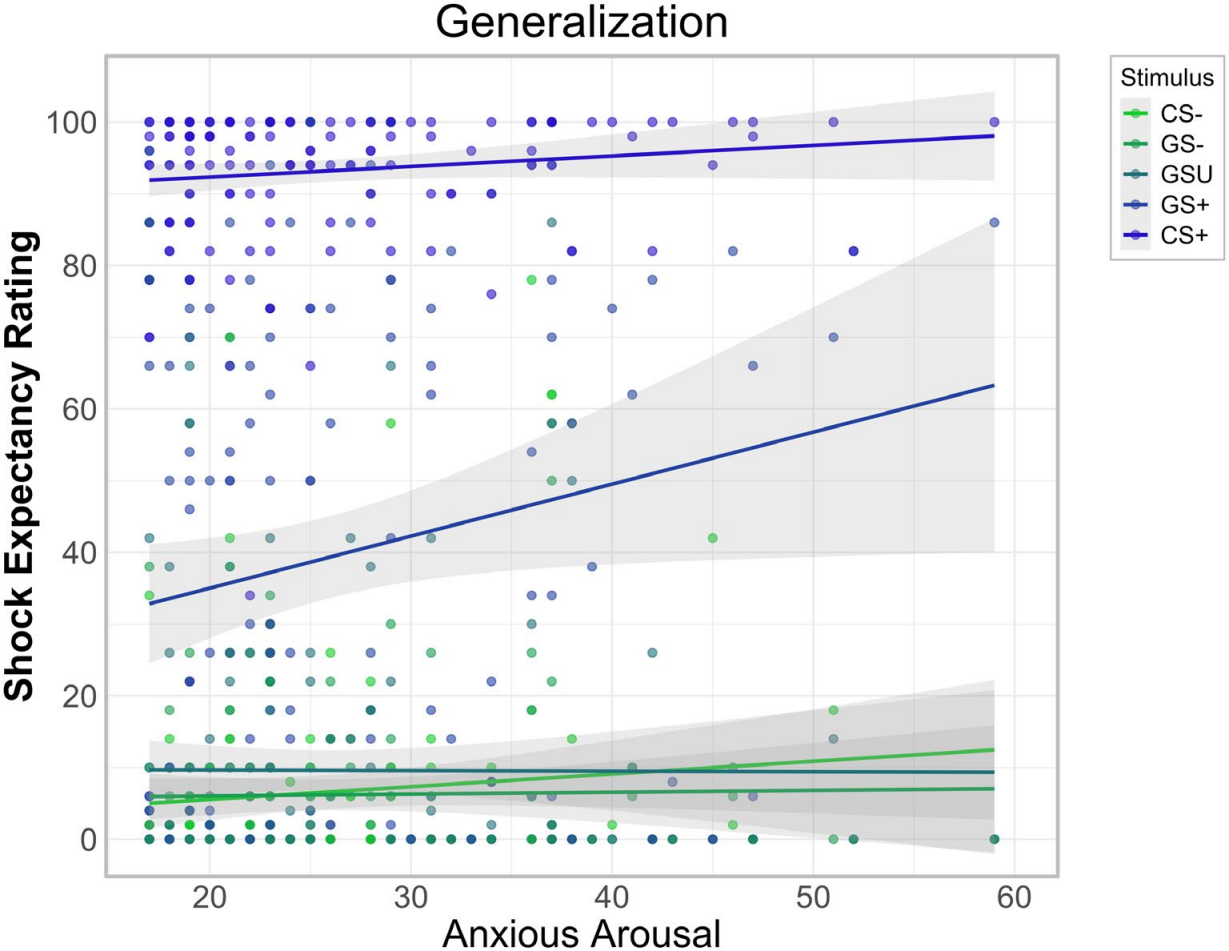
Influence of anxious arousal on shock expectancy ratings during generalization. Anxious arousal was measured with the sum score of the Anxious Arousal Subscale of the Mood and Anxiety Symptom Questionnaire (MASQ‐AA). Increased MASQ‐AA scores were associated with higher shock expectancy ratings for the GS+ during generalization.

A significant interaction for stimulus cue × MASQ‐AA (*p* = 0.020) was found. Further, estimated marginal trends revealed a significant positive association between MASQ‐AA levels and shock expectancy ratings for the GS+ (*b* = 0.73, SE = 0.20, 95% CI: [0.34, 1.11]), whereas all other stimuli showed non‐significant trends. Sidak corrected pairwise contrasts indicated that the slope for the GS+ was significantly steeper compared to the GS− (*t*(596) = −2.86, *p* = 0.043) and GSU (*t*(596) = −3.00, *p* = 0.028), indicating that higher MASQ‐AA scores were associated with higher shock expectancy ratings of the GS+ (see Figure 5). None of the other comparisons reached significance (all *p*s > 0.225).

##### 3.4.2.2 FPS

Symptom dimensions or their possible interactions were not included in the reduced model, indicating no influence of these variables on the FPS response during generalization.

##### 3.4.2.3 LPP

A significant interaction of stimulus cue × group × MASQ‐AA (*p* = 0.046) was found. Using estimated marginal trends, for each stimulus cue × group combination, the slope of MASQ‐AA levels on the LPP was estimated. There was a significantly negative association between the GS− in the SP group (*b* = −0.08, SE = 0.03, 95% CI: [−0.14, −0.01]), between the CS+ in the SAD group (*b* = −0.08, SE = 0.04, 95% CI: [−0.15, −0.01]) as well as a significantly positive association between the GS+ (*b* = 0.06, SE = 0.03, 95% CI: [0.00, 0.12]) and CS+ (*b* = 0.07, SE = 0.03, 95% CI: [0.01, 0.13]) in the OCD group and MASQ‐AA levels. However, for pairwise contrasts of these slopes, none of the differences were significant after Sidak correction (all *p*s > 0.304).

#### 3.4.3 Summary

**FIGURE 6:**
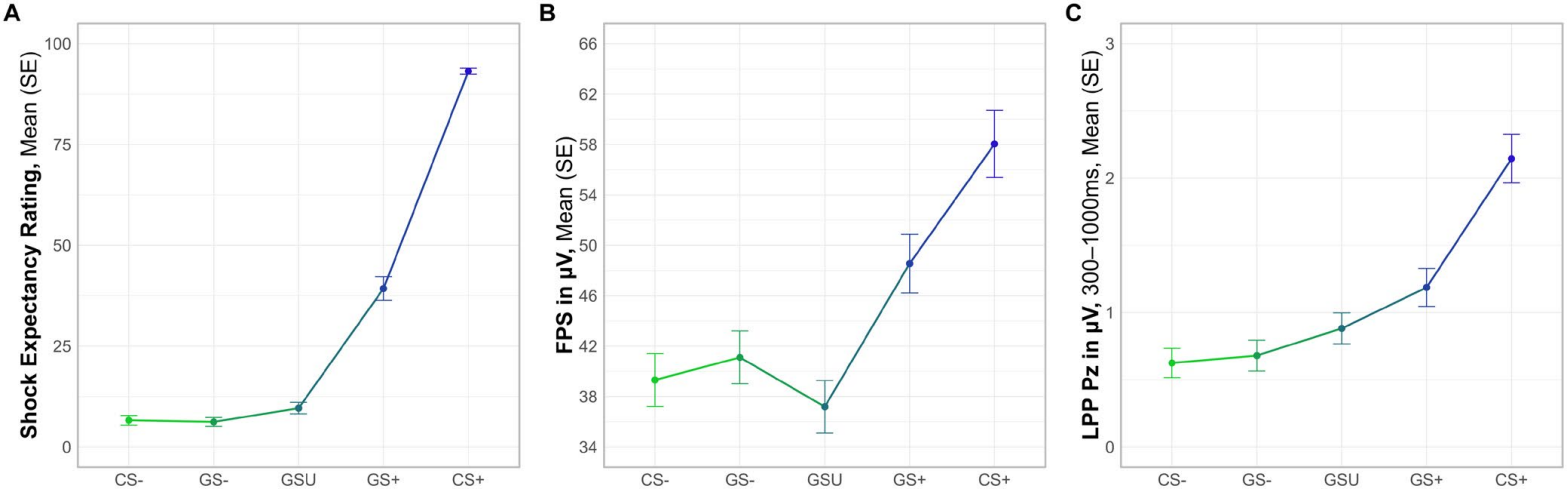
Fear generalization across the shock expectancy rating, fear‐potentiated startle, and late positive potential. FPS, fear‐potentiated startle; LPP, late positive potential.

Taken together, confirming our hypothesis, results suggest fear generalization for all groups in all outcome measures (i.e., shock expectancy rating, FPS, and LPP). We obtained a significant main effect of stimulus cue qualified by not only an elevated response for the CS+ compared to all other stimuli but also an elevated response for the GS+ compared to the GS− and CS− across all outcomes (see Figure 6; Supporting Information: Appendix H). Furthermore, mirroring the absence of group differences in the linear mixed‐effects models (i.e., either by the not included factor of group in the reduced models or by non‐significant group effect), comparison of the GI between all groups revealed no significant differences either (shock expectancy rating: *F*(3,147) = 0.25, *p* = 0.859, $\eta_{p}^{2}$ = 0.005; FPS: *F*(3, 143) = 0.66; *p* = 0.581, $\eta_{p}^{2}$ = 0.014; LPP: *F*(3, 145) = 0.35; *p* = 0.790, $\eta_{p}^{2}$ = 0.007). Thus, we did not confirm our hypotheses that patients with anxiety disorders or OCD would show increased fear generalization compared to controls. Regarding the dimensional hypotheses, we found a positive association between shock expectancy ratings for the GS+ and anxious arousal (i.e., higher MASQ‐AA levels were associated with higher shock expectancy ratings for the GS+) and no influence of anxious apprehension, which is partially in line with our hypotheses.

### 3.5 Fear Extinction

Model fit indices for the reduced models as well as all main and interaction effects for the extinction phase are displayed in Table 4. Backward model selection yielded the following models:

$$\text{Shock Expectancy}∼\mathrm{stimulus}\;\mathrm{cue}+\mathrm{BDI}‐\mathrm{II}+\left(1\;|\;\mathrm{ID}\right)$$

$$\mathrm{FPS}∼\text{stimulus}\;\mathrm{cue}+\left(1\;|\;\mathrm{ID}\right)$$

$$\mathrm{LPP}∼\text{stimulus}\;\mathrm{cue}+\left(1\;|\;\mathrm{ID}\right)$$

**TABLE 4:** Model fit indices and results of the Type III ANOVA with Satterthwaite's method for the extinction phase.

	$R_{c}^{2}$/$R_{m}^{2}$	ICC	RMSE	*σ*	*F*	df	*p*	$R_{p}^{2}$	95% CI
Shock expectancy rating	0.581/0.274	0.422	18.69	22.38					
Stimulus cue					154.37	1, 150	**< 0.001**	0.248	0.172, 0.330
BDI‐II					17.18	1, 149	**< 0.001**	0.083	0.034, 0.150
FPS^a^	0.636/0.008	0.633	39.99	40.68					
Stimulus cue					39.06	1, 1591	**< 0.001**	0.018	0.008, 0.034
LPP	0.308/0.013	0.299	1.09	1.25					
Stimulus cue					5.41	1, 148	**0.021**	0.013	0.000, 0.051

*Note:* Degrees of freedom are deviating for the outcome measures due to missing data. The full model included stimulus cue (i.e., CS+, and CS− for extinction), group (i.e., four levels: OCD, SAD, SP, control), three mean‐centered continuous predictors (i.e., PSWQ, MASQ‐AA, BDI‐II) as well as their full interaction with stimulus cue and group. For each outcome measure, an individual reduced model was computed using a backward model selection procedure *p* < 0.05 are printed in bold.

Abbreviations: BDI‐II = Beck Depression Inventory II, FPS = fear‐potentiated startle, LPP = late positive potential, $R_{c}^{2}$ = conditional *R* ^2^, $R_{m}^{2}$ = marginal *R* ^2^, $R_{p}^{2}$ = partial *R* ^2^, MASQ‐AA = anxious arousal subscale of the Mood and Anxiety Symptom Questionnaire.

^a^ Single trial data were used for the FPS.

#### 3.5.1 Categorical Analysis

##### 3.5.1.1 Shock Expectancy

Results showed a remaining significant stimulus cue effect with significantly increased values for CS+ compared to the CS−, suggesting no complete fear extinction learning (*p* < 0.001).

##### 3.5.1.2 FPS

There was a remaining significant stimulus cue effect. More precisely, there was a significantly higher response towards the CS+ compared to the CS−, indicating no complete fear extinction learning in the FPS response (*p* < 0.001).

##### 3.5.1.3 LPP

A significant stimulus cue effect remained (*p* = 0.021), with a higher LPP response for the CS+ compared to the CS− indicating no complete fear extinction learning in the LPP either (see Figure 3).

Importantly, the factor group and its interactions were not included in any of the reduced models, indicating no differences in shock expectancy, FPS, or LPP during extinction between the categorical groups.

#### 3.5.2 Dimensional Analysis

##### 3.5.2.1 Shock Expectancy

**FIGURE 7:**
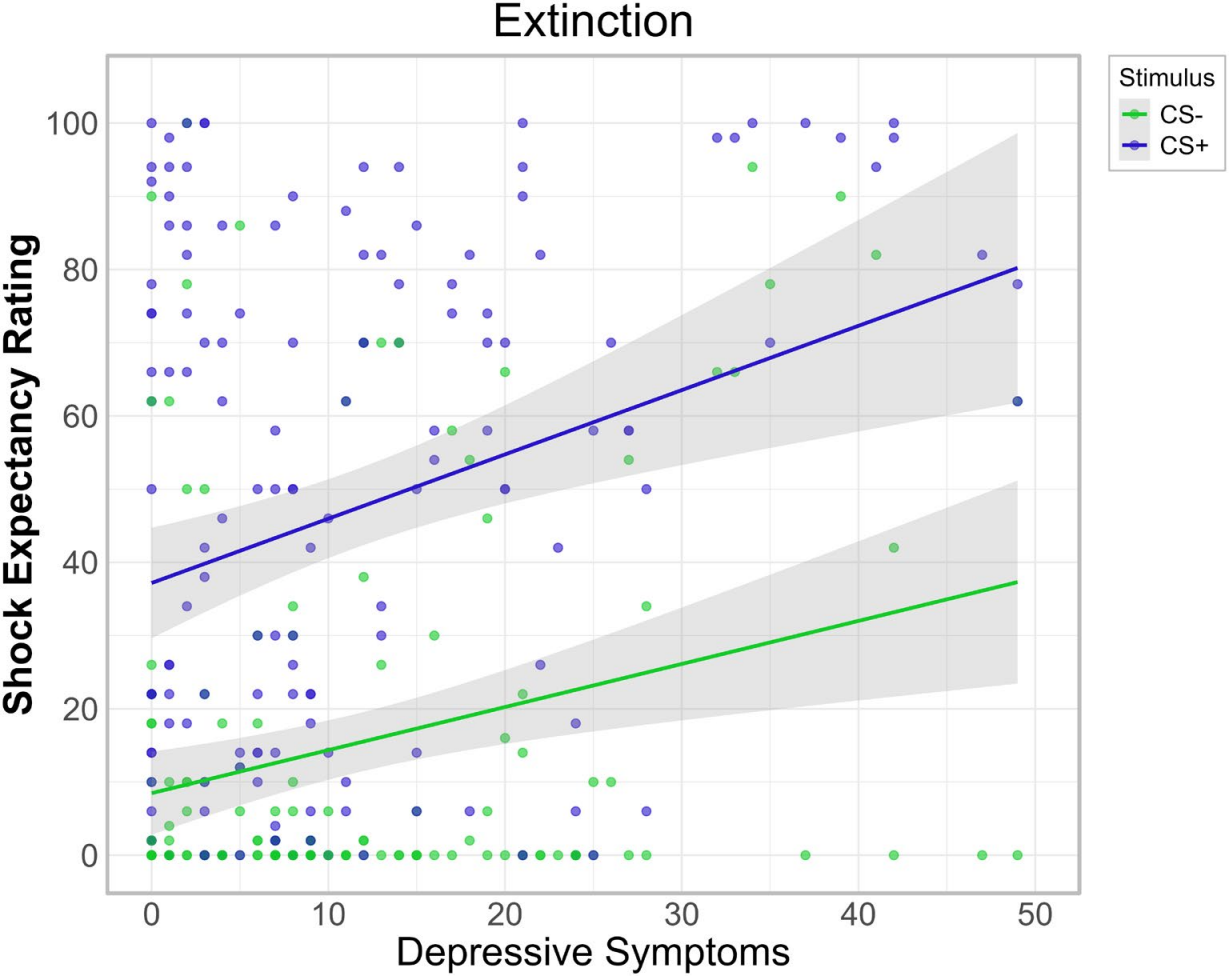
Influence of depressive symptoms on shock expectancy ratings during extinction. Depressive symptoms measured with the sum score of the Beck Depression Inventory II (BDI‐II). Increased BDI‐II levels were associated with higher shock expectancy ratings for the CS+ as well as the CS− during extinction.

There was a significant main effect of BDI‐II levels (*p* < 0.001). Estimated marginal trends revealed a significant positive association between BDI‐II levels and shock expectancy ratings (*b* = 0.73, SE = 0.18, 95% CI: [0.38, 1.08]) regardless of stimulus cue, that is, for both the CS+ and CS−, higher depressive symptoms led to increased shock expectancy during extinction (see Figure 7).

##### 3.5.2.2 FPS

Symptom dimensions or their possible interactions were not included in the reduced model, indicating no influence of these variables on the FPS response during extinction.

##### 3.5.2.3 LPP

Mirroring the FPS, symptom dimensions or their possible interactions were not included in the reduced model, indicating no influence of these variables on the LPP response during extinction.

#### 3.5.3 Summary

As our findings indicate that extinction learning seems to not have been fully completed, one linear mixed‐effects model for each outcome measure was carried out to test for changes over the course of phases. For an overview of model fit indices as well as all effects for the different outcome measures and post hoc comparisons, see Supporting Information: Appendix I.

##### 3.5.3.1 Shock Expectancy

The interaction between stimulus cue and phase was significant (*F*(3, 735) = 336.19, *p* < 0.001, $R_{p}^{2}$ = 0.177, 95% CI: [0.142, 0.218]). In all phases, except for the habituation (*p* = 0.409), the CS+ was rated significantly higher compared to the CS− (all *p*s < 0.001). Phase comparison revealed that for the CS+ the habituation rating was significantly lower compared to the ratings in all other phases (all *p*s < 0.001). The acquisition rating was not significantly different compared to the generalization rating (*p* > 0.999) but both were significantly higher compared to the extinction rating (all *p*s < 0.001). The CS− rating followed a different pattern. The habituation rating was significantly lower compared to the acquisition (*p* = 0.001) and generalization ratings (*p* < 0.001) but not the extinction rating (*p* > 0.999). The acquisition and generalization ratings did not differ significantly (*p* > 0.999) and both were significantly lower compared to the extinction rating (all *p*s < 0.002). Additionally, the model revealed a significant main effect of group (*F*(3, 147) = 3.39, *p* = 0.020, $R_{p}^{2}$ = 0.009, 95% CI: [0.003, 0.026]). Descriptively, the SAD and OCD group showed the highest ratings, regardless of stimulus cue and phase, however, Sidak corrected post hoc comparisons using EMMs revealed no significant differences (all *p*s > 0.125).

##### 3.5.3.2 FPS

There was a significant interaction between stimulus cue and phase (*F*(2, 4998) = 4.31, *p* = 0.014, $R_{p}^{2}$ < 0.001, 95% CI: [0.000, 0.003]). The CS+ was significantly higher compared to the CS− in all phases (all *p*s < 0.001). Phase comparison for the CS+ revealed that during acquisition the startle response was significantly higher compared to generalization (*p* = 0.035) and extinction (*p* < 0.001), and the generalization response was significantly higher compared to extinction (*p* < 0.001). For the CS−, the FPS response during acquisition was significantly higher compared to the generalization and extinction (all *p*s < 0.001), while generalization and extinction did not differ significantly (*p* = 0.995).

##### 3.5.3.3 LPP

Again, there was a significant interaction effect of stimulus cue and phase (*F*(2, 725) = 14.95, *p* < 0.001, $R_{p}^{2}$ = 0.013, 95% CI: [0.003, 0.033]). The LPP response for the CS+ was constantly higher compared to the CS− (all *p*s > 0.026). Phase comparison revealed that for the CS+, the LPP response during acquisition was significantly lower compared to the generalization phase (*p* < 0.001) and both were significantly higher compared to extinction (all *p*s < 0.001). There were no significant differences between the phases for the CS− (all *p*s > 0.276).

**FIGURE 8:**
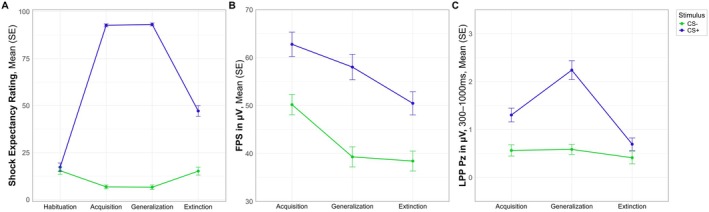
Fear and safety learning across all phases for the shock expectancy rating, fear‐potentiated startle, and late positive potential. FPS, fear‐potentiated startle; LPP, late positive potential.

In summary, the pattern of analyses suggests that there was, in fact, a decrease in responding towards the CS+ during the extinction phase compared to the acquisition and generalization phases in all outcome measures. This indicates that extinction learning took place but seems to not have been fully completed as the stimulus cue main effect remained significant throughout all outcome measures (for an overview, see Figure 8). We did not find any significant main effect of group nor significant interaction effects with group in either of the analyses. Therefore, we cannot confirm our hypotheses that extinction learning would be impaired in the anxiety and OCD groups compared to the control group. Anxious arousal and apprehension were not included in the reduced model, indicating that those symptom dimensions did not influence extinction learning; thus, we cannot confirm our dimensional hypotheses.

### 3.6 Results of the Preregistered Mixed‐Measures ANOVAs and Regression Models

The preregistered mixed‐measures ANOVAs for the categorical comparisons yielded results consistent with those obtained and reported from the linear mixed‐effects models (see Table 5 for an overview of the group and dimensional effects). For the dimensional analyses, however, we observed some differences. In the case of shock expectancy during generalization, MASQ‐AA levels showed a significant effect on responses to the GS+ in the linear mixed‐effect models, whereas the preregistered regression models indicated only a trend towards significance (*p* = 0.057). For FPS during acquisition, MASQ‐AA level effects reached significance in the linear mixed‐effects models but not in the regression models. Moreover, the regression models revealed an influence of BDI‐II levels on the FPS response to the CS– during generalization and extinction; however, BDI‐II levels were not included in the reduced linear mixed‐effect models reported here. Lastly, for the LPP, no differences emerged between the preregistered models and the linear mixed‐effect models. Results of the preregistered analysis are displayed in detail in Supporting Information: Appendix D.

**TABLE 5:** Overview of the group and symptom‐specific dimensional effects.

	Acquisition		Generalization					Extinction	
	CS+	CS−	CS+	GS+	GSU	GS‐	CS−	CS+	CS−
Group									
SAD		+ Exp							
SP									
OCD									
CON									
Dimensional									
PSWQ									
MASQ‐AA	+ Exp + FPS			+ Exp					
BDI‐II								+ Exp	+ Exp
Exploratory									
A+									
A−									
A+/D+								+ Exp Δ LPP	+ Exp Δ LPP
A+/D−									
A−/D−									

*Note:* The + indicates significantly increased responding and the Δ increased CS+/CS− differentiation for the specific outcome measurement (*p* < 0.05).

Abbreviations: A+ = participants with any current anxiety‐ or stress‐related disorder (i.e., anxiety disorders, obsessive‐compulsive disorder, post‐traumatic stress disorder; *n* = 117), A− = participants with no current anxiety‐ or stress‐related disorder (*n* = 39), A+/D+ = participants with a current anxiety‐ or stress related disorder and a current comorbid depression (i.e., current depressive episode or dysthymia; *n* = 24), A+/D− = participants with a current anxiety‐ or stress‐related disorder without a current comorbid depression (*n* = 93), A−/D− = participants without a current anxiety‐ or stress‐related disorder or comorbid depression (*n* = 39), BDI‐II = Beck Depression Inventory II, CON = control group, Exp = shock expectancy rating, FPS = fear‐potentiated startle, MASQ‐AA = anxious arousal subscale of the Mood and Anxiety Symptom Questionnaire, OCD = obsessive‐compulsive disorder group, PSWQ = Penn State Worry Questionnaire, SAD = social anxiety disorder group, SP = specific phobia group.

### 3.7 Exploratory Analyses

All results of the exploratory analyses can be found in detail in the Supporting Information: (Appendix I).

#### 3.7.1 Bayesian Analyses

Bayesian analyses, which quantify evidence in favor of the null hypothesis, did not reveal any group differences for the primary outcomes (all P(M|data) < 0.037, all BF_10_ < 0.067). This does strengthen our reported null effects for most of the group comparisons. The significant stimulus cue × group interaction observed for shock expectancy ratings during acquisition should be interpreted carefully as the model including the stimulus cue × group interaction (P(M|data) = 0.419, BF_10_ = 0.915) does not improve model fit compared to the model only including stimulus cue (P(M|data) = 0.458, BF_10_ = 1.000).

#### 3.7.2 Influence of Current Anxiety‐ or Stress‐Related Disorders

To assess the effect of clinical status regardless of disorder category, we compared individuals with any current anxiety‐ or stress‐related disorder (i.e., anxiety disorders, OCD, PTSD; *n* = 117) to those without such a diagnosis (*n* = 39). This comparison did not reveal any group differences for any of the phases or outcomes (all *p*s > 0.113; see Appendix I in the Supporting Information for model fit indices as well as effects for the different outcome measures across all phases).

#### 3.7.3 Influence of Comorbid Depression

**FIGURE 9:**
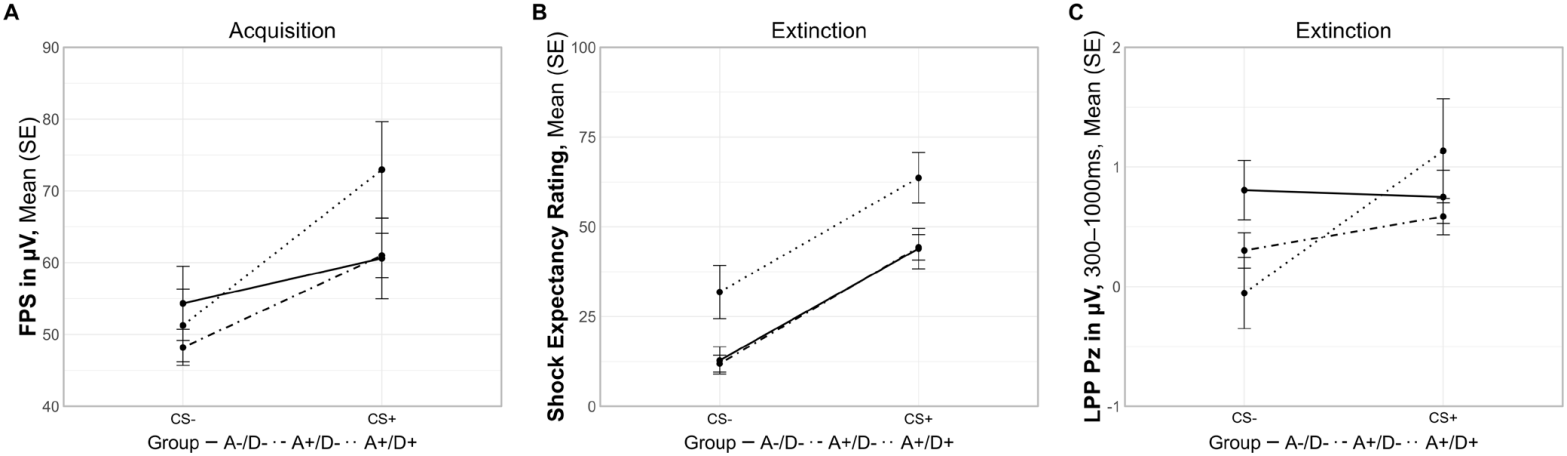
Differences of individuals with a current anxiety‐ or stress‐related disorder and current comorbid depression compared to participants without. A−/D− = No current anxiety‐ or stress‐related disorder, no current depression; A+/D− = current anxiety‐ or stress‐related disorder, no current comorbid depression; A+/D+ = current anxiety‐ or stress‐related disorder, current comorbid depression. Anxiety‐ or stress‐related disorders included a current diagnosis of any anxiety disorder, OCD, or PTSD. Depression included a current depressive episode or dysthymia. There was a significant stimulus cue × group interaction for the FPS during the acquisition phase (*p* = 0.047) pointing to a larger effect for the CS+/CS− differentiation for the A+/D+ group (A), a significant effect of group for the shock expectancy rating during extinction (*p* = 0.004), with significantly higher shock expectancy ratings for the A+/D+ group in comparison to the other groups (all *p*s < 0.013; B), and a significant stimulus cue × group interaction for the LPP during extinction (*p* = 0.025), with the CS+/CS− differentiation in LPP activity being significant for individuals with a comorbid depression (*p* = 0.001), but not for individuals with an anxiety‐ or stress‐related disorder without a comorbid depression and those without any disorder (all *p*s > 0.128; C).

Additionally, based on the findings related to dimensional BDI‐II effects, we compared individuals with a current anxiety‐ or stress related disorder with a current comorbid depression (i.e., current depressive episode or dysthymia; *n* = 24; A+/D+), to individuals without a current comorbid depression (*n* = 93; A+/D−), and individuals without any anxiety‐ or stress‐related disorder (*n* = 39; A−/D−). The results (see Supporting Information: Appendix I in the supplement for model fit indices as well as effects for the different outcome measures across all phases) revealed a significant stimulus cue × group interaction for the FPS during the acquisition phase (*F*(1, 1598.31) = 3.07, *p* = 0.047, partial *R* ^2^ = 0.003, 95% CI: [0.000, 0.011]). Post hoc comparisons using EMMs indicated a consistent difference between the CS+ and the CS− with a significantly higher response towards the CS+ (acquisition: *t*(1599) = −2.16, *p* = 0.031; generalization: *t*(1598) = −4.70, *p* < 0.001; extinction: *t*(1599) = −4.77, *p* < 0.001), and group differences were nonsignificant after Sidak correction (all *p*s > 0.764). However, for individuals without any disorder (A−/D−), the estimated difference was −7.84 (SE = 3.97, *p* = 0.049), for individuals with an anxiety‐ or stress‐related disorder but without a comorbid depression (A+/D−) it was −11.72 (SE = 2.52, *p* < 0.001), and for individuals with a comorbid depression (A+/D+) it was −24.39 (SE = 5.02, *p* < 0.001), pointing to a larger effect in individuals with mental disorders and especially comorbid depression (see Figure 9). Further group differences were found for the extinction phase: There was a significant effect of group for the shock expectancy rating (*F*(2, 148) = 5.82, *p* = 0.004, partial *R* ^2^ = 0.030, 95% CI: [0.006, 0.083]). Sidak corrected post hoc comparisons using EMMs revealed that A+/D+ individuals compared to A+/D− individuals rated the shock expectancy significantly higher regardless of stimulus cue (*t*(148) = −3.32, *p* = 0.003) as well as A−/D− individuals (*t*(148) = −2.89, *p* = 0.013; see Figure 8). Lastly, there was a significant stimulus cue × group interaction for the LPP during extinction (*F*(2, 146) = 3.79, *p* = 0.025, partial *R* ^2^ = 0.018, 95% CI: [0.002, 0.064]). Post hoc pairwise comparisons revealed that the differentiation between the CS+ and CS− in LPP activity was significant for A+/D+ (*t*(146) = −3.29, *p* = 0.001), but not for A+/D− individuals (*t*(146) = −1.53, *p* = 0.128) or those without any disorder (*t*(146) = 0.20, *p* = 0.842; see Figure 8). Together, this aligns with the pattern of the results in the dimensional analyses. Further, it reinforces the role of depressive symptomatology in supporting that depressive symptoms were associated with impaired safety learning on a self‐report level.

## 4 Discussion

The current study examined fear learning in a large transdiagnostic sample of participants who were recruited based on their primary diagnosis of SAD, SP, or OCD, as well as control participants using multiple outcomes, that is, shock expectancy rating, FPS, and LPP data. To study fear learning, a differential fear conditioning paradigm was employed, including acquisition, generalization, and extinction. Our present results indicate that: (1) all participants acquired fear for the CS+ compared to the CS−; (2) all participants generalized fear to the GS+; (3) during extinction learning there was a significant decrease in fear for the CS+ compared to acquisition and generalization; however, significantly higher reactions towards the CS+ compared to the CS− remained, indicating only partial extinction learning; (4) contrary to our expectations, patients with SAD, SP, and OCD did not show altered fear learning processes compared to controls, with one exception: During acquisition, SAD patients rated the shock expectancy for the CS− significantly higher compared to all other groups; (5) increased anxious arousal was associated with higher CS+ shock expectancy ratings and FPS response during acquisition and higher GS+ shock expectancy ratings during generalization; (6) higher depressive symptoms were associated with higher CS+ and CS− shock expectancy during extinction; and (7) exploratory analyses revealed that participants with an anxiety‐ or stress‐related disorder (i.e., anxiety disorder, OCD, PTSD) and a comorbid depression (i.e., current depressive episode or dysthymia) compared to participants without a comorbid depression and healthy participants showed greater CS+/CS− differentiation in their FPS response during acquisition as well as a higher shock expectancy ratings and a significant CS+/CS− differentiation in LPP activity during extinction.

Regarding the basic effect of fear conditioning on a behavioral and psychophysiological level, that is, the acquisition, generalization and extinction in shock expectancy ratings and FPS, the findings of our paradigm are in line with previous studies (e.g., Lissek et al. 2008; Lonsdorf et al. 2017). Comparatively fewer researchers have used EEG to study the neural correlates of fear learning. Here, this study makes a particularly important contribution.

Overall, it has been reported that during fear acquisition, LPP responses to the CS+ are significantly enhanced compared to the CS−. This effect has been found for different types of stimuli such as faces (Bruchmann et al. 2021; Ferreira De Sá et al. 2019; Panitz et al. 2015; Sperl et al. 2021) and geometrical forms (Bacigalupo and Luck 2017; Dou et al. 2023; Paiva et al. 2020; Seligowski et al. 2018) as well as different aversive US types (i.e., electrotactile stimulation (Dou et al. 2023; Ferreira De Sá et al. 2019; Sperl et al. 2021), sound (Bacigalupo and Luck 2017; Bruchmann et al. 2021; Paiva et al. 2020; Panitz et al. 2015) or airblast (Seligowski et al. 2018)). Thus, for the LPP during fear acquisition, our findings strengthen and extend previous research indicating that successful fear learning is accompanied by increased attention and emotional processing of the CS+ as indicated by larger LPP amplitudes.

For fear generalization, the present data suggest continued fear learning to the CS+ and fear generalization to the GS+ on a neural level. To our knowledge, only one study investigated generalization and the LPP in a differential fear conditioning paradigm. Klein et al. (2024) focused on the comparison between anxious and non‐anxious youth in a fear generalization and extinction recall paradigm. Anxious compared to non‐anxious youth showed a significantly larger LPP for the CS+ as well as the stimulus most similar to the CS+. We did not observe group differences in our LPP data during generalization. Here, developmental differences could play a role, as neural responses to fear generalization may vary between anxious youth and adults. Two other studies investigated the LPP in an observational learning (Dou et al. 2023) and backward masking fear conditioning paradigm (Mei et al. 2024). No fear generalization was reported for the LPP in these studies (Dou et al. 2023; Mei et al. 2024), which is in contrast to our results that indicate higher attentional and emotional processing for the stimulus most similar to the CS+. These discrepancies may be explained by variations in task design, as observational learning as well as backward masking fear conditioning could influence LPP responses in a different way than differential fear conditioning, thus complicating comparisons. Further, we cannot rule out that differences in the similarity of the GS compared to the CS+ and CS− might additionally influence LPP responses, potentially accounting for the gradual increase in the LPP response observed in our data but not in previous research (Dou et al. 2023; Mei et al. 2024). Our data show that the LPP gradually increases with increasing proximity to the conditioned stimulus (CS+), highlighting that more neural resources and attention are allocated to the processing of stimuli that are perceptually similar to the CS+. This may serve as the basis for enhanced and faster reactions to threat‐associated stimuli. However, more research focusing on fear generalization using the LPP as an outcome measurement is needed to better understand the underlying mechanisms.

After extinction learning, the response towards the CS+ compared to the CS− remained significantly increased. However, the significant decrease in the response towards CS+ during the extinction phase, compared to both acquisition and generalization, indicates that extinction learning occurred but was not fully completed. Here, our findings are partially in line with previous research on LPP and extinction learning that reported complete extinction (Bacigalupo and Luck 2017; Ferreira De Sá et al. 2019; Panitz et al. 2015). However, our findings underscore how persistent and stable learned threat associations can be, indicating that longer extinction periods may be necessary to successfully complete the process. Regarding the outcome measures, we report the smallest effect size of the main stimulus effect during extinction for the LPP compared to shock expectancy and FPS data. This suggests that, in comparison to verbal self‐report and the startle reflex, the attentional allocation and neural processing of the stimuli were less differentiated between the CS+ and CS−, indicating that extinction was more pronounced here. Thus, there might be different processes and dynamics underlying safety learning at a neural level compared to the behavioral level (Gazendam and Kindt 2012; Haaker et al. 2013, 2014), or indicating that conscious reports may adapt more slowly than initial attentional allocation.

Our study is one of the first to assess EEG data in fear conditioning in a large transdiagnostic sample of patients with SAD, SP, OCD, and unaffected controls. While many studies examine fear conditioning in anxiety, few include multiple disorder groups, which makes it difficult to identify distinct and shared effects. However, aside from a significant effect in the rating data (i.e., higher shock expectancy ratings for individuals with SAD to the CS− during acquisition), none of the outcomes related to the participants' clinical status and no group differences or interactions with group were observed. This is in line with a recently conducted study that investigated differences in fear acquisition and extinction learning between patients with anxiety disorders, depression, and controls who did not report any differences in safety or extinction learning between the groups using subjective ratings and SCR (Adolph et al. 2023). On the other hand, these findings are at odds with the above described meta‐analytical evidence that reported heightened fear responses to conditioned safety cues during fear acquisition and reduced fear extinction in patients with anxiety disorders and OCD (Duits et al. 2015; Kausche et al. 2025). However, the observed increase in expectancy ratings for the CS− in individuals with SAD is consistent with the existing literature (e.g., Ahrens et al. 2015; Fyer et al. 2020; Hermann et al. 2002). Notably, the most recent meta‐analysis (Kausche et al. 2025) demonstrates that group differences are most robustly observed in self‐report measures, which is also reflected in our data. Nonetheless, our studies and others suggest that changes in fear conditioning in anxiety‐ and stress‐related disorders may be smaller and less robust than often assumed.

There are several possible explanations for why we did not find other differences in fear learning between patients with SAD, SP, OCD, and controls. Firstly, the task itself may have left little room for variance as participants were instructed to learn what circle was paired with the aversive stimulation and it might have been too easy with the reinforcement rate too high, leading to ceiling effects and limited variability, therefore, diminishing the possibility to find group differences. Secondly, it is questionable whether the used stimuli were relevant enough, in the sense that geometrical forms do not play an important role neither in our everyday lives nor for the examined disorders (e.g., compared to faces or spiders; e.g., Åhs et al. 2018; Ney et al. 2022). Here, future studies could increase ecological validity by using more symptom‐specific stimuli. Thirdly, some findings from other studies indicate differences between anxious and non‐anxious individuals only in delayed and not immediate extinction (e.g., Klein et al. 2024). It might thus be possible that differences between participants with and without anxiety‐ and stress‐related disorders do not arise from differences in extinction learning but rather from differences in memory consolidation (Beckers and Kindt 2017) that become apparent during delayed extinction or extinction recall tests. In the present paradigm, only immediate extinction was investigated which leaves no room for memory consolidation (Lonsdorf et al. 2017). Here, future studies are needed to investigate the differences of immediate and delayed extinction processes in anxiety‐ and stress‐related disorders. Lastly, previously reported differences in fear conditioning between individuals with anxiety and those without might not be as large as expected or cannot be transferred to all anxiety‐ and stress‐related disorders (Cooper et al. 2024). This idea is supported by the methodological limitations of the meta‐analytical evidence. For example, Duits et al. (2015) have also included older studies with no clear diagnosis criteria and Kausche et al. (2025) report considerable symptomatic heterogeneity and symptom overlap between different anxiety‐ and stress‐related disorders which limits the generalizability of differences in fear conditioning across disorders.

Due to the substantial symptom overlap among different anxiety disorders as well as comorbidities (e.g., Cooper et al. 2024; Craske et al. 2009; Goldstein‐Piekarski et al. 2016; Krueger 1999; Saha et al. 2021), we analyzed the data with a focus on the dimensional effects of transdiagnostic symptoms, specifically targeting core symptom dimensions relevant to anxiety‐ and stress‐related disorders: anxious apprehension, anxious arousal, and depressive symptoms. This dimensional analysis did not find associations between anxiety dimensions or depressive symptoms and neural responses that sustained after correction for multiple comparisons. It did, however, suggest that increased anxious arousal was associated with higher shock expectancy ratings and FPS responses for the CS+ during acquisition, and higher shock expectancy ratings of the GS+ during generalization. Given that hyperarousal is a key symptom of PTSD (Yang et al. 2017) and a central component of the anxious arousal dimension (Härpfer et al. 2021), our finding of an association between higher anxious arousal and elevated shock expectancy ratings for the CS+ during acquisition aligns with previous evidence of altered fear learning in PTSD (Duits et al. 2015; Kausche et al. 2025) as well as altered fear generalization in PTSD (Cooper et al. 2022). Thus, heightened arousal and a state of readiness to respond to threatening situations translates into an increased expectation of shock in response to the CS+. Furthermore, elevated arousal states seem to amplify threat perception and the generalization of fear responses (as seen in shock expectancy rating results), thereby strengthening learned associations and contributing to maladaptive fear responses across disorders. Further, increased depressive symptoms were linked to higher shock expectancy ratings of the CS+ and the CS− during extinction. This is in line with research supporting that depressive symptoms predict problems in extinction learning (Bylsma et al. 2008) and aligns with the meta‐analysis by Kausche et al. (2025) that also reported a modulation of extinction by depressive symptoms. Additionally, our exploratory analyses dividing the sample by current comorbid depression further strengthens the modulating role of depression. It revealed a significant group effect for shock expectancy ratings during extinction, supporting the association between increased shock expectancy for both CS+ and CS− during extinction in individuals with a comorbid depression, indicating disrupted extinction processes. An overall blunting of the LPP was not significant for the subgroup with comorbid depression in any of the phases. This finding might be considered unexpected, as it contrasts with previous research reporting reduced LPP amplitudes in depression for both positive and negative stimuli (Hajcak and Foti 2020; Weinberg et al. 2016). However, those studies did not employ fear conditioning paradigms, which limits the comparability. The impaired extinction in the subgroup with comorbid depression is further supported by neural data in the LPP, where only those participants with an anxiety‐ and stress‐related disorder and a comorbid depression still differentiated between the CS+ and the CS− during extinction, whereas the other groups did not. The impaired extinction learning observed in our study may reflect processes related to learned helplessness, and in line with prior evidence of impaired extinction in depression, it seems reasonable to assume that this may not only be visible in subjective ratings but also in the LPP. Nevertheless, the interplay between anxiety‐ and stress‐related disorders with and without comorbid depression, and how these are reflected in the LPP, remains insufficiently understood and should be addressed in future research. However, whereas these findings align well and support the idea of the influence of depressive symptoms on altered extinction learning processes, the differences in group sizes for these exploratory analyses need to be considered when interpreting our findings. Taken together, these findings suggest that depressive symptoms may hinder the extinction of learned fear responses, leading to an increased expectation of threat also evident on a neural level, even when no danger is present. Elevated expectancy ratings in depressed individuals may indicate a greater attentional bias towards threatening cues, which could exacerbate anxiety and fear responses (Hallion and Ruscio 2011). Thus, it is essential to consider the interaction between depression and anxiety‐ and stress‐related disorders when evaluating fear and extinction processes. Furthermore, addressing disrupted extinction learning in individuals with comorbid depression could be key to improving interventions and therapy outcomes. On the one hand, the presence of depressive symptoms may indicate that exposure therapy requires more sessions or an extended duration to be effective, thereby contributing to individualized treatment planning. On the other hand, specific interventions—such as integrating mood‐regulating strategies or combining exposure with cognitive techniques—to address depressive symptoms, cognitive biases, or distortions, may be used to enhance treatment efficacy. A multimodal approach that considers both anxiety and depressive symptoms could significantly improve treatment outcomes for individuals with comorbid conditions.

Some limitations need to be considered when interpreting the present findings. It has been reported that the number of extinction trials has an effect on extinction learning (Harris and Andrew 2017). As the difference between the CS+ and CS− during extinction remained and erasure mechanisms seem to become more prominent as the extinction training proceeds (An et al. 2017), it is possible, that the extinction phase in our paradigm was not long enough. Another influencing factor of the incomplete extinction process may have been that the startle probe was presented consistently during extinction. The startle probe was not associated with a specific stimulus, but still perceived as aversive. This could have interfered with the extinction process since it might have created a general aversive context that diminished stimuli specific threats. However, this interpretation is not based on existing literature and future studies should examine this potential impact. Additionally, we only collected data on current mental disorders and not on mental disorders in the past. Furthermore, the control group consisted of a community sample and mental disorders other than anxiety‐ and stress related disorders were not an exclusion criterion within this group. This way, our sample was more representative, on the other hand, however, we cannot rule out, that not knowing about past mental disorders as well as not having exclusively healthy controls might have led to smaller group effects in our study and may complicate interpretation of group differences. However, comparing individuals with an anxiety‐ or stress‐related disorder to those individuals without revealed no significant differences either. Future research should include comprehensive assessments of participants' mental health histories, including past mental disorders, to better account for their potential influence on the observed effects. Further, sensitivity analyses indicated reduced statistical power for some smaller interaction effects including dimensional variables, particularly the three‐way interaction of the LPP, which fell below the commonly recommended 80% threshold (Lakens 2022). While these effects were not interpreted further due to non‐significant post hoc comparisons, it is noteworthy that for the interaction effects including dimensional variables of the shock expectancy rating during acquisition and generalization, power was slightly below 80% (i.e., 74.3% and 77.8%, respectively). Thus, these effects should be interpreted with this consideration in mind. Lastly, all the participants completed the fear conditioning paradigm as the last of multiple tasks in our lab. Due to the length of the laboratory assessment, it cannot be ruled out that concentration was reduced towards the end of the assessment, however, we did not directly assess this. Further, prior to the fear conditioning paradigm, a 12‐min‐long no‐threat, predictable threat, unpredictable threat task was conducted, which also used electrotactile stimulation. Therefore, the electrotactile stimulation was not novel for our participants. We asked participants whether they still perceived the electrotactile stimulation as unpleasant before starting the fear conditioning paradigm and re‐calibrated when necessary. Despite showing strong task effects in the current sample, evidence suggest that habituation of electrotactile stimulation takes place over many trials (Sperl et al. 2016) and, thus, might have impacted the fear conditioning in our paradigm and might have also altered extinction learning as participants were exposed to the electrotactile stimulation longer.

In conclusion, the fear conditioning paradigm used in this study effectively enabled fear learning, generalization, and initial extinction learning at neural and psychophysiological level as well as in shock expectancy ratings. Consistent with the overall pattern, all three measures demonstrated similar task effects, that is, increased responding towards the CS+ during fear acquisition, increased responding towards the CS+ and GS+ during fear generalization, as well as a decreasing response towards the CS+ during fear extinction, suggesting a common but incomplete extinction process. Some differences regarding group and symptom‐specific dimensional analyses emerged between outcome measures. There were no differences observed in fear acquisition, generalization, or extinction between patients with OCD, SP, and controls. Only patients with SAD, as frequently reported, exhibited heightened responses in shock expectancy ratings to the safety stimuli (CS−) during acquisition. This is in line with recent meta‐analytical findings that report the highest group differences between anxiety patients and controls in subjective ratings (Kausche et al. 2025). To complement the categorical analyses—whose interpretability is limited by overlapping symptoms, common comorbidities, and smaller group sizes—we also performed dimensional analyses across the entire sample. These analyses identified significant effects of anxious arousal and depressive symptoms on fear learning processes, highlighting their relevance beyond diagnostic categories. Increased anxious arousal was associated with an increased shock expectancy of the CS+ and amplified FPS responses for the CS+ during acquisition supporting the notion that heightened arousal enhances threat expectation and defensive responses in fear learning processes. Increased depressive symptoms were associated with higher shock expectancy ratings for both the CS+ and CS− during extinction, suggesting that depressive traits may impair the ability to differentiate safety signals from threat cues, thereby influencing fear regulation processes. No dimensional influences were found for LPP data. The partial alignment of these subjective and physiological indices emphasize that different measures may reveal complementary aspects of fear learning processes. Future studies should investigate a range of outcome measures to capture this complexity. Together, this highlights the potential and underscores the need of further research on transdiagnostic influences on fear learning processes. It also highlights that the frequently co‐occurring comorbid depressive symptoms can impact fear extinction, and therefore, should be considered in the treatment of anxiety disorders to enhance therapeutic outcomes.

## Author Contributions

**Kim M. Sobania:** data curation, formal analysis, investigation, methodology, project administration, validation, writing – original draft, writing – review and editing, visualization. **Kai Härpfer:** conceptualization, investigation, validation, writing – review and editing. **Hannes P. Carsten:** conceptualization, validation, investigation, writing – review and editing. **Tania M. Lincoln:** writing – review and editing. **Franziska M. Kausche:** conceptualization, data curation, investigation, methodology, project administration, software, validation, writing – original draft, writing – review and editing, supervision. **Anja Riesel:** conceptualization, funding acquisition, methodology, project administration, resources, validation, writing – original draft, writing – review and editing, supervision.

## Funding

This research was supported by the German Research Foundation (Deutsche Forschungsgemeinschaft—DFG, grants RI‐2853/2‐1 awarded to A. Riesel and GRK 2753/1 project number 449640848).

## Conflicts of Interest

The authors declare no conflicts of interest.

## Supporting information


**Data S1:** psyp70277‐sup‐0001‐DataS1.docx.

## Data Availability

The data that support the findings of this study are available on request from the corresponding author. The data are not publicly available due to privacy or ethical restrictions.

## Endnotes

^1^
Preregistered here: osf.io/cfqm9. We deviated from the preregistration regarding the analysis of the skin conductance response, the reinforcement rate during generalization, preprocessing of the fear‐potentiated startle, one hypothesis, the use of linear mixed‐effect models, and additional exploratory analyses. Reasons for deviations from preregistration are listed in Supporting Information: Appendix A.
