# Supplementary material for: Are Fear Learning Processes Altered in Obsessive‐Compulsive Disorder, Social Anxiety, and Specific Phobia? Insights From the Late Positive Potential, Fear‐Potentiated Startle, and Ratings
Source: Psychophysiology. 2026 Mar 23;63(3):e70277. doi: 10.1111/psyp.70277 (PMC13009327; doi:10.1111/psyp.70277)
Supplement: Supplementary file 1 — Data S1: psyp70277‐sup‐0001‐DataS1.docx. [file PSYP-63-e70277-s001.docx]

**Appendix A**

Reasons for Deviations from Preregistration

The study was preregistered on OSF and can be found here: osf.io/cfqm9. There were deviations from the preregistrations regarding various manners, which are described and justified below:

1. The analysis of the skin conductance response (SCR): For the SCR we did not find reasonable task effects. The paradigm was optimized for EEG and fear-potentiated startle (FPS) data, not SCR data, which may explain missing reasonable SCR response towards the US. Therefore, we decided not to interpret SCR data any further.
2. The reinforcement rate during generalization: It was preregistered that during the generalization phase, the CS+ would be paired with the US in 100% of the cases. This was changed to 80% reinforcement rate comparable to acquisition, to leave the threat experience of the CS+ unchanged.
3. Preprocessing of the FPS: The analysis section of the FPS has been updated to reflect the most current and accurate version of the current state of research. The prior version was outdated and, therefore, the analysis plan was updated. FPS data have only been analyzed in the updated way.
4. Hypothesis H3a (“all participants show fear extinction”) was added after the preregistration due to the fact that this hypothesis was forgotten and not preregistered by mistake.
5. For LPP, single-trial FPS, and shock (i.e., US) expectancy rating analyses, mixed-measured ANOVAs were pre-registered. Instead, a series of linear mixed-effects models were fitted for two reasons: First, to reduce the number of separate analyses. Second, to allow for simultaneous testing of both categorical and dimensional predictors, instead of separate ANOVAs (see appendix D).
6. Multiple additional exploratory analyses were added:
   1. An additional linear mixed-effects model was conducted for each outcome measure including the factor phase (i.e., habituation, acquisition, generalization, and extinction for shock expectancy rating as well as acquisition, generalization, and extinction for FPS and LPP data), stimulus cue (i.e., CS+ and CS- only), and group (i.e., OCD, SAD, SP, and control).
   2. Two mixed-measures ANOVAs were calculated with the between-subject factor group and the within-subject factor phase (i.e., acquisition and generalization for shock valence, and acquisition, generalization, and extinction for startle valence).
   3. Depressive symptoms using the sum score of the Beck Depression Inventory (BDI-II; Beck et al., 1996; Hautzinger et al., 2006) were added into the dimensional analyses as recent meta-analytic findings suggest an influence of depressive symptoms on altered fear learning (Kausche et al., 2025).
   4. Bayesian analyses for the main outcome measures to better parameterize our observed null effects.
   5. Individuals with any current anxiety- or stress-related disorder (i.e., anxiety disorders, OCD, PTSD) were compared to individuals without such diagnoses.
   6. Individuals with any current anxiety- or stress related disorder and current comorbid depression (i.e., current depressive episode or dysthymia) were compared to individuals with a current anxiety- or stress related disorder without a current comorbid depression, and individuals without such diagnoses.

**Appendix B**

At different stages of data collection, a range of questionnaires and diagnostic instruments were administered. Here, only the measures that are directly reported in the present study are presented. This approach aims to offer a clear and coherent clinical overview, without overloading the description with measures that are not relevant to the analyses shown.

Prior to the laboratory assessment, a screening was conducted by trained personnel during which the following interviews and questionnaires were administered:

- A screening-based version of the Structured Clinical Interview for DSM-5 Disorders – Clinical Version to assess presence of current mental disorders (SCID-5-CV; Beesdo-Baum et al., 2019; First et al., 2016)
- In the presence of obsessive-compulsive symptoms, the Yale-Brown Obsessive Compulsive Scale (Y-BOCS; semi-structured interview, 19 items, 5-point Likert scale 0–4; Goodman, 1989; Hand & Büttner-Westphal, 1991)
- The global assessment of functioning (GAF; American Psychiatric Association, 2000; DSM-IV Version)
- The severity of illness with the respective scale of the Clinical Global Impression (CGI; 7-point Liker scale 1–7; Guy, 1976)

During the laboratory assessment, prior to completing the battery of tasks, the following questionnaires were assessed:

- Anxious apprehension by the Penn State Worry Questionnaire (PSWQ; 16 items, 5-point Likert scale 1–5; Cronbach’s α = .94; Glöckner-Rist & Rist, 2006; Meyer et al., 1990)
- Anxious arousal by the respective subscale of the Mood and Anxiety Symptom Questionnaire (MASQ-AA; 17 items, 5-point Likert scale 1–5; Cronbach’s
  α = .88; Watson et al., 1995; Watson & Clark, 1991)
- Depression symptoms by the Beck Depression Inventory-II (BDI-II; 21 items,
  4-point Likert scale 0–3; Cronbach’s α = .95; Beck et al., 1996; Hautzinger et al., 2006)
- State and trait anxiety by the respective subscale of the State-Trait-Anxiety Inventory (STAI; 20 items each, 4-point Likert scale 1–4; State: Cronbach’s
  α = .95; Trait: Cronbach’s α = .95; Laux et al., 1981; Spielberger et al., 1983)
- Obsessive-compulsive symptoms by the Obsessive-Compulsive Inventory-Revised (OCI-R; 18 items, 5-point Likert scale 0–4; Cronbach’s α = .92; Foa et al., 2002; Gönner et al., 2007)
- Social anxiety symptoms by the self-report version of Liebowitz Social Anxiety Scale (LSAS-SR; 24 items for fear and avoidance each, 4-point Likert scale 0–3; Cronbach’s α = .98; Baker et al., 2002; Liebowitz, 1987; Stangier & Heidenreich, 2005)
- Specific phobia symptoms by the DSM-5 Severity Measure for Specific Phobia (SMSP; 10 items, 5-point Likert scale 0–4; Cronbach’s α = .86; Craske et al., 2013)

**Appendix C**

Color Coding of Stimuli

The bluest (HEX #2914cc) and greenest (HEX #14cc29) stimuli served as CS+ and CS-, counterbalanced across participants. The stimuli in between served as generalization stimuli (GS). One GS was most similar to the blue circle (HEX #2442a3), one most similar to the green circle (HEX #199e52), and one GS was the most ambiguous stimulus, incorporating equally blue and green color (HEX #1f707b; see table C-1).

| Stimulus | | Color Coding |
| --- | --- | --- |
|  | CS+ / CS- | HEX #2914cc |
|  | GS+ / GS- | HEX #2442a3 |
|  | GSU | HEX #1f707b |
|  | GS- / GS+ | HEX #199e52 |
|  | CS- / CS+ | HEX #14cc29 |

**Table C-1.** Overview of the stimuli used.

**Appendix D**

Results of the Pre-Registered Analyses

***D-1 Mixed-Measures ANOVAs for the Categorical Comparison of Participants with SAD (n = 39), Specific Phobia (n = 40), OCD (n = 38), and Controls (n = 39).***

ERPs, single-trial FPS, and US-expectancy ratings were analyzed using separate mixed-measures ANOVAs with the between-subject factor group with four levels (i.e., OCD, SAD, specific phobia, control) and the within-factor stimulus cue (i.e., CS+ and CS- for acquisition and extinction, and CS+, GS+, GSU, GS- and CS- for generalization). Following this, *t*-tests for separate group comparisons between the clinical groups and the control group respectively were calculated. Significant main or interaction effects were pursued using post-hoc planned comparisons. If sphericity assumption was violated, Greenhouse-Geiser (if Greenhouse-Geiser ε ≤ 0.75) or Huynh-Feldt (if Greenhouse-Geiser ε > 0.75) correction was applied.

**Table D-1-1**

*Results of the Mixed-Measures ANOVA for the Acquisition Phase.*

|  | *F* | *df* | $\eta_{p}^{2}$ | *p* |
| --- | --- | --- | --- | --- |
| Shock Expectancy Rating |  |  |  |  |
| Stimulus | 5345.88 | 1, 147 | .97 | **<.001** |
| Group | 2.23 | 3, 147 | .04 | .087 |
| Stimulus × Group | 3.51 | 3, 147 | .07 | **.017**^1^ |
| FPS |  |  |  |  |
| Stimulus | 38.53 | 1, 143 | .21 | **<.001** |
| Group | 0.66 | 3, 143 | .01 | .577 |
| Stimulus × Group | 0.28 | 3, 143 | .01 | .840 |
| LPP |  |  |  |  |
| Stimulus | 35.91 | 1, 145 | .20 | **<.001** |
| Group | 0.68 | 3, 145 | .01 | .565 |
| Stimulus × Group | .38 | 3, 145 | .01 | .767 |

*Note.* FPS = Fear-Potentiated Startle; LPP = Late Positive Potential; degrees of freedom are deviating for the outcome measures due to missing data. *p* < .05 are printed in bold.

^1^ Sidak corrected post-hoc pairwise comparisons revealed no significance (all *p*s > .053)

**Table D-1-2**

*Results of the Mixed-Measures ANOVA for the Generalization Phase.*

|  | *F* | *df* | $\eta_{p}^{2}$ | *p* |
| --- | --- | --- | --- | --- |
| Shock Expectancy Rating^1^ |  |  |  |  |
| Stimulus | 661.12 | 1.81, 266.31 | .82 | **<.001** |
| Group | 0.57 | 3, 147 | .01 | .634 |
| Stimulus × Group | 1.07 | 5.46, 266.31 | .02 | .380 |
| FPS^1^ |  |  |  |  |
| Stimulus | 45.65 | 3.74, 534.99 | .24 | **<.001** |
| Group | 0.37 | 3, 143 | .01 | .777 |
| Stimulus × Group | 0.52 | 11.22, 534.99 | .01 | .895 |
| LPP^1^ |  |  |  |  |
| Stimulus | 31.15 | 3.28, 476.22 | .18 | **<.001** |
| Group | 0.25 | 3, 145 | .01 | .861 |
| Stimulus × Group | 0.81 | 9.85, 476.22 | .02 | .619 |

*Note.* FPS = Fear-Potentiated Startle; LPP = Late Positive Potential; degrees of freedom are deviating for the outcome measures due to missing data. *p* < .05 are printed in bold.

^1^ Greenhouse-Geiser (if Greenhouse-Geiser ε ≤ 0.75) or Huynh-Feldt (if Greenhouse-Geiser ε > 0.75) correction was applied as Mauchly’s test indicated that the assumption of sphericity was violated (Shock Expectancy Rating: χ²(9) = 365.03, *p* = <.001, ε = .45; FPS = χ²(9) = 31.77, *p* = <.001, ɛ̃ = .94; LPP = χ²(9) = 69.16, *p* = <.001, ɛ̃ = .82).

**Table D-1-3**

*Results of the Mixed-Measures ANOVA for the Extinction Phase.*

|  | *F* | *df* | $\eta_{p}^{2}$ | *p* |
| --- | --- | --- | --- | --- |
| Shock Expectancy Rating |  |  |  |  |
| Stimulus | 153.51 | 1, 147 | .51 | **<.001** |
| Group | 1.28 | 3, 147 | .03 | .284 |
| Stimulus × Group | 0.53 | 3, 147 | .01 | .665 |
| FPS |  |  |  |  |
| Stimulus | 42.79 | 1, 143 | .23 | **<.001** |
| Group | 0.13 | 3, 143 | <.01 | .944 |
| Stimulus × Group | 0.09 | 3, 143 | <.01 | .968 |
| LPP |  |  |  |  |
| Stimulus | 5.63 | 1, 145 | .04 | **.019** |
| Group | 1.07 | 3, 145 | .02 | .366 |
| Stimulus × Group | 1.63 | 3, 145 | .03 | .185 |

*Note.* FPS = Fear-Potentiated Startle; LPP = Late Positive Potential; degrees of freedom are deviating for the outcome measures due to missing data. *p* < .05 are printed in bold.

***D-2 Results for the pairwise post-hoc stimuli comparisons during generalization for shock expectancy (Table 2-1), FPS (Table 2-2), and Late-Positive Potential (LPP; Table 2-3).***

**Table D-2-1**

*Pairwise Post-Hoc Stimuli Comparisons for Shock Expectancy Rating during Generalization.*

|  | vs. | *M* | *SE* | Difference | *SE* | *p* | *95% CI* |
| --- | --- | --- | --- | --- | --- | --- | --- |
| CS- |  | 6.63 | 1.21 |  |  |  |  |
|  | GS- |  |  | 0.45 | .92 | 1.00 | -2.16; 3.05 |
|  | GSU |  |  | -2.98 | 1.30 | .209 | -6.68; 0.71 |
|  | GS+ |  |  | -32.67 | 2.94 | **<.001** | -41.02; -24.33 |
|  | CS+ |  |  | -86.57 | 1.43 | **<.001** | -90.63; -85.51 |
| GS- |  | 6.19 | 1.10 |  |  |  |  |
|  | GSU |  |  | -3.43 | .90 | **.002** | -5.98; -0.88 |
|  | GS+ |  |  | -33.12 | 2.74 | **<.001** | -40.91; -25.33 |
|  | CS+ |  |  | -87.02 | 1.38 | **<.001** | -90.93; 83.10 |
| GSU |  | 9.62 | 1.43 |  |  |  |  |
|  | GS+ |  |  | -29.69 | 2.34 | **<.001** | -37.19; -22.19 |
|  | CS+ |  |  | -83.59 | 1.64 | **<.001** | -88.24; -78.94 |
| GS+ |  | 39.31 | 2.92 |  |  |  |  |
|  | CS+ |  |  | -53.90 | 3.06 | **<.001** | -62.59; -45.21 |
| CS+ |  | 93.20 | 0.78 |  |  |  |  |

*Note. p* < .05 are printed in bold.

**Table D-2-2**

*Pairwise Post-Hoc Stimuli Comparisons for FPS during Generalization.*

|  |  | *M* | *SE* | *Difference* | *SE* | *p* | *95% CI* |
| --- | --- | --- | --- | --- | --- | --- | --- |
| CS- |  | 48.23 | 0.29 |  |  |  |  |
|  | GS- |  |  | -.87 | .41 | .32 | -2.05; 0.31 |
|  | GSU |  |  | -.07 | .39 | 1.00 | -1.19: 1.04 |
|  | GS+ |  |  | -2.65 | .46 | **<.001** | -3.95; -1.34 |
|  | CS+ |  |  | -5.26 | .52 | **<.001** | -6.73; -3.79 |
| GS- |  | 49.10 | 0.32 |  |  |  |  |
|  | GSU |  |  | -.80 | .39 | .39 | -0.34; 1.93 |
|  | GS+ |  |  | -1.78 | .44 | **<.001** | -3.03; -0.53 |
|  | CS+ |  |  | -4.39 | .53 | **<.001** | -5.90; -2.88 |
| GSU |  | 48.30 | 0.31 |  |  |  |  |
|  | GS+ |  |  | -2.58 | .42 | **<.001** | -3.76; -1.39 |
|  | CS+ |  |  | -5.19 | .54 | **<.001** | -6.72; -3.66 |
| GS+ |  | 50.88 | 0.32 |  |  |  |  |
|  | CS+ |  |  | -2.61 | .52 | **<.001** | -4.08; -1.15 |
| CS+ |  | 53.49 | 0.39 |  |  |  |  |

*Note. p* < .05 are printed in bold

**Table D-2-3**

*Pairwise Post-Hoc Stimuli Comparisons for LPP during Generalization.*

|  |  | *M* | *SE* | *Difference* | *SE* | *p* | *95% CI* |
| --- | --- | --- | --- | --- | --- | --- | --- |
| CS- |  | .62 | .11 |  |  |  |  |
|  | GS- |  |  | -.06 | .13 | 1.00 | -0.42; 0.31 |
|  | GSU |  |  | -.26 | .13 | .34 | -0.61; 0.10 |
|  | GS+ |  |  | -.56 | .15 | **.002** | -0.98; -0.15 |
|  | CS+ |  |  | -1.52 | .20 | **<.001** | -2.07; -0.96 |
| GS- |  | .68 | .12 |  |  |  |  |
|  | GSU |  |  | -.20 | .13 | .77 | -0.58; 0.18 |
|  | GS+ |  |  | -.51 | .16 | **.02** | -0.97; -0.04 |
|  | CS+ |  |  | -1.46 | .20 | **<.001** | -2.02; -0.90 |
| GSU |  | .88 | .12 |  |  |  |  |
|  | GS+ |  |  | -.30 | .14 | .26 | -0.70; 0.09 |
|  | CS+ |  |  | -1.26 | .16 | **<.001** | -1.72; -0.80 |
| GS+ |  | 1.19 | .14 |  |  |  |  |
|  | CS+ |  |  | -.95 | .17 | **<.001** | -1.42; -0.48 |
| CS+ |  | 2.14 | .18 |  |  |  |  |

*Note. p* < .05 are printed in bold

***D-3 Analyses of Transdiagnostic Dimensional Symptom Measures.***

Since there is a substantial symptom overlap among different anxiety disorders and OCD, we also analyzed transdiagnostic dimensional symptom measures way by using the degree of anxious apprehension measured with the PSWQ, anxious arousal measured with the MASQ-AA, and depressive symptoms measured with the BDI-II. For this, hierarchical regressions were conducted, with the first model including the PSWQ and MASQ-AA and a second model, which was not pre-registered, additionally including the BDI-II.

**Table D-3-1**

*Hierarchical Multiple Linear Regression Models for Shock Expectancy Ratings during Acquisition.*

|  | *R²* | *F* | *df* | *p* | *B* | *β* | *t* | *p* |
| --- | --- | --- | --- | --- | --- | --- | --- | --- |
| CS+ | .09 | 6.83 | 2, 148 | **.001** |  |  |  |  |
| PSWQ |  |  |  |  | 0.05 | .08 | 0.79 | .431 |
| MASQ-AA |  |  |  |  | 0.26 | .24 | 2.38 | **.019** |
| CS+ | .09 | 0.00 | 1, 147 | 1.000 |  |  |  |  |
| PSWQ |  |  |  |  | 0.05 | .08 | 0.72 | .473 |
| MASQ-AA |  |  |  |  | 0.26 | .24 | 2.11 | **.037** |
| BDI-II |  |  |  |  | <-0.01 | .00 | 0.00 | 1.000 |
|  |  |  |  |  |  |  |  |  |
| CS- | <.01 | 0.17 | 2, 148 | .841 |  |  |  |  |
| PSWQ |  |  |  |  | 0.05 | .06 | 0.57 | .570 |
| MASQ-AA |  |  |  |  | -0.07 | -.05 | -0.47 | .642 |
| CS- | <.01 | <0.01 | 1, 147 | .948 |  |  |  |  |
| PSWQ |  |  |  |  | 0.06 | .06 | 0.55 | .586 |
| MASQ-AA |  |  |  |  | -0.07 | -.05 | -0.38 | .702 |
| BDI-II |  |  |  |  | -0.01 | -.01 | -0.07 | .948 |

*Notes. B* = unstandardised beta-coefficent; *β* = standardised beta-coefficient; PSWQ = Penn State Worry Questionnaire; MASQ-AA = anxious arousal subscale of the Mood and Anxiety Symptom Questionnaire; BDI-II = Beck Depression Inventory. Significant results (*p* < .05) are printed in bold. *N* = 151.

**Table D-3-2**

*Hierarchical Multiple Linear Regression Models for Shock Expectancy Ratings during Generalization.*

|  | *R²* | *F* | *df* | *p* | *B* | *β* | *t* | *p* |
| --- | --- | --- | --- | --- | --- | --- | --- | --- |
| CS+ | .02 | 1.27 | 2, 148 | .284 |  |  |  |  |
| PSWQ |  |  |  |  | 0.01 | .01 | 0.09 | .929 |
| MASQ-AA |  |  |  |  | 0.14 | .12 | 1.20 | .230 |
| CS+ | .02 | 0.01 | 1, 147 | .937 |  |  |  |  |
| PSWQ |  |  |  |  | <0.01 | .01 | 0.05 | .961 |
| MASQ-AA |  |  |  |  | 0.14 | .12 | 1.03 | .303 |
| BDI-II |  |  |  |  | 0.01 | .01 | 0.08 | .937 |
|  |  |  |  |  |  |  |  |  |
| GS+ | .03 | 2.29 | 2, 148 | .105 |  |  |  |  |
| PSWQ |  |  |  |  | -0.11 | -.04 | -0.42 | .672 |
| MASQ-AA |  |  |  |  | 0.84 | .20 | 1.92 | .057 |
| GS+ | .03 | 0.14 | 1, 147 | .708 |  |  |  |  |
| PSWQ |  |  |  |  | -0.07 | -.03 | -0.23 | .816 |
| MASQ-AA |  |  |  |  | 0.92 | .22 | 1.88 | .063 |
| BDI-II |  |  |  |  | -0.14 | -.05 | -0.38 | .708 |
|  |  |  |  |  |  |  |  |  |
| GSU | .01 | 0.39 | 2, 148 | .676 |  |  |  |  |
| PSWQ |  |  |  |  | 0.12 | .09 | 0.89 | .378 |
| MASQ-AA |  |  |  |  | -0.12 | -.06 | -0.58 | .565 |
| GSU | .01 | 0.11 | 1, 147 | .744 |  |  |  |  |
| PSWQ |  |  |  |  | 0.10 | .08 | 0.67 | .502 |
| MASQ-AA |  |  |  |  | -0.16 | -.08 | -0.66 | .509 |
| BDI-II |  |  |  |  | 0.06 | .04 | 0.33 | .744 |
|  |  |  |  |  |  |  |  |  |
| GS- | <.01 | 0.05 | 2, 148 | .952 |  |  |  |  |
| PSWQ |  |  |  |  | -0.03 | -.03 | -0.24 | .808 |
| MASQ-AA |  |  |  |  | 0.05 | .03 | 0.30 | .762 |
| GS- | .01 | 1.37 | 1, 147 | .244 |  |  |  |  |
| PSWQ |  |  |  |  | -0.08 | -.08 | -0.70 | .486 |
| MASQ-AA |  |  |  |  | -0.05 | -.03 | -0.26 | .794 |
| BDI-II |  |  |  |  | 0.17 | .14 | 1.17 | .244 |
|  |  |  |  |  |  |  |  |  |
| CS- | .02 | 1.15 | 2, 148 | .320 |  |  |  |  |
| PSWQ |  |  |  |  | 0.10 | .09 | 0.89 | .377 |
| MASQ-AA |  |  |  |  | 0.08 | .04 | 0.43 | .667 |
| CS- | .02 | 0.93 | 1, 147 | .336 |  |  |  |  |
| PSWQ |  |  |  |  | 0.05 | .05 | 0.42 | .677 |
| MASQ-AA |  |  |  |  | -0.01 | -.01 | -0.06 | .956 |
| BDI-II |  |  |  |  | 0.15 | .12 | 0.97 | .336 |

*Notes. B* = unstandardised beta-coefficent; *β* = standardised beta-coefficient; PSWQ = Penn State Worry Questionnaire; MASQ-AA = anxious arousal subscale of the Mood and Anxiety Symptom Questionnaire; BDI-II = Beck Depression Inventory. No significant results (*p* < .05). *N* = 151.

**Table D-3-3**

*Hierarchical Multiple Linear Regression Models for Shock Expectancy Ratings during Extinction.*

|  | *R²* | *F* | *df* | *p* | *B* | *β* | *t* | *p* |
| --- | --- | --- | --- | --- | --- | --- | --- | --- |
| CS+ | .05 | 3.84 | 2, 148 | **.024** |  |  |  |  |
| PSWQ |  |  |  |  | -0.07 | -.03 | -0.26 | .796 |
| MASQ-AA |  |  |  |  | 0.98 | .24 | 2.34 | **.020** |
| CS+ | .10 | 7.82 | 1, 147 | **.006** |  |  |  |  |
| PSWQ |  |  |  |  | -0.37 | -.15 | -1.38 | .170 |
| MASQ-AA |  |  |  |  | 0.40 | .10 | 0.86 | .390 |
| BDI-II |  |  |  |  | 0.98 | .32 | 2.80 | **.006** |
|  |  |  |  |  |  |  |  |  |
| CS- | .04 | 3.34 | 2, 148 | **.038** |  |  |  |  |
| PSWQ |  |  |  |  | -0.11 | -.06 | -0.57 | .573 |
| MASQ-AA |  |  |  |  | 0.73 | .24 | 2.34 | **0.21** |
| CS- | .09 | 6.73 | 1, 147 | **.010** |  |  |  |  |
| PSWQ |  |  |  |  | -0.32 | -.17 | -1.58 | .116 |
| MASQ-AA |  |  |  |  | 0.33 | .11 | 0.95 | .346 |
| BDI-II |  |  |  |  | 0.68 | .30 | 2.59 | **.010** |

*Notes. B* = unstandardised beta-coefficent; *β* = standardised beta-coefficient; PSWQ = Penn State Worry Questionnaire; MASQ-AA = anxious arousal subscale of the Mood and Anxiety Symptom Questionnaire; BDI-II = Beck Depression Inventory. Significant results (*p* < .05) are printed in bold. *N* = 151.

**Table D-3-4**

*Hierarchical Multiple Linear Regression Models for the Fear-Potentiated Startle during Acquisition.*

|  | *R²* | *F* | *df* | *p* | *B* | *β* | *t* | *p* |
| --- | --- | --- | --- | --- | --- | --- | --- | --- |
| CS+ | .03 | 1.86 | 2, 144 | .160 |  |  |  |  |
| PSWQ |  |  |  |  | 0.01 | .02 | 0.15 | .885 |
| MASQ-AA |  |  |  |  | 0.10 | .15 | 1.44 | .152 |
| CS+ | .03 | 0.05 | 1, 143 | .822 |  |  |  |  |
| PSWQ |  |  |  |  | <0.01 | .01 | 0.04 | .968 |
| MASQ-AA |  |  |  |  | 0.09 | .14 | 1.17 | .243 |
| BDI-II |  |  |  |  | 0.01 | .03 | 0.23 | .822 |
|  |  |  |  |  |  |  |  |  |
| CS- | <.01 | 0.10 | 2, 144 | .903 |  |  |  |  |
| PSWQ |  |  |  |  | -0.01 | -.04 | -0.42 | .675 |
| MASQ-AA |  |  |  |  | 0.01 | .01 | 0.12 | .903 |
| CS- | <.01 | 0.01 | 1, 143 | .909 |  |  |  |  |
| PSWQ |  |  |  |  | -0.01 | -.05 | -0.43 | .668 |
| MASQ-AA |  |  |  |  | <0.01 | .01 | 0.06 | .956 |
| BDI-II |  |  |  |  | 0.01 | .01 | 0.12 | .909 |

*Notes. B* = unstandardised beta-coefficent; *β* = standardised beta-coefficient; PSWQ = Penn State Worry Questionnaire; MASQ-AA = anxious arousal subscale of the Mood and Anxiety Symptom Questionnaire; BDI-II = Beck Depression Inventory. No significant results (*p* < .05). *N* = 147.

**Table D-3-5**

*Hierarchical Multiple Linear Regression Models for Fear-Potentiated Startle during Generalization.*

|  | *R²* | *F* | *df* | *p* | *B* | *β* | *t* | *p* |
| --- | --- | --- | --- | --- | --- | --- | --- | --- |
| CS+ | <.01 | 0.09 | 2, 144 | .910 |  |  |  |  |
| PSWQ |  |  |  |  | 0.01 | .04 | 0.39 | .698 |
| MASQ-AA |  |  |  |  | -0.02 | -.04 | -0.39 | .697 |
| CS+ | <.01 | 0.08 | 1, 143 | .777 |  |  |  |  |
| PSWQ |  |  |  |  | 0.01 | .03 | 0.24 | .812 |
| MASQ-AA |  |  |  |  | -0.03 | -.06 | -0.48 | .635 |
| BDI-II |  |  |  |  | 0.01 | .04 | 0.28 | .777 |
|  |  |  |  |  |  |  |  |  |
| GS+ | .01 | 0.96 | 2, 144 | .384 |  |  |  |  |
| PSWQ |  |  |  |  | -0.02 | -.08 | -0.75 | .458 |
| MASQ-AA |  |  |  |  | 0.07 | .14 | 1.38 | .169 |
| GS+ | .02 | 1.14 | 1, 143 | .288 |  |  |  |  |
| PSWQ |  |  |  |  | -0.01 | -.03 | -0.25 | .804 |
| MASQ-AA |  |  |  |  | 0.09 | .20 | 1.72 | .088 |
| BDI-II |  |  |  |  | -0.04 | -.13 | -1.07 | .288 |
|  |  |  |  |  |  |  |  |  |
| GSU | .02 | 1.40 | 2, 144 | .250 |  |  |  |  |
| PSWQ |  |  |  |  | -0.04 | -.14 | -1.31 | .193 |
| MASQ-AA |  |  |  |  | <-0.01 | <-.01 | -.03 | .973 |
| GSU | .02 | 0.42 | 1, 143 | .519 |  |  |  |  |
| PSWQ |  |  |  |  | -0.03 | -.11 | -0.93 | .354 |
| MASQ-AA |  |  |  |  | 0.01 | .03 | 0.27 | .790 |
| BDI-II |  |  |  |  | -0.03 | -.08 | -0.65 | .519 |
|  |  |  |  |  |  |  |  |  |
| GS- | <.01 | 0.28 | 2, 144 | .760 |  |  |  |  |
| PSWQ |  |  |  |  | -0.02 | -.07 | -0.70 | .483 |
| MASQ-AA |  |  |  |  | 0.03 | .06 | 0.61 | .540 |
| GS- | .02 | 1.94 | 1, 143 | .166 |  |  |  |  |
| PSWQ |  |  |  |  | -0.04 | -.14 | -1.21 | .229 |
| MASQ-AA |  |  |  |  | -0.01 | -.01 | -0.09 | .929 |
| BDI-II |  |  |  |  | 0.06 | .17 | 1.39 | .166 |
|  |  |  |  |  |  |  |  |  |
| CS- | <.01 | 0.21 | 2, 144 | .814 |  |  |  |  |
| PSWQ |  |  |  |  | -0.02 | -.06 | -0.61 | .540 |
| MASQ-AA |  |  |  |  | 0.01 | .02 | 0.22 | .823 |
| CS- | .05 | 6.36 | 1, 143 | **.013** |  |  |  |  |
| PSWQ |  |  |  |  | 0.01 | .05 | 0.45 | .652 |
| MASQ-AA |  |  |  |  | 0.07 | .16 | 1.36 | .176 |
| BDI-II |  |  |  |  | -0.09 | -.30 | -2.52 | **.013** |

*Notes. B* = unstandardised beta-coefficent; *β* = standardised beta-coefficient; PSWQ = Penn State Worry Questionnaire; MASQ-AA = anxious arousal subscale of the Mood and Anxiety Symptom Questionnaire; BDI-II = Beck Depression Inventory. Significant results (*p* < .05) are printed in bold. *N* = 147.

**Table D-3-6**

*Hierarchical Multiple Linear Regression Models for Fear-Potentiated Startle during Extinction.*

|  | *R²* | *F* | *df* | *p* | *B* | *β* | *t* | *p* |
| --- | --- | --- | --- | --- | --- | --- | --- | --- |
| CS+ | <.01 | 0.10 | 2, 144 | .904 |  |  |  |  |
| PSWQ |  |  |  |  | 0.02 | .05 | 0.45 | .654 |
| MASQ-AA |  |  |  |  | -0.02 | -.03 | -0.29 | .769 |
| CS+ | <.01 | .02 | 1, 143 | .898 |  |  |  |  |
| PSWQ |  |  |  |  | 0.01 | .04 | 0.36 | .721 |
| MASQ-AA |  |  |  |  | -0.02 | -.04 | -0.32 | .750 |
| BDI-II |  |  |  |  | 0.01 | .02 | 0.13 | .898 |
|  |  |  |  |  |  |  |  |  |
| CS- | .01 | 0.57 | 2, 144 | .569 |  |  |  |  |
| PSWQ |  |  |  |  | 0.01 | .03 | 0.29 | .773 |
| MASQ-AA |  |  |  |  | -0.05 | -.10 | -0.99 | .324 |
| CS- | .04 | 5.19 | 1, 143 | **.024** |  |  |  |  |
| PSWQ |  |  |  |  | -0.02 | -.07 | -0.66 | .513 |
| MASQ-AA |  |  |  |  | -0.10 | -.22 | -1.94 | .055 |
| BDI-II |  |  |  |  | 0.09 | .28 | 2.28 | **.024** |

*Notes. B* = unstandardised beta-coefficent; *β* = standardised beta-coefficient; PSWQ = Penn State Worry Questionnaire; MASQ-AA = anxious arousal subscale of the Mood and Anxiety Symptom Questionnaire; BDI-II = Beck Depression Inventory. Significant results (*p* < .05) are printed in bold. *N* = 147.

**Table D-3-7**

*Hierarchical Multiple Linear Regression Models for Late Positive Potential (Pz, 300-1000ms) during Acquisition.*

|  | *R²* | *F* | *df* | *p* | *B* | *β* | *t* | *p* |
| --- | --- | --- | --- | --- | --- | --- | --- | --- |
| CS+ | .01 | 0.55 | 2, 146 | .579 |  |  |  |  |
| PSWQ |  |  |  |  | <-0.01 | -.02 | -0.22 | .825 |
| MASQ-AA |  |  |  |  | -0.02 | -.07 | -0.69 | .494 |
| CS+ | .01 | <0.01 | 1, 145 | .998 |  |  |  |  |
| PSWQ |  |  |  |  | <-0.01 | -.02 | -0.20 | .840 |
| MASQ-AA |  |  |  |  | -0.02 | -.07 | -0.62 | .538 |
| BDI-II |  |  |  |  | <0.01 | <.01 | <0.01 | .998 |
|  |  |  |  |  |  |  |  |  |
| CS- | <.01 | 0.29 | 2, 146 | .748 |  |  |  |  |
| PSWQ |  |  |  |  | <-0.01 | -.01 | -0.09 | .929 |
| MASQ-AA |  |  |  |  | 0.01 | .07 | 0.66 | .511 |
| CS- | .01 | 0.39 | 1, 145 | .532 |  |  |  |  |
| PSWQ |  |  |  |  | <0.01 | .02 | 0.18 | .861 |
| MASQ-AA |  |  |  |  | 0.02 | .10 | 0.87 | .389 |
| BDI-II |  |  |  |  | -0.01 | -.08 | -.63 | .532 |

*Notes. B* = unstandardised beta-coefficent; *β* = standardised beta-coefficient; PSWQ = Penn State Worry Questionnaire; MASQ-AA = anxious arousal subscale of the Mood and Anxiety Symptom Questionnaire; BDI-II = Beck Depression Inventory. No significant results (*p* < .05). *N* = 149.

**Table D-3-8**

*Hierarchical Multiple Linear Regression Models for Late Positive Potential (Pz, 300-1000ms) during Generalization.*

|  | *R²* | *F* | *df* | *p* | *B* | *β* | *t* | *p* |
| --- | --- | --- | --- | --- | --- | --- | --- | --- |
| CS+ | .01 | 0.62 | 2, 146 | .542 |  |  |  |  |
| PSWQ |  |  |  |  | -0.02 | -.10 | -0.93 | .352 |
| MASQ-AA |  |  |  |  | <0.01 | .01 | 0.08 | .937 |
| CS+ | .01 | 0.04 | 1, 145 | .848 |  |  |  |  |
| PSWQ |  |  |  |  | -0.01 | -.09 | -0.77 | .443 |
| MASQ-AA |  |  |  |  | 0.01 | .02 | 0.15 | .878 |
| BDI-II |  |  |  |  | -0.01 | -.02 | -0.19 | .848 |
|  |  |  |  |  |  |  |  |  |
| GS+ | .02 | 1.19 | 2, 146 | .307 |  |  |  |  |
| PSWQ |  |  |  |  | -0.01 | -.08 | -0.77 | .444 |
| MASQ-AA |  |  |  |  | 0.03 | .16 | 1.53 | .128 |
| GS+ | .02 | 0.16 | 1, 145 | .690 |  |  |  |  |
| PSWQ |  |  |  |  | -0.01 | -.10 | -0.86 | .390 |
| MASQ-AA |  |  |  |  | 0.03 | .14 | 1.20 | .231 |
| BDI-II |  |  |  |  | 0.01 | .05 | 0.40 | .690 |
|  |  |  |  |  |  |  |  |  |
| GSU | .01 | 0.61 | 2, 146 | .546 |  |  |  |  |
| PSWQ |  |  |  |  | -0.01 | -.07 | -0.67 | .502 |
| MASQ-AA |  |  |  |  | 0.02 | .11 | 1.10 | .272 |
| GSU | .01 | 0.42 | 1, 145 | .518 |  |  |  |  |
| PSWQ |  |  |  |  | -0.01 | -.10 | -0.88 | .381 |
| MASQ-AA |  |  |  |  | 0.01 | .08 | 0.71 | .479 |
| BDI-II |  |  |  |  | 0.01 | .08 | 0.65 | .518 |
|  |  |  |  |  |  |  |  |  |
| GS- | <.01 | .027 | 2, 146 | .974 |  |  |  |  |
| PSWQ |  |  |  |  | <0.01 | <-.01 | -0.02 | .982 |
| MASQ-AA |  |  |  |  | <-0.01 | -.02 | -0.17 | .865 |
| GS- | .01 | 0.67 | 1, 145 | .414 |  |  |  |  |
| PSWQ |  |  |  |  | <-0.01 | -.04 | -0.36 | .722 |
| MASQ-AA |  |  |  |  | -0.01 | -.06 | -0.51 | .612 |
| BDI-II |  |  |  |  | 0.01 | .10 | 0.82 | .414 |
|  |  |  |  |  |  |  |  |  |
| CS- | .01 | 0.46 | 2, 146 | .636 |  |  |  |  |
| PSWQ |  |  |  |  | 0.01 | .08 | 0.78 | .437 |
| MASQ-AA |  |  |  |  | -0.02 | -.09 | -0.91 | .366 |
| CS- | .02 | 2.06 | 1, 145 | .154 |  |  |  |  |
| PSWQ |  |  |  |  | <0.01 | .01 | 0.13 | .900 |
| MASQ-AA |  |  |  |  | -0.03 | -.16 | -1.44 | .152 |
| BDI-II |  |  |  |  | 0.02 | .17 | 1.43 | .154 |

*Notes. B* = unstandardised beta-coefficent; *β* = standardised beta-coefficient; PSWQ = Penn State Worry Questionnaire; MASQ-AA = anxious arousal subscale of the Mood and Anxiety Symptom Questionnaire; BDI-II = Beck Depression Inventory. No significant results (*p* < .05). *N* = 149.

**Table D-3-9**

*Hierarchical Multiple Linear Regression Models for Late Positive Potential (Pz, 300-1000ms) during Extinction.*

|  | *R²* | *F* | *df* | *p* | *B* | *β* | *t* | *p* |
| --- | --- | --- | --- | --- | --- | --- | --- | --- |
| CS+ | .01 | 0.49 | 2, 146 | .613 |  |  |  |  |
| PSWQ |  |  |  |  | -0.01 | -.10 | -0.93 | .353 |
| MASQ-AA |  |  |  |  | 0.02 | .09 | 0.83 | .409 |
| CS+ | .02 | 2.34 | 1, 145 | .128 |  |  |  |  |
| PSWQ |  |  |  |  | -0.02 | -.17 | -1.48 | .140 |
| MASQ-AA |  |  |  |  | <0.01 | .01 | 0.09 | .933 |
| BDI-II |  |  |  |  | 0.03 | .18 | 1.53 | .128 |
|  |  |  |  |  |  |  |  |  |
| CS- | .02 | 1.29 | 2, 146 | .278 |  |  |  |  |
| PSWQ |  |  |  |  | -0.01 | -.08 | -0.77 | .445 |
| MASQ-AA |  |  |  |  | -0.01 | -.07 | -0.67 | .502 |
| CS- | .02 | 0.23 | 1, 145 | .636 |  |  |  |  |
| PSWQ |  |  |  |  | -0.01 | -.10 | -0.89 | .375 |
| MASQ-AA |  |  |  |  | -0.02 | -.09 | -0.81 | .418 |
| BDI-II |  |  |  |  | 0.01 | .06 | 0.48 | .636 |

*Notes. B* = unstandardised beta-coefficent; *β* = standardised beta-coefficient; PSWQ = Penn State Worry Questionnaire; MASQ-AA = anxious arousal subscale of the Mood and Anxiety Symptom Questionnaire; BDI-II = Beck Depression Inventory. No significant results (*p* < .05). *N* = 149.

**Appendix E**

Results of the Model for the Shock Expectancy Rating Including only the BDI-II as a Dimensional Measure.

The model for the shock expectancy rating during the generalization phase was computed without the BDI-II due to multicollinearity, influences of the BDI-II were checked separately (see table C-1 for model fit indices and results) as there were no a priori hypotheses generated for the influence of BDI-II. The following model was computed:

Shock Expectancy ~ stimulus cue + group + BDI-II + stimulus cue × group + stimulus cue × BDI-II + stimulus cue × group × BDI-II + (1 | ID)

**Table E-1**

*Model Fit Indices and Results of the Type III ANOVA with Satterthwaite’s Method for the Generalization Phase.*

|  | $R_{c}^{2}$ / $R_{m}^{2}$ | ICC | RMSE | σ | *F* | *df* | *p* | $R_{p}^{2}$ |
| --- | --- | --- | --- | --- | --- | --- | --- | --- |
| Shock Expectancy Rating | .791/.728 | 0.232 | 16.47 | 19.05 |  |  |  |  |
| Stimulus cue |  |  |  |  | 348.90 | 1, 572 | **<.001** | .153 |
| Group |  |  |  |  | 0.15 | 3, 143 | .932 | .001 |
| BDI-II |  |  |  |  | 0.30 | 1, 143 | .587 | .000 |
| Stimulus cue × Group |  |  |  |  | 0.89 | 12, 572 | .559 | .012 |
| Stimulus cue × BDI-II |  |  |  |  | 0.05 | 4, 572 | .996 | .001 |
| Stimulus cue × Group × BDI-II |  |  |  |  | 0.38 | 12, 572 | .969 | .005 |

*Note.* $R_{c}^{2}$ = Conditional *R²*; $R_{m}^{2}$ = Marginal *R²*; $R_{p}^{2}$ = Partial *R²*; BDI-II = Beck Depression Inventory. *p* < .05 are printed in bold.

**Appendix F**

Overview of Missing Data and Dropouts

For various reasons, participants included for each outcome measure differ (see table D-1). Overall, *N* = 163 were invited to the laboratory assessment. *n* = 5 participants were excluded as they did not finish the task. *n* = 2 participants were excluded as they met exclusion criteria. The final sample consisted of *n* = 156 participants (*n* = 39 control participants, *n* = 38 patients with OCD, *n* = 40 patients with specific phobia, *n* = 39 patients with SAD). Out of these, *n* = 5 participants were excluded for the rating, EEG, and FPS data as they did not report to have learned the association between the CS+ and the US (i.e., shock expectancy rating CS- > CS+). For EEG analysis, *n* = 2 participants had to be excluded as they were identified as outliers within the LPP data (i.e., mean LPP activity > 3 SD), which was traced back to technical malfunction and artificial EEG activity. For FPS analysis, *n* = 5 were excluded because startle analyses showed more than 66% zeros or missings.

**Table F-1**

*Number of Participants for each Outcome Measure*.

| N | OCD | SAD | PHOB | CON |
| --- | --- | --- | --- | --- |
| Overall | 38 | 39 | 40 | 39 |
| Rating | 37 | 37 | 39 | 38 |
| EEG | 36 | 37 | 38 | 38 |
| FPS | 35 | 34 | 39 | 38 |

*Note*. N = Number of participants; OCD = Obsessive-Compulsive Disorder; SAD = Social Anxiety Disorder; PHOB = Specific Phobia; CON = Controls; EEG = Electroencephalography; FPS = Fear-Potentiated Startle.

**Appendix G**

Distribution of Clinical Questionnaires across Groups*.*





**Figure G-1.** PSWQ = Penn State Worry Questionnaire; MASQ-AA = Anxious arousal subscale of the Mood and Anxiety Symptom Questionnaire; OCD = Obsessive-compulsive disorder; SAD = Social anxiety disorder; SP = Specific phobia; Con = Control group. *N* = 151.

**

**

**Figure G-2.** BDI-II = Beck Depression Inventory-II; OCI-R = Obsessive-Compulsive Inventory-Revised; OCD = Obsessive-compulsive disorder; SAD = Social anxiety disorder; SP = Specific phobia; Con = Control group. *N* = 151.

**

**

**Figure G-3.** LSAS-SR = self-report version of Liebowitz Social Anxiety Scale; SMPS = DSM-5 Severity Measure for Specific Phobia; OCD = Obsessive-compulsive disorder; SAD = Social anxiety disorder; SP = Specific phobia; Con = Control group. *N* = 151.

**

**

**Figure G-4.** STAI-T = Trait anxiety subscale of the State-Trait-Anxiety Inventory; STAI-S = State anxiety subscale of the State-Trait-Anxiety-Subscale; OCD = Obsessive-compulsive disorder; SAD = Social anxiety disorder; SP = Specific phobia; Con = Control group. *N* = 151.





**Figure G-5.** GAF = Global Assessment of Functioning (DSM-IV Version); OCD = Obsessive-compulsive disorder; SAD = Social anxiety disorder; SP = Specific phobia; Con = Control group. *N* = 151.

**Appendix H**

Results for Sidak corrected estimated marginal means (EMMs) post-hoc comparisons during generalization for shock expectancy (Table E-1), FPS (Table E-2), and Late-Positive Potential (LPP; Table E-3).

**Table H-1**

*Estimated Marginal Means Post-Hoc Stimuli Comparisons for Shock Expectancy Ratings during Generalization.*

|  |  | Estimate | *SE* | Df | *t* | *p* |
| --- | --- | --- | --- | --- | --- | --- |
| CS- |  |  |  |  |  |  |
|  | GS- | 0.41 | 2.05 | 596 | 0.20 | >.999 |
|  | GSU | -3.02 | 2.05 | 596 | -1.48 | .780 |
|  | GS+ | -32.68 | 2.05 | 596 | -15.97 | **<.001** |
|  | CS+ | -86.60 | 2.05 | 596 | -42.31 | **<.001** |
| GS- |  |  |  |  |  |  |
|  | GSU | -3.43 | 2.05 | 596 | -1.68 | .628 |
|  | GS+ | -33.09 | 2.05 | 596 | -16.17 | **<.001** |
|  | CS+ | -87.01 | 2.05 | 596 | -42.51 | **<.001** |
| GSU |  |  |  |  |  |  |
|  | GS+ | -29.66 | 2.05 | 596 | -14.49 | **<.001** |
|  | CS+ | -83.58 | 2.05 | 596 | -40.84 | **<.001** |
| GS+ |  |  |  |  |  |  |
|  | CS+ | -53.92 | 2.05 | 596 | -26.35 | **<.001** |

*Note. p* < .05 are printed in bold.

**Table H-2**

*Estimated Marginal Means Post-Hoc Stimuli Comparisons for Fear-Potentiated Startle during Generalization.*

|  |  | Estimate | *SE* | Df | *z* | *p* |
| --- | --- | --- | --- | --- | --- | --- |
| CS- |  |  |  |  |  |  |
|  | GS- | -2.67 | 1.91 | Inf | -1.40 | .827 |
|  | GSU | -0.12 | 1.97 | Inf | -0.06 | >.999 |
|  | GS+ | -9.41 | 1.89 | Inf | -4.97 | **<.001** |
|  | CS+ | -19.74 | 1.94 | Inf | -10.20 | **<.001** |
| GS- |  |  |  |  |  |  |
|  | GSU | 2.55 | 1.99 | Inf | 1.28 | .894 |
|  | GS+ | -6.74 | 1.92 | Inf | -3.51 | **. 005** |
|  | CS+ | -17.07 | 1.96 | Inf | -8.69 | **<.001** |
| GSU |  |  |  |  |  |  |
|  | GS+ | -9.29 | 1.98 | Inf | -4.69 | **<.001** |
|  | CS+ | -19.62 | 2.02 | Inf | -9.71 | **<.001** |
| GS+ |  |  |  |  |  |  |
|  | CS+ | -10.33 | 1.95 | Inf | -5.30 | **<.001** |

*Note.* Degrees-of-freedom method: asymptotic. *p* < .05 are printed in bold.

**Table H-3**

*Estimated Marginal Means Post-Hoc Stimuli Comparisons for Late Positive Potential during Generalization.*

|  |  | Estimate | *SE* | Df | *t* | *p* |
| --- | --- | --- | --- | --- | --- | --- |
| CS- |  |  |  |  |  |  |
|  | GS- | -0.07 | 0.17 | 564 | -0.42 | >.999 |
|  | GSU | -0.31 | 0.17 | 564 | -1.85 | .487 |
|  | GS+ | -0.57 | 0.17 | 564 | -3.41 | **.007** |
|  | CS+ | -1.49 | 0.17 | 564 | -8.87 | **<.001** |
| GS- |  |  |  |  |  |  |
|  | GSU | -0.24 | 0.17 | 564 | 1.43 | .808 |
|  | GS+ | -0.50 | 0.17 | 564 | 2.99 | **.029** |
|  | CS+ | -1.42 | 0.17 | 564 | -8.45 | **<.001** |
| GSU |  |  |  |  |  |  |
|  | GS+ | -0.26 | 0.17 | 564 | -1.56 | .721 |
|  | CS+ | -1.18 | 0.17 | 564 | -7.02 | **<.001** |
| GS+ |  |  |  |  |  |  |
|  | CS+ | -0.92 | 0.17 | 564 | -5.46 | **<.001** |

*Note. p* < .05 are printed in bold.

**Appendix I**

In the following, results of the exploratory analyses will be displayed.

***I-1 Phase Effect.***

**Table I-1-1**

*Model Fit Indices and Results of the Type III Satterthwaite’s Method.*

|  | $R_{c}^{2}$ / $R_{m}^{2}$ | ICC | RMSE | σ | *F* | *df* | *p* | $R_{p}^{2}$ | 95% CI |
| --- | --- | --- | --- | --- | --- | --- | --- | --- | --- |
| Shock expectancy rating | .765 / .722 | 0.153 | 18.83 | 19.83 |  |  |  |  |  |
| Stimulus cue |  |  |  |  | 2045.39 | 1, 1029 | **<.001** | .000 | .000, .004 |
| Phase |  |  |  |  | 201.60 | 3, 1029 | **<.001** | .266 | .228, .308 |
| Group |  |  |  |  | 3.39 | 3, 147 | **.020** | .009 | .003, .026 |
| Stimulus cue × Phase |  |  |  |  | 336.19 | 3, 1029 | **<.001** | .177 | .142, .218 |
| Stimulus cue × Group |  |  |  |  | 0.70 | 3, 1029 | .556 | .001 | .000, .010 |
| Phase × Group |  |  |  |  | 1.01 | 9, 1029 | .431 | .008 | .005, .029 |
| Stimulus cue × Phase × Group |  |  |  |  | 0.45 | 9, 1029 | .906 | .003 | .003, .022 |
| FPS^1^ | .654 / .042 | 0.638 | 40.49 | 41.15 |  |  |  |  |  |
| Stimulus cue |  |  |  |  | 176.13 | 1, 4998 | **<.001** | .001 | .000, .004 |
| Phase |  |  |  |  | 43.26 | 2, 4998 | **<.001** | .002 | .000, .006 |
| Group |  |  |  |  | 1.87 | 3, 143 | .138 | .013 | .007, .020 |
| Stimulus cue × Phase |  |  |  |  | 4.31 | 2, 4998 | **.014** | .000 | .000, .003 |
| Stimulus cue × Group |  |  |  |  | 1.88 | 3, 4998 | .130 | .001 | .000, .004 |
| Phase × Group |  |  |  |  | 2.00 | 6, 4998 | .062 | .001 | .001, .005 |
| Stimulus cue × Phase × Group |  |  |  |  | 0.42 | 6, 2998 | .866 | .001 | .000, .004 |
| LPP | .436 / .133 | 0.349 | 1.22 | 1.32 |  |  |  |  |  |
| Stimulus cue |  |  |  |  | 99.38 | 1, 725 | **<.001** | .009 | .001, .025 |
| Phase |  |  |  |  | 29.96 | 2, 725 | **<.001** | .000 | .000, .010 |
| Group |  |  |  |  | 0.38 | 3, 145 | .765 | .001 | .000, .013 |
| Stimulus cue × Phase |  |  |  |  | 14.95 | 2, 725 | **<.001** | .013 | .003, .033 |
| Stimulus cue × Group |  |  |  |  | 0.27 | 3, 725 | .845 | .001 | .000, .013 |
| Phase × Group |  |  |  |  | 1.30 | 6, 725 | .257 | .003 | .002, .022 |
| Stimulus cue × Phase × Group |  |  |  |  | 1.07 | 6, 725 | .380 | .006 | .003, .028 |

*Note.* $R_{c}^{2}$ = Conditional *R²*; $R_{m}^{2}$ = Marginal *R²*; $R_{p}^{2}$ = Partial *R²*; CI = Confidence interval; FPS = Fear-potentiated startle; LPP = Late positive potential. Degrees of freedom are deviating for the outcome measures due to missing data. The model included stimulus cue (i.e., CS+ and CS-), group (i.e., OCD, SAD, SP, control), and phase (i.e., habitation, acquisition, generalization, and extinction for shock expectancy ratings as well as acquisition, generalization, and extinction for FPS and LPP) as well as their full interaction. Significant effects (*p* < .05) are printed in bold.

^1^ single trial data were used for the FPS.

**Table I-1-2**

*Phase Post Hoc Comparisons for Shock Expectancy Ratings using EMMs for CS- and CS+ Separately to Investigate the Significant Stimulus Cue × Phase Interaction.*

| Shock expectancy  rating | | Estimate | *SE* | df | *t* | *p* |
| --- | --- | --- | --- | --- | --- | --- |
| CS- | |  |  |  |  |  |
|  | Hab vs. Acq | 8.64 | 2.28 | 1029 | 3.77 | **.001** |
|  | Hab vs. Gen | 8.82 | 2.28 | 1029 | 3.86 | **<.001** |
|  | Hab vs. Ext | 0.25 | 2.28 | 1029 | 0.11 | >.999 |
|  | Acq vs. Gen | 0.18 | 2.28 | 1029 | 0.08 | >.999 |
|  | Acq vs. Ext | -8.39 | 2.28 | 1029 | -3.68 | **.002** |
|  | Gen vs. Ext | -8.57 | 2.28 | 1029 | -3.76 | **.001** |
| CS+ | |  |  |  |  |  |
|  | Hab vs. Acq | -75.42 | 2.28 | 1029 | -33.04 | **<.001** |
|  | Hab vs. Gen | -75.86 | 2.28 | 1029 | -33.23 | **<.001** |
|  | Hab vs. Ext | -29.94 | 2.28 | 1029 | -13.11 | **<.001** |
|  | Acq vs. Gen | -0.44 | 2.28 | 1029 | -0.19 | >.999 |
|  | Acq vs. Ext | 45.49 | 2.28 | 1029 | 19.27 | **<.001** |
|  | Gen vs. Ext | 45.93 | 2.28 | 1029 | 20.12 | **<.001** |

*Note.* Hab = Habituation; Acq = Acquisition; Gen = Generalization; Ext = Extinction. Significant comparisons (*p* < .05) are printed in bold.

**Table I-1-3**

*Phase Post Hoc Comparisons for Shock Expectancy Ratings using EMMs for all Phases Separately to Investigate the Significant Stimulus Cue × Phase Interaction.*

| Shock expectancy  rating | | Estimate | *SE* | df | *t* | *p* |
| --- | --- | --- | --- | --- | --- | --- |
| Habituation | |  |  |  |  |  |
|  | CS- vs. CS+ | -1.89 | 2.28 | 1029 | -0.83 | .409 |
| Acquisition | |  |  |  |  |  |
|  | CS- vs. CS+ | -85.95 | 2.28 | 1029 | -37.65 | **<.001** |
| Generalization | |  |  |  |  |  |
|  | CS- vs. CS+ | -86.57 | 2.28 | 1029 | -37.92 | **<.001** |
| Extinction | |  |  |  |  |  |
|  | CS- vs. CS+ | -32.07 | 2.28 | 1029 | -14.05 | **<.001** |

*Note.* Significant comparisons (*p* < .05) are printed in bold.

**Table I-1-4**

*Phase Post Hoc Comparisons for the Fear-Potentiated Startle using EMMs for CS- and CS+ Separately to Investigate the Significant Stimulus Cue × Phase Interaction.*

| Fear-potentiated  startle | | Estimate | *SE* | df | *z* | *p* |
| --- | --- | --- | --- | --- | --- | --- |
| CS- | |  |  |  |  |  |
|  | Acq vs. Gen | 12.14 | 1,96 | Inf | 6.19 | **<.001** |
|  | Acq vs. Ext | 12.56 | 1.97 | Inf | 6.37 | **<.001** |
|  | Gen vs. Ext | 0.43 | 1.97 | Inf | 0.22 | .995 |
| CS+ | |  |  |  |  |  |
|  | Acq vs. Gen | 5.12 | 2.03 | Inf | 2.52 | **.035** |
|  | Acq vs. Ext | 12.93 | 1.98 | Inf | 6.52 | **<.001** |
|  | Gen vs. Ext | 7.82 | 2.03 | Inf | 3.84 | **<.001** |

*Note.* Phase post hoc comparisons for single-trial fear-potentiated startle data using EMMs for CS- and CS+ separately to investigate the significant stimulus cue × phase interaction. Acq = Acquisition; Gen = Generalization; Ext = Extinction. Degrees-of-freedom method: asymptotic. Significant comparisons (*p* < .05) are printed in bold.

**Table I-1-5**

*Phase Post Hoc Comparisons for the Fear-Potentiated Startle using EMMs for all Phases Separately to Investigate the Significant Stimulus Cue × Phase Interaction.*

| Fear-potentiated  startle | | Estimate | *SE* | df | *z* | *p* |
| --- | --- | --- | --- | --- | --- | --- |
| Acquisition | |  |  |  |  |  |
|  | CS- vs. CS+ | -13.0 | 1.98 | Inf | -6.61 | **<.001** |
| Generalization | |  |  |  |  |  |
|  | CS- vs. CS+ | -20.1 | 2.02 | Inf | -9.94 | **<.001** |
| Extinction | |  |  |  |  |  |
|  | CS- vs. CS+ | -12.7 | 1.98 | Inf | -6.40 | **<.001** |

*Note.* Degrees-of-freedom method: asymptotic. Significant comparisons (*p* < .05) are printed in bold.

**Table I-1-6**

*Phase Post Hoc Comparisons for the Late Positive Potential using EMMs for CS- and CS+ Separately to Investigate the Significant Stimulus Cue × Phase Interaction.*

| Late positive  potential | | Estimate | *SE* | df | *t* | *p* |
| --- | --- | --- | --- | --- | --- | --- |
| CS- | |  |  |  |  |  |
|  | Acq vs. Gen | -0.09 | 0.15 | 725 | -0.57 | .919 |
|  | Acq vs. Ext | 0.16 | 0.15 | 725 | 1.07 | .637 |
|  | Gen vs. Ext | 0.25 | 0.15 | 725 | 1.64 | .276 |
| CS+ | |  |  |  |  |  |
|  | Acq vs. Gen | -0.81 | 0.15 | 725 | -5.31 | **<.001** |
|  | Acq vs. Ext | 0.61 | 0.15 | 725 | 3.99 | **<.001** |
|  | Gen vs. Ext | 1.43 | 0.15 | 725 | 9.30 | **<.001** |

*Note***.** Acq = Acquisition; Gen = Generalization; Ext = Extinction. Significant comparisons (*p* < .05) are printed in bold.

**Table I-1-7**

*Phase Post Hoc Comparisons for the Late Positive Potential using EMMs for all Phases Separately to Investigate the Significant Stimulus Cue × Phase Interaction.*

| Late positive  potential | | Estimate | *SE* | df | *t* | *p* |
| --- | --- | --- | --- | --- | --- | --- |
| Acquisition | |  |  |  |  |  |
|  | CS- vs. CS+ | -0.79 | 0.15 | 725 | -5.15 | **<.001** |
| Generalization | |  |  |  |  |  |
|  | CS- vs. CS+ | -1.52 | 0.15 | 725 | -9.89 | **<.001** |
| Extinction | |  |  |  |  |  |
|  | CS- vs. CS+ | -0.34 | 0.15 | 725 | -2.23 | **.026** |

*Note*. Significant comparisons (*p* < .05) are printed in bold.

***I-2 Bayesian Analyses***

**Table I-2-1**

*Bayesian Repeated Measures ANOVA for Shock Expectancy during Acquisition.*

| Models | P(M) | P(M\|data) | BF _M_ | BF _10_ | error % |
| --- | --- | --- | --- | --- | --- |
| Stimulus | 0.200 | 0.458 | 3.384 | 1.000 |  |
| Stimulus + Group + Stimulus*Group | 0.200 | 0.419 | 2.889 | 0.915 | 6.419 |
| Stimulus + Group | 0.200 | 0.122 | 0.557 | 0.267 | 1.497 |
| Null model (incl. subject) | 0.200 | <0.001 | <0.001 | <0.001 | 1.012 |
| Group | 0.200 | <0.001 | <0.001 | <0.001 | 1.359 |

*Notes.* All models include subject.

**Table I-2-2**

*Bayesian Repeated Measures ANOVA for Shock Expectancy during Generalization.*

| Models | P(M) | P(M\|data) | BF _M_ | BF _10_ | error % |
| --- | --- | --- | --- | --- | --- |
| Stimulus | 0.200 | 0.969 | 123.205 | 1.000 |  |
| Stimulus + Group | 0.200 | 0.031 | 0.129 | 0.032 | 2.669 |
| Stimulus + Group + Stimulus*Group | 0.200 | <0.001 | 0.001 | <0.001 | 2.451 |
| Null model (incl. subject) | 0.200 | <0.001 | <0.001 | <0.001 | 2.348 |
| Group | 0.200 | <0.001 | <0.001 | <0.001 | 2.409 |

*Notes.* All models include subject.

**Table I-2-3**

*Bayesian Repeated Measures ANOVA for Shock Expectancy during Extinction.*

| Models | P(M) | P(M\|data) | BF _M_ | BF _10_ | error % |
| --- | --- | --- | --- | --- | --- |
| Stimulus | 0.200 | 0.824 | 18.734 | 1.000 |  |
| Stimulus + Group | 0.200 | 0.165 | 0.792 | 0.201 | 1.401 |
| Stimulus + Group + Stimulus*Group | 0.200 | 0.011 | 0.043 | 0.013 | 2.998 |
| Null model (incl. subject) | 0.200 | <0.001 | <0.001 | <0.001 | 0.937 |
| Group | 0.200 | <0.001 | <0.001 | <0.001 | 1.086 |

*Notes.* All models include subject.

**Table I-2-4**

*Bayesian Repeated Measures ANOVA for Fear-Potentiated Startle during Acquisition.*

| Models | P(M) | P(M\|data) | BF _M_ | BF _10_ | error % |
| --- | --- | --- | --- | --- | --- |
| Stimulus | 0.200 | 0.949 | 74.186 | 1.000 |  |
| Stimulus + Group | 0.200 | 0.049 | 0.205 | 0.051 | 1.516 |
| Stimulus + Group + Stimulus*Group | 0.200 | 0.003 | 0.010 | 0.003 | 1.775 |
| Null model (incl. subject) | 0.200 | <0.001 | <0.001 | <0.001 | 1.164 |
| Group | 0.200 | <0.001 | <0.001 | <0.001 | 1.286 |

*Notes.* All models include subject.

**Table I-2-5**

*Bayesian Repeated Measures ANOVA for Fear-Potentiated Startle during Generalization.*

| Models | P(M) | P(M\|data) | BF _M_ | BF _10_ | error % |
| --- | --- | --- | --- | --- | --- |
| Stimulus | 0.200 | 0.990 | 389.745 | 1.000 |  |
| Stimulus + Group | 0.200 | 0.010 | 0.041 | 0.010 | 1.055 |
| Stimulus + Group + Stimulus*Group | 0.200 | <0.001 | <0.001 | <0.001 | 1.004 |
| Null model (incl. subject) | 0.200 | <0.001 | <0.001 | <0.001 | 0.485 |
| Group | 0.200 | <0.001 | <0.001 | <0.001 | 0.892 |

*Notes.* All models include subject.

**Table I-2-6**

*Bayesian Repeated Measures ANOVA for Fear-Potentiated Startle during Extinction.*

| Models | P(M) | P(M\|data) | BF _M_ | BF _10_ | error % |
| --- | --- | --- | --- | --- | --- |
| Stimulus | 0.200 | 0.972 | 138.805 | 1.000 |  |
| Stimulus + Group | 0.200 | 0.027 | 0.111 | 0.028 | 2.256 |
| Stimulus + Group + Stimulus*Group | 0.200 | 0.001 | 0.004 | 0.001 | 3.119 |
| Null model (incl. subject) | 0.200 | <0.001 | <0.001 | <0.001 | 1.986 |
| Group | 0.200 | <0.001 | <0.001 | <0.001 | 2.354 |

*Notes.* All models include subject.

**Table I-2-7**

*Bayesian Repeated Measures ANOVA for Late Positive Potential during Acquisition.*

| Models | P(M) | P(M\|data) | BF _M_ | BF _10_ | error % |
| --- | --- | --- | --- | --- | --- |
| Stimulus | 0.200 | 0.898 | 35.344 | 1.000 |  |
| Stimulus + Group | 0.200 | 0.096 | 0.427 | 0.107 | 1.955 |
| Stimulus + Group + Stimulus*Group | 0.200 | 0.005 | 0.021 | 0.006 | 2.395 |
| Null model (incl. subject) | 0.200 | <0.001 | <0.001 | <0.001 | 1.595 |
| Group | 0.200 | <0.001 | <0.001 | <0.001 | 1.698 |

*Notes.* All models include subject.

**Table I-2-8**

*Bayesian Repeated Measures ANOVA for Late Positive Potential during Generalization.*

| Models | P(M) | P(M\|data) | BF _M_ | BF _10_ | error % |
| --- | --- | --- | --- | --- | --- |
| Stimulus | 0.200 | 0.969 | 126.654 | 1.000 |  |
| Stimulus + Group | 0.200 | 0.030 | 0.126 | 0.031 | 0.985 |
| Stimulus + Group + Stimulus*Group | 0.200 | <0.001 | <0.001 | <0.001 | 0.830 |
| Null model (incl. subject) | 0.200 | <0.001 | <0.001 | <0.001 | 0.429 |
| Group | 0.200 | <0.001 | <0.001 | <0.001 | 0.583 |

*Notes.* All models include subject.

**Table I-2-9**

*Bayesian Repeated Measures ANOVA for Late Positive Potential during Extinction.*

| Models | P(M) | P(M\|data) | BF _M_ | BF _10_ | error % |
| --- | --- | --- | --- | --- | --- |
| Stimulus | 0.200 | 0.553 | 4.946 | 1.000 |  |
| Null model (incl. subject) | 0.200 | 0.332 | 1.990 | 0.601 | 1.480 |
| Stimulus + Group | 0.200 | 0.062 | 0.264 | 0.112 | 2.132 |
| Group | 0.200 | 0.037 | 0.155 | 0.067 | 1.687 |
| Stimulus + Group + Stimulus*Group | 0.200 | 0.016 | 0.064 | 0.029 | 2.514 |

*Notes.* All models include subject.

***I-3-1*** ***Results for the Linear Mixed-Effect Models*** ***for the Comparison of Individuals with any Current Anxiety- or Stress-Related Disorder (i.e., Anxiety Disorders, OCD, PTSD; n = 117) to those without (n = 39).***

**Table I-3-1-1**

*Model Fit Indices and Results of the Type III ANOVA with Satterthwaite’s Method for Models for the Acquisition Phase for the Comparison of Individuals with any Current Anxiety- or Stress-Related Disorder (i.e., Anxiety Disorders, OCD, PTSD) to those without.*

|  | $R_{c}^{2}$ / $R_{m}^{2}$ | ICC | RMSE | σ | *F* | *df* | *p* | $R_{p}^{2}$ |
| --- | --- | --- | --- | --- | --- | --- | --- | --- |
| Shock Expectancy Rating | .944 / .940 | 0.076 | 10.03 | 10.47 |  |  |  |  |
| Stimulus cue |  |  |  |  | 3882.59 | 1, 149 | **<.001** | .806 |
| Group |  |  |  |  | 0.49 | 1, 149 | .487 | .004 |
| Stimulus cue × Group |  |  |  |  | 0.74 | 1, 149 | 0.391 | .002 |
| FPS^1^ | .683 / .009 | 0.681 | 38.20 | 39.87 |  |  |  |  |
| Stimulus cue |  |  |  |  | 25.59 | 1, 1598.93 | **<.001** | .002 |
| Group |  |  |  |  | 0.07 | 1, 144.51 | .787 | .002 |
| Stimulus cue × Group |  |  |  |  | 1.74 | 1, 1598.93 | .187 | .001 |
| LPP | .517 / .063 | 0.485 | 0.92 | 1.13 |  |  |  |  |
| Stimulus cue |  |  |  |  | 31.49 | 1, 147 | **<.001** | .026 |
| Group |  |  |  |  | 0.70 | 1, 147 | .404 | .001 |
| Stimulus cue × Group |  |  |  |  | 0.51 | 1, 147 | .478 | .001 |

*Note.* $R_{c}^{2}$ = Conditional *R²*; $R_{m}^{2}$ = Marginal *R²*; $R_{p}^{2}$ = Partial *R²*; FPS = Fear-Potentiated Startle; LPP = Late Positive Potential; degrees of freedom are deviating for the outcome measures due to missing data. *p* < .05 are printed in bold.
^1^ single trial data were used for the FPS.

**Table I-3-1-2**

*Model Fit Indices and Results of the Type III ANOVA with Satterthwaite’s Method for Models for the Generalization Phase for the Comparison of Individuals with any Current Anxiety- or Stress-Related Disorder (i.e., Anxiety Disorders, OCD, PTSD) to those without.*

|  | $R_{c}^{2}$ / $R_{m}^{2}$ | ICC | RMSE | σ | *F* | *df* | *p* | $R_{p}^{2}$ |
| --- | --- | --- | --- | --- | --- | --- | --- | --- |
| Shock Expectancy Rating | .791 / .729 | 0.230 | 16.73 | 17.95 |  |  |  |  |
| Stimulus cue |  |  |  |  | 497.47 | 4, 596 | **<.001** | .432 |
| Group |  |  |  |  | 0.53 | 1, 149 | .467 | .001 |
| Stimulus cue × Group |  |  |  |  | 0.11 | 4, 596 | .978 | .001 |
| FPS^1^ | .648 / .017 | .642 | 38.75 | 39.48 |  |  |  |  |
| Stimulus cue |  |  |  |  | 24.57 | 4, 3977.00 | **<.001** | .006 |
| Group |  |  |  |  | 1.10 | 1, 144.7 | .295 | .005 |
| Stimulus cue × Group |  |  |  |  | 0.66 | 4, 3977.00 | .623 | .001 |
| LPP | .399 / .105 | 0.328 | 1.25 | 1.35 |  |  |  |  |
| Stimulus cue |  |  |  |  | 25.69 | 4, 588 | **<.001** | .042 |
| Group |  |  |  |  | 0.08 | 1, 147 | .776 | .000 |
| Stimulus cue × Group |  |  |  |  | 0.90 | 4, 588 | .464 | .004 |

*Note.* $R_{c}^{2}$ = Conditional *R²*; $R_{m}^{2}$ = Marginal *R²*; $R_{p}^{2}$ = Partial *R²*; FPS = Fear-Potentiated Startle; LPP = Late Positive Potential; degrees of freedom are deviating for the outcome measures due to missing data. *p* < .05 are printed in bold.
^1^ single trial data were used for the FPS.

**Table I-3-1-3**

*Model Fit Indices and Results of the Type III ANOVA with Satterthwaite’s Method for Models for the Extinction Phase for the Comparison of Individuals with any Current Anxiety- or Stress-Related Disorder (i.e., Anxiety Disorders, OCD, PTSD) to those without.*

|  | $R_{c}^{2}$ / $R_{m}^{2}$ | ICC | RMSE | σ | *F* | *df* | *p* | $R_{p}^{2}$ |
| --- | --- | --- | --- | --- | --- | --- | --- | --- |
| Shock Expectancy Rating | .579 / .217 | 0.462 | 18.44 | 22.45 |  |  |  |  |
| Stimulus cue |  |  |  |  | 113.53 | 1, 149 | **<.001** | .069 |
| Group |  |  |  |  | 0.57 | 1, 149 | .450 | .001 |
| Stimulus cue × Group |  |  |  |  | 0.04 | 1, 149 | .850 | .000 |
| FPS^1^ | .637 / .010 | 0.634 | 38.989 | 40.69 |  |  |  |  |
| Stimulus cue |  |  |  |  | 31.86 | 1, 1589.24 | **<.001** | .006 |
| Group |  |  |  |  | 0.38 | 1, 144.38 | .538 | .006 |
| Stimulus cue × Group |  |  |  |  | 0.16 | 1, 1589.24 | .692 | .000 |
| LPP | .320 / .027 | 0.301 | 1.08 | 1.24 |  |  |  |  |
| Stimulus cue |  |  |  |  | 1.58 | 1, 147 | .211 | .000 |
| Group |  |  |  |  | 1.94 | 1, 147 | .166 | .015 |
| Stimulus cue × Group |  |  |  |  | 2.55 | 1, 147 | .113 | .006 |

*Note.* $R_{c}^{2}$ = Conditional *R²*; $R_{m}^{2}$ = Marginal *R²*; $R_{p}^{2}$ = Partial *R²*; FPS = Fear-Potentiated Startle; LPP = Late Positive Potential; degrees of freedom are deviating for the outcome measures due to missing data. *p* < .05 are printed in bold.
^1^ single trial data were used for the FPS.

***I-3-2*** ***Results for the Mixed-Measures ANOVAs for the Comparison of Individuals with any Current Anxiety- or Stress-Related Disorder (i.e., Anxiety Disorders, OCD, PTSD; n = 117) to those without (n = 39).***

**Table I-3-2-1**

*Results of the Mixed-Measures ANOVA for Acquisition Phase for the Comparison of Individuals with any Current Anxiety- or Stress-Related Disorder (i.e., Anxiety Disorders, OCD, PTSD) to those without.*

|  | *F* | *df* | $\eta_{p}^{2}$ | *p* |
| --- | --- | --- | --- | --- |
| Shock Expectancy Rating |  |  |  |  |
| Stimulus | 3882.59 | 1, 149 | .96 | **<.001** |
| Group | 0.49 | 1, 149 | <.01 | .487 |
| Stimulus × Group | 0.74 | 1, 149 | .01 | .391 |
| FPS |  |  |  |  |
| Stimulus | 27.16 | 1, 145 | .16 | **<.001** |
| Group | 0.53 | 1, 145 | <.01 | .468 |
| Stimulus × Group | 0.14 | 1, 145 | <.01 | .707 |
| LPP |  |  |  |  |
| Stimulus | 31.49 | 1, 147 | .18 | **<.001** |
| Group | 0.70 | 1, 147 | .01 | .404 |
| Stimulus × Group | 0.51 | 1, 147 | <.01 | .478 |

*Note.* FPS = Fear-Potentiated Startle; LPP = Late Positive Potential; degrees of freedom are deviating for the outcome measures due to missing data. *p* < .05 are printed in bold.

**Table I-3-2-2**

*Results of the Mixed-Measures ANOVA for the Generalization Phase for the Comparison of Individuals with any Current Anxiety- or Stress-Related Disorder (i.e., Anxiety Disorders, OCD, PTSD) to those without.*

|  | *F* | *df* | $\eta_{p}^{2}$ | *p* |
| --- | --- | --- | --- | --- |
| Shock Expectancy Rating^1^ |  |  |  |  |
| Stimulus | 497.47 | 1.81, 268.93 | .77 | **<.001** |
| Group | 0.53 | 1, 149 | <.01 | .467 |
| Stimulus × Group | 0.11 | 1.81, 268.93 | <.01 | .875 |
| FPS^1^ |  |  |  |  |
| Stimulus | 29.67 | 3.69, 535.59 | .17 | **<.001** |
| Group | 0.67 | 1, 145 | .01 | .414 |
| Stimulus × Group | 0.91 | 3.69, 535.59 | .01 | .449 |
| LPP^1^ |  |  |  |  |
| Stimulus | 25.69 | 3.24, 475.79 | .15 | **<.001** |
| Group | 0.08 | 1, 147 | <.01 | .776 |
| Stimulus × Group | 0.90 | 3.24, 475.79 | .01 | .448 |

*Note.* FPS = Fear-Potentiated Startle; LPP = Late Positive Potential; degrees of freedom are deviating for the outcome measures due to missing data. *p* < .05 are printed in bold.

^1^ Greenhouse-Geiser (if Greenhouse-Geiser ε ≤ 0.75) or Huynh-Feldt (if Greenhouse-Geiser ε > 0.75) correction was applied as Mauchly’s test indicated that the assumption of sphericity was violated (Shock Expectancy Rating: χ²(9) = 368.80, *p* = <.001, ε = .45; FPS = χ²(9) = 31.75, *p* = <.001, ɛ̃ = .92; LPP = χ²(9) = 70.25, *p* = <.001, ɛ̃ = .81).

**Table I-3-2-3**

*Results of the Mixed-Measures ANOVA for the Extinction Phase for the Comparison of Individuals with any Current Anxiety- or Stress-Related Disorder (i.e., Anxiety Disorders, OCD, PTSD) to those without.*

|  | *F* | *df* | $\eta_{p}^{2}$ | *p* |
| --- | --- | --- | --- | --- |
| Shock Expectancy Rating |  |  |  |  |
| Stimulus | 133.53 | 1, 149 | .43 | **<.001** |
| Group | 0.58 | 1, 149 | <.01 | .450 |
| Stimulus × Group | 0.04 | 1, 149 | <.01 | .850 |
| FPS |  |  |  |  |
| Stimulus | 33.84 | 1, 145 | .19 | **<.001** |
| Group | 0.04 | 1, 145 | <.01 | .847 |
| Stimulus × Group | 0.05 | 1, 145 | <.01 | .825 |
| LPP |  |  |  |  |
| Stimulus | 1.58 | 1, 147 | .01 | .211 |
| Group | 1.94 | 1, 147 | .01 | .166 |
| Stimulus × Group | 2.55 | 1, 147 | .02 | .113 |

*Note.* FPS = Fear-Potentiated Startle; LPP = Late Positive Potential; degrees of freedom are deviating for the outcome measures due to missing data. *p* < .05 are printed in bold.

***I-4-1*** ***Results for the Linear Mixed-Effect Models*** ***for the Comparison of Individuals with a Current Anxiety- or Stress Related Disorder with a Current Comorbid Depression (i.e., Current Depressive Episode or Dysthymia; n = 24), to Individuals without a Current Comorbid Depression (n = 93), and Individuals without any Anxiety-Related Diagnosis (n = 39)***

**Table I-4-1-1**

*Model Fit Indices and Results of the Type III ANOVA with Satterthwaite’s Method for Models for the Acquisition Phase for the Comparison of Individuals with a Current Anxiety- or Stress Related Disorder with a Current Comorbid Depression (i.e., Current Depressive Episode or Dysthymia), to Individuals without a Current Comorbid Depression, and Individuals without.*

|  | $R_{c}^{2}$ / $R_{m}^{2}$ | ICC | RMSE | σ | *F* | *df* | *p* | $R_{p}^{2}$ |
| --- | --- | --- | --- | --- | --- | --- | --- | --- |
| Shock Expectancy Rating | .944 / .940 | 0.069 | 10.06 | 10.50 |  |  |  |  |
| Stimulus cue |  |  |  |  | 3769.44 | 1, 148 | **<.001** | .806 |
| Group |  |  |  |  | 1.39 | 2, 148 | .252 | .006 |
| Stimulus cue × Group |  |  |  |  | 0.47 | 2, 148 | .624 | .003 |
| FPS^1^ | .686 / .011 | 0.682 | 38.15 | 39.83 |  |  |  |  |
| Stimulus cue |  |  |  |  | 41.90 | 1, 1598.37 | **<.001** | .002 |
| Group |  |  |  |  | 0.16 | 2, 143.33 | .855 | .002 |
| Stimulus cue × Group |  |  |  |  | 3.07 | 1, 1598.31 | **.047** | .003 |
| LPP | .519 / .081 | 0.477 | 0.92 | 1.13 |  |  |  |  |
| Stimulus cue |  |  |  |  | 23.94 | 1, 146 | **<.001** | .026 |
| Group |  |  |  |  | 2.15 | 2, 146 | .120 | .006 |
| Stimulus cue × Group |  |  |  |  | 0.64 | 2, 146 | .531 | .003 |

*Note.* $R_{c}^{2}$ = Conditional *R²*; $R_{m}^{2}$ = Marginal *R²*; $R_{p}^{2}$ = Partial *R²*; FPS = Fear-Potentiated Startle; LPP = Late Positive Potential; degrees of freedom are deviating for the outcome measures due to missing data. *p* < .05 are printed in bold.
^1^ single trial data were used for the FPS.

**Table I-4-1-2**

*Model Fit Indices and Results of the Type III ANOVA with Satterthwaite’s Method for Models for the Generalization Phase for the Comparison of Individuals with a Current Anxiety- or Stress Related Disorder with a Current Comorbid Depression (i.e., Current Depressive Episode or Dysthymia), to Individuals without a Current Comorbid Depression, and Individuals without.*

|  | $R_{c}^{2}$ / $R_{m}^{2}$ | ICC | RMSE | σ | *F* | *df* | *p* | $R_{p}^{2}$ |
| --- | --- | --- | --- | --- | --- | --- | --- | --- |
| Shock Expectancy Rating | .792 / .730 | 0.228 | 16.67 | 17.95 |  |  |  |  |
| Stimulus cue |  |  |  |  | 468.72 | 4, 592 | **<.001** | .433 |
| Group |  |  |  |  | 1.42 | 2, 148 | .245 | .010 |
| Stimulus cue × Group |  |  |  |  | 0.61 | 8, 592 | .771 | .005 |
| FPS^1^ | .649 / .019 | 0.643 | 38.74 | 39.49 |  |  |  |  |
| Stimulus cue |  |  |  |  | 24.33 | 4, 3973.00 | **<.001** | .006 |
| Group |  |  |  |  | 0.66 | 2, 143.70 | .517 | .005 |
| Stimulus cue × Group |  |  |  |  | 0.54 | 8, 3973.00 | .830 | .001 |
| LPP | .402 / .107 | 0.330 | 1.24 | 1.35 |  |  |  |  |
| Stimulus cue |  |  |  |  | 21.76 | 4, 584 | **<.001** | .042 |
| Group |  |  |  |  | 0.09 | 2, 146 | .918 | .000 |
| Stimulus cue × Group |  |  |  |  | 0.88 | 8, 584 | .536 | .007 |

*Note.* $R_{c}^{2}$ = Conditional *R²*; $R_{m}^{2}$ = Marginal *R²*; $R_{p}^{2}$ = Partial *R²*; FPS = Fear-Potentiated Startle; LPP = Late Positive Potential; degrees of freedom are deviating for the outcome measures due to missing data. *p* < .05 are printed in bold.
^1^ single trial data were used for the FPS.

**Table I-4-1-3**

*Model Fit Indices and Results of the Type III ANOVA with Satterthwaite’s Method for Models for the Extinction Phase for the Comparison of Individuals with a Current Anxiety- or Stress Related Disorder with a Current Comorbid Depression (i.e., Current Depressive Episode or Dysthymia), to Individuals without a Current Comorbid Depression, and Individuals without.*

|  | $R_{c}^{2}$ / $R_{m}^{2}$ | ICC | RMSE | σ | *F* | *df* | *p* | $R_{p}^{2}$ |
| --- | --- | --- | --- | --- | --- | --- | --- | --- |
| Shock Expectancy Rating | .578 / .255 | 0.433 | 18.63 | 22.53 |  |  |  |  |
| Stimulus cue |  |  |  |  | 110.80 | 1, 148 | **<.001** | .071 |
| Group |  |  |  |  | 5.82 | 2, 148 | **.004** | .030 |
| Stimulus cue × Group |  |  |  |  | 0.02 | 2, 148 | .980 | .000 |
| FPS^1^ | .639 / .011 | 0.635 | 38.97 | 40.69 |  |  |  |  |
| Stimulus cue |  |  |  |  | 23.71 | 1, 1588.67 | **<.001** | .006 |
| Group |  |  |  |  | 0.29 | 2, 143.47 | .751 | .004 |
| Stimulus cue × Group |  |  |  |  | 0.62 | 2, 1588.60 | .539 | .001 |
| LPP | .324 / .038 | 0.316 | 1.06 | 1.23 |  |  |  |  |
| Stimulus cue |  |  |  |  | 8.22 | 1, 146 | .**005** | .000 |
| Group |  |  |  |  | 1.02 | 2, 146 | .363 | .019 |
| Stimulus cue × Group |  |  |  |  | 3.79 | 2, 146 | .**025** | .018 |

*Note.* $R_{c}^{2}$ = Conditional *R²*; $R_{m}^{2}$ = Marginal *R²*; $R_{p}^{2}$ = Partial *R²*; FPS = Fear-Potentiated Startle; LPP = Late Positive Potential; degrees of freedom are deviating for the outcome measures due to missing data. *p* < .05 are printed in bold.
^1^ single trial data were used for the FPS.

***I-4-2*** ***Results for the Mixed-Measures ANOVAs for the Comparison of Individuals with a Current Anxiety- or Stress Related Disorder with a Current Comorbid Depression (i.e., Current Depressive Episode or Dysthymia; n = 24), to Individuals without a Current Comorbid Depression (n = 93), and Individuals without any Anxiety-Related Diagnosis (n = 39)***

**Table I-4-2-1**

*Results of the Mixed-Measures ANOVA for the Acquisition Phase for the Comparison of Individuals with a Current Anxiety- or Stress Related Disorder with a Current Comorbid Depression (i.e., Current Depressive Episode or Dysthymia), to Individuals without a Current Comorbid Depression, and Individuals without.*

|  | *F* | *df* | $\eta_{p}^{2}$ | *p* |
| --- | --- | --- | --- | --- |
| Shock Expectancy Rating |  |  |  |  |
| Stimulus | 3769.44 | 1, 148 | .96 | **<.001** |
| Group | 1.39 | 2, 148 | .02 | .252 |
| Stimulus × Group | 0.47 | 2, 148 | .01 | .624 |
| FPS |  |  |  |  |
| Stimulus | 37.20 | 1, 144 | .21 | **<.001** |
| Group | 0.32 | 2, 144 | <.01 | .729 |
| Stimulus × Group | 1.43 | 2, 144 | .02 | .243 |
| LPP |  |  |  |  |
| Stimulus | 23.94 | 1, 146 | .14 | **<.001** |
| Group | 2.15 | 2, 146 | .03 | .120 |
| Stimulus × Group | 0.64 | 2, 146 | .01 | .531 |

*Note.* FPS = Fear-Potentiated Startle; LPP = Late Positive Potential; degrees of freedom are deviating for the outcome measures due to missing data. *p* < .05 are printed in bold.

**Table I-4-2-2**

*Results of the Mixed-Measures ANOVA for the Generalization Phase for the Comparison of Individuals with a Current Anxiety- or Stress Related Disorder with a Current Comorbid Depression (i.e., Current Depressive Episode or Dysthymia), to Individuals without a Current Comorbid Depression, and Individuals without.*

|  | *F* | *df* | $\eta_{p}^{2}$ | *p* |
| --- | --- | --- | --- | --- |
| Shock Expectancy Rating^1^ |  |  |  |  |
| Stimulus | 468.72 | 1.79, 264.22 | .76 | **<.001** |
| Group | 1.42 | 2, 148 | .02 | .245 |
| Stimulus × Group | 0.61 | 3.57, 264.22 | .01 | .638 |
| FPS^1^ |  |  |  |  |
| Stimulus | 28.14 | 3.72, 535.86 | .16 | **<.001** |
| Group | 1.21 | 2, 144 | .02 | .301 |
| Stimulus × Group | 1.21 | 7.44, 535.86 | .02 | .294 |
| LPP^1^ |  |  |  |  |
| Stimulus | 21.76 | 3.26, 475.23 | .13 | **<.001** |
| Group | 0.09 | 2, 146 | <.01 | .918 |
| Stimulus × Group | 0.88 | 6.51, 475.23 | .01 | .519 |

*Note.* FPS = Fear-Potentiated Startle; LPP = Late Positive Potential; degrees of freedom are deviating for the outcome measures due to missing data. *p* < .05 are printed in bold.

^1^ Greenhouse-Geiser (if Greenhouse-Geiser ε ≤ 0.75) or Huynh-Feldt (if Greenhouse-Geiser ε > 0.75) correction was applied as Mauchly’s test indicated that the assumption of sphericity was violated (Shock Expectancy Rating: χ²(9) = 372.85, *p* = <.001, ε = .45; FPS = χ²(9) = 31.64, *p* = <.001, ɛ̃ = .93; LPP = χ²(9) = 69.68, *p* = <.001, ɛ̃ = .81).

**Table I-4-2-3**

*Results of the Mixed-Measures ANOVA for the Extinction Phase.*

|  | *F* | *df* | $\eta_{p}^{2}$ | *p* |
| --- | --- | --- | --- | --- |
| Shock Expectancy Rating |  |  |  |  |
| Stimulus | 110.80 | 1, 148 | .43 | **<.001** |
| Group | 5.82 | 2, 148 | .07 | **.004** |
| Stimulus × Group | 0.02 | 2, 148 | <.01 | .980 |
| FPS |  |  |  |  |
| Stimulus | 24.51 | 1, 144 | .15 | **<.001** |
| Group | 0.02 | 2, 144 | <.01 | .982 |
| Stimulus × Group | 1.11 | 2, 144 | .02 | .334 |
| LPP |  |  |  |  |
| Stimulus | 8.22 | 1, 146 | .05 | **.005** |
| Group | 1.02 | 2, 146 | .01 | .363 |
| Stimulus × Group | 3.79 | 2, 146 | .05 | **.025** |

*Note.* FPS = Fear-Potentiated Startle; LPP = Late Positive Potential; degrees of freedom are deviating for the outcome measures due to missing data. *p* < .05 are printed in bold.

**References**

American Psychiatric Association. (2000). *Diagnostic and statistical manual of mental disorders (DSM-IV)*. American Psychiatric Association.

Baker, S. L., Heinrichs, N., Kim, H.-J., & Hofmann, S. G. (2002). The Liebowitz social anxiety scale as a self-report instrument: A preliminary psychometric analysis. *Behaviour Research and Therapy*, *40*, 701–715. <https://doi.org/10.1016/S0005-7967(01)00060-2>

Beck, A. T., Steer, R. A., & Brown, G. (1996). *Beck Depression Inventory–II*. Psychological Corporation. <https://doi.org/10.1037/t00742-000>

Beesdo-Baum, K., Zaudig, M., & Wittchen, H.-U. (2019). *Strukturiertes Klinisches Interview für DSM-5-Störungen—Klinische Version (German adaptation of SCID-5-CV)*. Hogrefe.

Craske, M., Wittchen, H., Bogels, S., Stein, M., Adrews, G., & Lebeu, R. (2013). *Severity Measure for Specific Phobia—Adult*. American Psychiatric Association.

First, M. B., Williams, J. B. W., Karg, R. S., & Spitzer, R. L. (2016). *SCID-5-CV: Structured clinical interview for DSM-5 disorders - clinician version*. American Psychiatric Association Publishing.

Foa, E. B., Huppert, J. D., Leiberg, S., Langner, R., Kichic, R., Hajcak, G., & Salkovskis, P. M. (2002). The Obsessive-Compulsive Inventory: Development and validation of a short version. *Psychological Assessment*, *14*, 485–496. <https://doi.org/10.1037/1040-3590.14.4.485>

Glöckner-Rist, A., & Rist, F. (2006). Deutsche Version des Penn State Worry Questionnaire (PSWQ-d). *Zusammenstellung sozialwissenschaftlicher Items und Skalen (ZIS)*. <https://doi.org/10.6102/ZIS219>

Gönner, S., Leonhart, R., & Ecker, W. (2007). Das Zwangsinventar OCI-R - die deutsche Version des Obsessive-Compulsive Inventory-Revised. *PPmP – Psychotherapie Psychosomatik Medizinische Psychologie*, *57*, 395–404. <https://doi.org/10.1055/s-2007-970894>

Goodman, W. K. (1989). The Yale-Brown Obsessive Compulsive Scale: I. Development, Use, and Reliability. *Archives of General Psychiatry*, *46*, 1006. <https://doi.org/10.1001/archpsyc.1989.01810110048007>

Guy, W. (1976). Clinical Global Impressions (CGI) scale [Database record]. APA PsycTests. <https://doi.org/10.1037/t48216-000>

Hand, I., & Büttner-Westphal, H. (1991). Die Yale-Brown Obsessive Compulsive Scale (Y-BOCS): Ein halbstrukturiertes Interview zur Beurteilung des Schweregrades von Denk- und Handlungszwängen. *Verhaltenstherapie*, *1*, 223–225. <https://doi.org/10.1159/000257972>

Hautzinger, M., Keller, F., & Kühner, C. (2006). *Beck Depressions-Inventar (BDI-II)*. Harcourt Test Services.

Kausche, F. M., Carsten, H. P., Sobania, K. M., & Riesel, A. (2025). Fear and safety learning in anxiety- and stress-related disorders: An updated meta-analysis. *Neuroscience & Biobehavioral Reviews*, *169*, 105983. <https://doi.org/10.1016/j.neubiorev.2024.105983>

Laux, L., Glanzmann, P., & Spielberger, C. D. (1981). *Das State-Trait-Angstinventar (STAI)*. Beltz.

Liebowitz, M. R. (1987). Social Phobia. *Modern Problems of Pharmacipsychiatry*, *22*, 141–173. <https://doi.org/10.1159/000414022>

Meyer, T. J., Miller, M. L., Metzger, R. L., & Borkovec, T. D. (1990). Development and validation of the penn state worry questionnaire. *Behaviour Research and Therapy*, *28*, 487–495. <https://doi.org/10.1016/0005-7967(90)90135-6>

Spielberger, C. D., Gorsuch, R. L., Lushene, R., Vagg, P. R., & Jacobs, G. A. (1983). *Manual for the State-Trait Anxiety Inventory (Form Y)*. Consulting Psychologists Press.

Stangier, U., & Heidenreich, T. (2005). Die Liebowitz Soziale Angst Skala (LSAS). In *Collegium Internationale Psychiatriae Scalarum*.

Watson, D. (2005). Rethinking the mood and anxiety disorders: A quantitative hierarchical model for DSM-V. *Journal of Abnormal Psychology*, *114*, 522–536. <https://doi.org/10.1037/0021-843X.114.4.522>

Watson, D., & Clark, L. A. (1991). *The Mood and Anxiety Symptom Questionnaire*. University of Iowa.
